# Contact in context: How land use histories, sociocultural practices, and animal profiles shape human-animal contact and zoonotic pathogen exposure in a fragmented forest of the Democratic Republic of Congo

**DOI:** 10.1371/journal.pntd.0013823

**Published:** 2026-10-26

**Authors:** Romain Duda, Victor Narat, Thibaut Langlois, Robert Monfura, Marc Allassonnière Tang, Placide Mbala, Claude Monghiemo, Tamara Giles-Vernick

**Affiliations:** 1 CEFE, Univ Montpellier, CNRS, EPHE, IRD, Montpellier, France; 2 Eco-anthropologie, MNHN, CNRS, Université Paris Cité, Paris, France; 3 MIVEGEC, IRD, CNRS, University of Montpellier, Montpellier, France; 4 ONG Mbou-Mon-Tour, Nkala, Democratic Republic of the Congo; 5 Unité de Virologie, Institut National de Recherche Biomédicale, Kinshasa, Democratic Republic of the Congo; 6 Anthropology and Ecology of Disease Emergence Unit, Institut Pasteur-Université Paris Cité, Paris, France; Universidad Tecnológica Equinoccial: Universidad UTE, ECUADOR

## Abstract

Pathogenic spillovers from animals into humans have catalyzed epidemics throughout history. They result from multiple factors. One such factor, human-animal contact, remains poorly understood. Current studies often neglect variability in human-animal engagements across ecological zones and the broader processes bringing people, animals, and pathogens into engagement. We investigated factors and longer-term processes shaping human-animal contacts and indirectly, potential exposures to zoonotic pathogens in a fragmentated forest. We conducted our mixed-methods investigation in three villages along an ecological gradient of forest fragmentation in the Democratic Republic of Congo (DRC), a hotspot of biodiversity and disease emergence. Twenty-four village participants recorded their daily activities and contacts with animal species over five months. We developed a cluster analysis to categorize classes of animals according to type and frequency of contact and conducted transects to estimate animal species abundance according to village proximity. We then tested the influence of species abundance, human activities, gender, and village on contact frequency. We also undertook ethnographic and ethnohistorical interviews and observations to explore changing human-animal relations. Participants had physical and environmental contact with 61 animal species, which clustered into three classes according to their contact profiles. Relative abundance of animal species was associated with contact frequency, but its effects were weak. Our ethnohistorical and anthropological analyses, however, situated these contacts within their longer-term processes and sociocultural practices that our models could not capture. The animals with which participants had most frequent contact were those whose abundance and accessibility resulted from decades of land use change and hunting intensification. Several abundant species were rarely contacted because local categorizations of animals as *ngeyi* (“disgusting”) suppressed contacts that proximity alone would have predicted. Human-animal contact profiles are thus historically produced and socioculturally mediated; they cannot be derived exclusively from species ecology, land cover, or demographic variables.

## Author summary

Pathogenic spillovers from animals into humans have catalyzed epidemics throughout history. These spillovers result from many factors, although one, human-animal contact, is poorly understood. Studies of contact rarely account for the variability of human-animal interactions or for the longer-term changes shaping engagements between people, animals, and pathogens. Our mixed-methods study explored factors and longer-term processes affecting human-animal contacts and indirectly, potential risks of exposure to zoonotic pathogens in a fragmented forest of the Democratic Republic of Congo.

Over five months, participants recorded contacts with 61 different animal species. We grouped these species into three different classes according to how frequent and in what ways people encountered them. Animal abundance predicted some contacts, but historical changes and sociocultural practices accounted for much of the rest. Decades of land use change and intensive hunting shaped which animals are now abundant and accessible near villages, whereas local understandings of certain animals as “disgusting” suppressed contact that proximity alone would have predicted.

Human-animal contacts are thus historically produced and socioculturally mediated. They cannot be anticipated from ecology alone. We recommend that future studies examine a wider array of contacts and situate them in their historical contexts.

## Introduction

Pathogenic spillovers from animals into human populations (anthropozoonoses) have long constituted a threat to public health, resulting in the transmission of several neglected tropical diseases (NTDs) and in some cases, catalyzing epidemics and pandemics [1,2]. Recent mpox and COVID-19 pandemics, for instance, have had their beginnings in nonhuman animals, but their pathways into human populations are complex, shaped by multiple factors, including phylogenetic proximity between hosts, a pathogen’s adaptive capacity, and animal ecologies and behaviors that may intersect with human practices to shape cross-species contacts [3]. Anthropogenic transformations, from climatic and resulting ecological changes, to habitat fragmentation, demographic expansion, hunting, butchering and wildlife trade, all shape human-animal contact [4–6]. Human-animal contact, although a critical factor in facilitating zoonotic spillovers, is still poorly understood at a fine scale. Prior research has tended to focus on large scale changes, from intensive agricultural production to commercialized animal production to deforestation, that occasion biodiversity loss and modify patterns of human-animal contact [7–9]. Nevertheless, zoonotic spillovers are not only the consequence of broad scale changes, but also the result of specific human-animal-pathogen interactions at specific sites and moments [10,11].

These specificities, however, have not been fully tackled in literature on human-animal contact. Certain studies have not defined or used “contact” consistently [12]. Large-scale explanations for and mechanisms of spillover, moreover, do not account for granular human-animal interactions; they thus neglect why zones experiencing anthropogenic disturbance do not all produce traceable zoonotic spillovers. Granular, small-scale investigations shed light on a range of reported human-animal contacts at times a limited group of animals (nonhuman primates); a few have productively explored human contacts with a wide range of pre-determined animal species to address the complex pathways that pathogens may follow in host spillovers [13–15]. Although a recent study evaluated contact frequency to elucidate the types and frequencies of human-human and human-animal contacts [16], most studies still neglect granular insight into the rich contexts of these contact events, the variability of human-animal engagements, and the broader historical, socio-cultural, economic, and ecological processes and practices bringing people, animals, and pathogens into engagement.

Generating deeper insight into human risks of zoonotic transmission necessitates that we expand our scope and methods to undertake a socio-ecological investigation. More broadly, complex relations among undomesticated and domesticated animals, human societies, and pathogens can shape zoonotic transmission pathways [17–19]. Interspecies contact can result from relations of predation, competition, mutualism, or avoidance, from overlapping ecological niches and sharing of resources and habitat, as well as coincidental nocturnal or diurnal activities. Species diversity and abundance can further influence contacts and indirectly pathogenic transmission [20]; large-scale climatic and ecological transformations may intersect with smaller scale ecological changes, which in turn can shape species distribution, density, and behavior, thus affecting interspecies contacts [21,22].

This article therefore asks what factors, conditions, and longer-term processes have shaped human-animal contacts and thus potential exposures to pathogens in a fragmented landscape? To tackle this question, we conducted a study on the Congo basin forest edge of the Democratic Republic of Congo (DRC), a hotspot of biodiversity and disease emergence. Our mixed-methods investigation brought together ecological and anthropological tools to explore human-animal contacts at a local scale. Our study analyzed quantitatively and qualitatively the diversity of domesticated, commensal, and undomesticated species with which human inhabitants had contact, as well as types and frequencies of these human-animal contacts. We explored comparatively whether animal abundance, human activities, village sites, or gender affected contact frequencies. We also situated this contact analysis in broader historical changes in land use, hunting and trapping, and animal production drawing on ethnographic observations and local oral histories. This article thus offers a broad analysis of contact and the historical, sociocultural processes shaping it, to depict human exposures to animals and indirectly potential pathogens in a fragmented habitat in Central Africa.

## Materials and methods

### Ethics and research authorization

The research protocol, information notice, and consent forms were evaluated by and received ethical approvals from the Institut Pasteur Institutional Review Board (Decision No. IRB2018-08) and the National Committee of Health Ethics (Comité National d’Éthique de la Santé, CNES) of the Democratic Republic of Congo (Decision no. ESP/CE/2019 and ESP/CE/045/2021). All potential participants received written and oral descriptions of the study, procedures for participation, and participant rights. To obtain informed consent, Romain Duda and Robert Monfura read and explained the information notice describing the study and data collection. Literate participants provided written informed consent. The few participants unable to read or write provided oral consent. This study is registered on clinical trials database (n°NCT04012164). Before beginning our study, we presented all study aims and methods to village chiefs for their authorization to conduct our research.

### Study sites and study period

We conducted field research from March to August 2019 and in September 2021 in three villages (here anonymized as villages A, B and C) located in the Bolobo Territory, Maï-Ndombe Province, DRC (Fig 1). Situated along the southwestern edge of the Congo basin forest, this area is characterized by a fragmented forest-savanna mosaic, composed of lowland rainforest and herbaceous and woodland savannas [23]. Characteristics of the three villages are summarized in Table 1.

**Table 1:** Characteristics of the three villages targeted in this study.

Village	A*	B	C
Human population	830 (2021)	1005 (2021)	582 (2021)
Surface area of village territory** (km^2^)	NA	135.6	94.3
Human density (ind./km^2^)	NA	7.4	6.2
Forest cover*** (%)	24	55	66
Edge density index**** (m/ha)	228	93	58

* Village A falls under the territorial authority of another village, so we cannot calculate the area of the village territory or human density.

** Village territory corresponds to all land, including fields, forests, and savannas, managed by customary law (that is, accepted Tio law governing lands and applied by the village chief). Village A is included in the territory of a larger neighboring village.

*** Forest cover was obtained based on an unsupervised classification (K-Means) on a Landsat 8 image (August 14, 2016) on 64 km^2^ around each village, using ENVI 4.5. Spectral classes were interpreted based on our knowledge of the area and the Normalized Difference Vegetation Index (NDVI) [21].

**** Edge density index index was calculated by dividing the length of the edge by the forest cover area.

**Fig 1:**
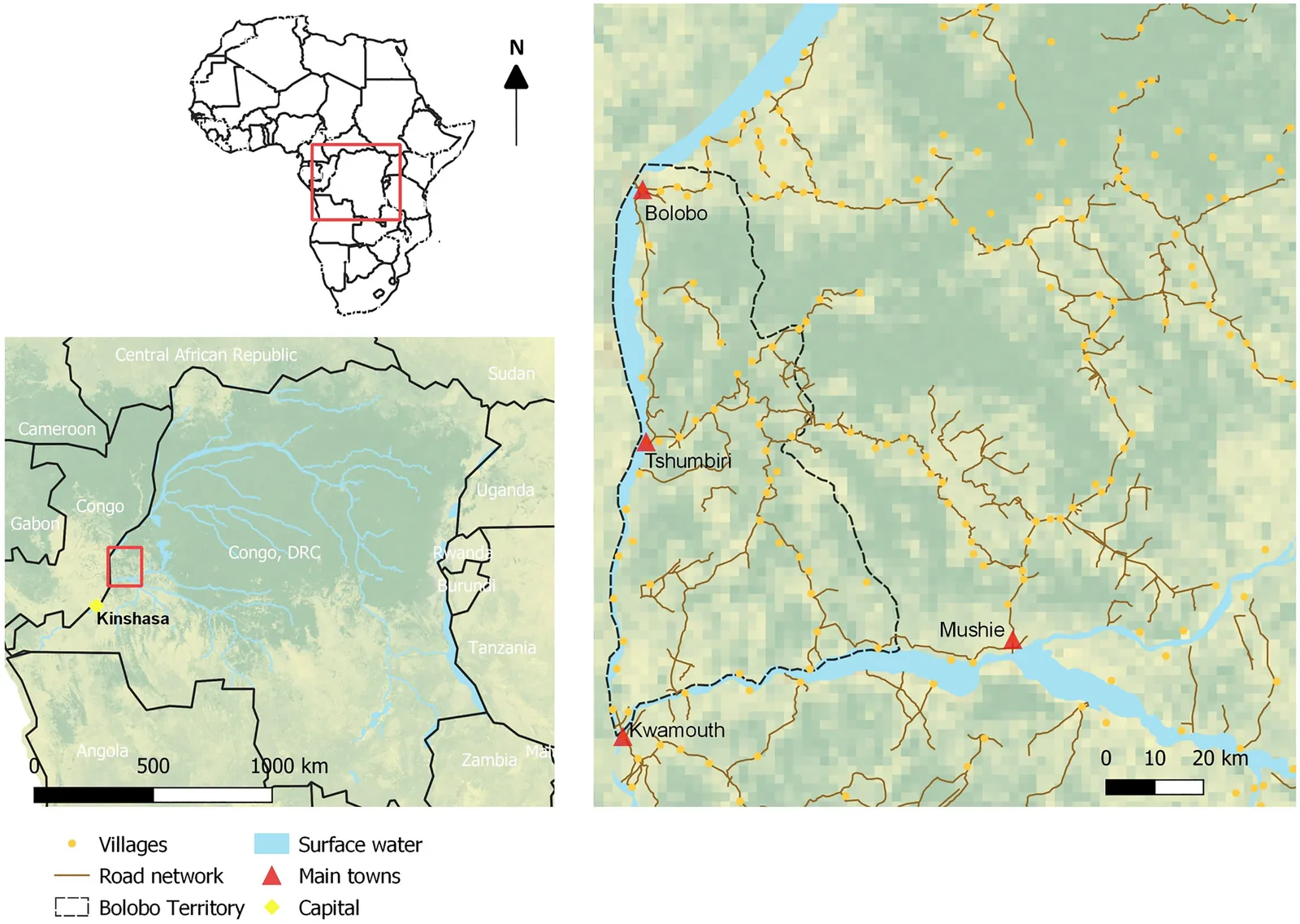
Location of the Bolobo Territory. Base map from Natural Earth. Towns, villages, road networks, and surface water from World Resource Institute. Country borders layer from geoportal (https://geoportal.icpac.net/layers/geonode%3Aafr_g2014_2013_0).

The region’s mean annual pluviometry is 1625 mm, with a rainy season lasting from October to May and a dry season from June to September [24]. Its forests and savannas host highly diverse terrestrial and arboreal mammals such as antelopes, boars, rodents, carnivores, and nonhuman primates (including bonobos, *Pan paniscus*) [25]. The village also houses several domesticated species, notably chickens, goats, sheep, dogs and cats, as well as commensal species such as mice and bats. Few farmers raised cattle in savanna zones outside of the villages [26].

Human population density is low (< 10 ind./km^2^), and human settlements are situated in savanna zones. Although one town on the banks of the Congo River has approximately 10,000 inhabitants, most village populations range between 250 and 3,000 people. The predominant ethnic group is Tio, part of the larger Teke (or Bateke) linguistic group which migrated from what is present-day Republic of Congo to this zone, likely during the 18^th^ century as a consequence of slave and other trades [27]. This population generally practices subsistence swidden agriculture, gathering, hunting, fishing, and engages in local trade. Inhabitants do not hunt and consume bonobos, which they consider “almost human” [28].

For the past 25 years, this study area has also been the site of a community-based conservation project developed by the Congolese nongovernmental organization (NGO) Mbou-Mon-Tour (MMT) [29]. First initiated in 1997 to serve local development needs because of diminishing game and fish populations, MMT is led through local, bottom-up governance [28]. It has supported bonobo conservation since 2001, with six sites (including villages B and C) for eco-tourism and scientific research [29]. MMT reinforces long-standing local taboos of hunting and consuming bonobos and is largely supported by local populations. In 2018, MMT obtained from the provincial ministry of the environment a decree transforming forests for bonobo conservation into community forests (*Concession forestière des communautés locales*) [30], banning hunting, fishing with poison (other techniques allowed), agriculture (small, forest-edge fields tolerated for those living nearby) and timber exploitation. Gathering is permitted. Each of our village sites relied on other nearby forests for unrestricted livelihood activities. In addition to bonobo conservation, MMT conducts regular awareness campaigns for forest conservation. An international conservation NGO (World Wildlife Fund) has also operated in the same area since 2006, focusing primarily on bonobo protection [28].

### Data collection

#### Animal contact and forest activity frequencies through a participatory approach.

To evaluate the diverse types and frequencies of contact between humans and animals in near real-time, we adapted a participatory data collection tool previously developed by research team members to evaluate contacts with non-human primates and simultaneous human activities in the forest [15]. Because this tool documents individual contacts and activities each day, it provides more granular, robust data than that collected through questionnaires, which ask respondents to estimate their contacts over a much longer duration.

We sought to recruit adults in each village belonging to a range of age, gender, and family (kin) groups. We targeted healthy adult participants with regular activities in forest and savanna. We excluded children and teenagers because ethics committees required specific research protocols that would have exceeded our human and financial resources. Because participation was remunerated and thus highly sought after, we requested each village chief in our three sites to select ten participants (hereafter “volunteers”) per village (total 30) to avoid creating local tensions. Village chiefs therefore chose participants from each site to convene to our inclusion criteria (adult >18 years old, healthy, resident of study village, regular practitioner of activities outside of the village). We encouraged chiefs to recruit individuals from different lineages and villages neighborhoods and to respect gender parity. Despite our guidelines, village chiefs (male authorities in a patriarchal society) selected more men than women. Men also generally had higher educational levels and more free time than women and were more able to participate, even when we provided scribes for women who could not read or write. Additionally, more men than women volunteered to participate. Overall, our male participants outnumbered female participants (19 and 11, respectively).

We trained adult volunteers for daily self-recording on paper datasheets to document their contacts with animals and daily forest and savanna activities. After an initial training, a study team member visited all volunteers at regular intervals (~ once a week) to check their data collection validity.

All trained volunteers documented their animal contacts for five consecutive months, filling out two datasheets daily in their local language (Etio or Kiteke). On the first datasheet, volunteers recorded any contact with an animal, either domesticated or not, its vernacular name, and signs of its presence. Among non-domestic animals, we excluded arthropods because such collection necessitated a different protocol (arthropod capture). Because the risk of zoonotic transmission is much higher when two hosts are phylogenetically close, we asked volunteers to document mammalian contacts, not those with undomesticated birds and fish. On the same datasheet, volunteers also documented the type(s) of contact taking place: indirect (participant saw feces, food remains, nest/hole/burrow and/or footprints), direct (participant saw animal alive or dead, or heard animal); or physical contact (participant hunted, transported, butchered, prepared/cooked, ate, purchased, or sold the animal). On a second data sheet, volunteers documented the activities performed that day (hunting, fishing, agriculture, gathering, cattle breeding, or no activity in the forest or savanna) (A in S1 File).

Data quality was ensured through multiple measures. Volunteers underwent training by Romain Duda, who monitored their study activities during the first and last months of data collection, as well as a full-time research assistant (Robert Monfura), who supervised volunteer data collection during the rest of the participatory study. Because several volunteers could not write, we trained a young literate family member to assist them in filling out the data sheets. We also regularly verified information collected, requesting that volunteers orally recount their contacts with animals over the past 48 hours. Of the 30 volunteers recruited, we excluded six because their data were unreliable or inconsistent. The final dataset included 24 volunteers (7 women, 17 men), with a mean age of 48 years old (+/- SD = 18, range = 22–80), reflecting recruitment from wide-ranging age groups. We obtained a mean of 160 days with data on animal contacts per volunteer (+/- SD = 5.3; range = 144–167).

#### Transect survey.

We developed faunal transects for each village site to estimate the relative abundance of certain animal species for which we could obtain signs of presence. Developing an 8*8 km square, with each village at the square’s center, we then drew 2*2 km squares inside of the larger square, so that each resulting diagonal of the sub-squares constituted a transect. We then divided each transect into a 50-meter section. For each section, local trackers from the NGO MMT were trained by Victor Narat to record all animal traces. These trackers all had deep experience recognizing animal signs of presence. Certain transect sections were not possible to access because landowners did not permit data collection. We therefore censused a total distance of 44.45 km, 41.50 km and 44.90 km for villages A, B, and C, respectively. A data collection error in Village B meant that non-human primates were not registered there and were not included in the transect analyses.

#### Household survey for commensal species.

Scientific studies show that certain housing infrastructures and building materials are associated with a higher frequency of synanthropic species (notably mice and bats) [31–33]. We therefore conducted a short questionnaire (B in S1 File) to evaluate the presence or absence of two major commensal species (*Mastomys natalensis* and *Pipistrella* sp.) inside houses, and to document housing types, namely the roofing type (straw or sheet metal), wall materials (brick or mud), and the effects of the construction type on commensal distribution. We opted to address these two species because in our preliminary investigations, village inhabitants reported that they were the principal ones living inside of their homes. Over two days in each village, a research assistant conducted the survey with all household heads available at that time. We conducted surveys among 272 households in total (99 in village A, 136 in village B, and 37 in village C).

#### Semi-structured interviews, participatory and direct observation.

To gain greater insight into current human-animal contacts and the underlying historical dynamics shaping them, we collected ethnohistorical and anthropological data with village inhabitants using semi-structured interview guides, informal interviews, focus group discussions, and participant-observation. Each ethnohistorical, anthropological interview and focus group lasted 30–60 minutes, with discussions focusing on local histories of hunting and wildlife dynamics and behavior, relations with commensal species, goat and sheep raising, poultry farming, dogs as hunters and guards, cattle raising, diet and food avoidances, habitat and landscape dynamics, and illnesses. We also asked participants to recount changing land uses (that is, qualitative descriptions, often with field visits, to describe past practices and uses of land) (C in S1 File for semi-structured interview guides). We employed focus group discussions to validate vernacular species names (using [34]) and to understand local categorizations of animal species and ecological habitats. Participant-observations documented daily activities, including hunting, fishing, and gathering. We conducted 88 semi-structured interviews, 8 focus group discussions (approximately 5 persons/group), and around 30 hours of participant-observation.

For qualitative data collection, we recruited adult participants based on their willingness to participate. Our recruitment relied on snowball sampling; we asked our participants to identify other knowledgeable people who might be willing to participate. We followed local norms of prioritizing certain gender and age groups to find individuals with deep knowledge about specific subjects. Hence, for interviews about domestic animals, we sought out middle-aged and older men to discuss cattle-, goat-, and sheep-herding, and women of all ages for chicken and duck raising. Interviews focusing on forest and savanna species were conducted with active or retired hunters (mostly men). For historical interviews, we sought out elders (mostly men, who are purveyors of such knowledge), born in one of the three villages and coming from different families. We conducted interviews to saturation, that is, until we gathered no new knowledge. We led all interviews and focus group discussions in French, which our research assistant translated directly into Etio. We recorded these exchanges if the participant consented, and for the few who refused, we took detailed notes. All recordings were transcribed and then translated into French by a native Etio speaker.

### Data analysis

#### Animal contact frequencies and clustering analysis.

We developed our animal contact frequency and clustering analysis to provide broad insight into human exposures to animals and evaluate risks of pathogenic exposure without imposing pre-conceived analytical categories. Although zoonotic spillovers can occur through rare contact with specific mammals, contact frequency was important to evaluate because increased numbers of contacts over time increased exposure and in turn the possibility of spillover [35,36]. We therefore developed an index of presence/absence per day to quantify the frequency of one type of contact for one animal species. We defined a “contact event” as one volunteer having one type of contact with one animal species on one date. Thus, if on a specific day a volunteer had three types of contact with one species, we would count these engagements as three distinct contact events. We calculated for each volunteer a frequency for each possible interaction “animal species*type of contact” (thus, for each animal species and each type of contact) with the following formula:


$$\begin{array}{c} F\text{v},\text{c},\text{s}=\frac{N\text{v},\text{c},\text{s}}{T\text{v}} \end{array}$$

where Fv,c,s is the frequency of the type of contact *c* with the animal species *s* for the volunteer *v*, Nv,c,s is number of days with a recording of the type of contact *c* with the animal species *s* by the volunteer *v*, and Tv is the total number of days with data for the volunteer *v*.

The same method was used to calculate the frequency of each human activity, including different techniques used for hunting and fishing. We used the following formula:


$$\begin{array}{c} F\text{v},\text{a}=\frac{N\text{v},\text{a}}{T\text{v}} \end{array}$$

in which Fv,a is the frequency of the activity *a* for the volunteer *v*, Nv,a is number of days with a recording of the activity *a* by the volunteer *v*, and Tv is the total number of days with data for the volunteer *v*. Thus, for each volunteer, we obtained a frequency for each category of activity (hunting, fishing, agriculture, gathering, cattle raising) and a frequency for each specific technique of hunting and fishing.

To avoid imposing our own assumptions about animal species categories (e.g., “domestic” or “wild”), we used a cluster analysis to identify animal categories of contact profiles based on the contact frequency for each contact type. Our goal was to cluster by *contact profile*, not by species biology, because we wanted to understand human exposure patterns. Predefined categories of “domestic” and “wild” obscure diverse animal ecologies and behaviors and may simultaneously impose assumptions about differences across a presumed “domestic-wild” divide. “Wild” animals on the forest edge are highly diverse in their phylogenetics, ecologies, and behaviors, and thus may bear little resemblance to one another, thus reducing the relevance of the category “wild” in assessments of contact and potential pathogenic exposures. Simultaneously, presumed differences between certain “wild” and “domesticated” mammals may be less appropriate: free-range goats, for instance, may forage in sites where small antelope also forage.

We performed a Principal Component Analysis (PCA) to search for correlations between contact types and to reduce the number of dimensions. For each animal species, we calculated the mean contact frequency (based on the 24 volunteers included) for each type of contact; each animal species was associated with 14 values (for the 14 types of contact studied). We performed the PCA on these for mean contact frequencies, considering each animal species as a statistical individual and each contact type as variables for comparison. We then reassigned the coordinates of the first two axes to each animal species and performed on these reassigned coordinates a hierarchical cluster analysis (HCA, R package Cluster [37]) with the Ward method [38]. To characterize each cluster obtained, we compared mean frequencies for the initial 14 contact types with the mean relative abundance between the three clusters, using a Kruskal Wallis test and a pairwise Wilcoxon test with Bonferroni correction.

#### Influence of relative abundance index, gender and village on animal contact frequencies.

Because we examined human-animal contact from human perspectives, we included one variable to account for the abundance of each animal species and its proximity to the village. We hypothesized that for any given animal species, the higher abundance and closer proximity to a village (and thus to human activities) would increase the probability of human-animal contact. For this reason, we calculated a relative abundance index for each animal species, adapted from Narat et al. 2018 [15], with the following formula:


$$\begin{array}{c} RAs=\sum (\frac{\text{1}}{Di,s}) \end{array}$$

where RAs is the relative abundance index for the species s, and Di,s is the Distance (km) of the sign of presence i for the species s recorded during transect survey.

We then built Generalized Linear Mixed Models (GLMMs) to test the influence of village, gender, relative abundance index, and frequencies of forest activities on contact frequency. We built one model for each interaction between the contact category obtained from the PCA and the clusters of animal species from the HCA. The response variable was the contact frequency, and the predictive variables (fixed effect) were village, gender, human activity frequency, and relative abundance of animal species. We controlled for volunteers and animal species as random effects. For each model, we compared the full model vs the null model: we assessed whether a full model with fixed effects plus random effects yielded better results than a null model with only random effects. If the former could not explain more variance than the latter, it indicated that the predictive variables did not explain much of the variance of contact frequency. The details of the models and their interpretative plots are provided in the supplementary materials (S2 File).

#### Commensal species and house type.

We analyzed the presence of mice and bats in house and relations with housing materials. We conducted the statistical analysis by employing Fisher’s exact test for each variable individually, followed by a combination of variable tests.

#### Ethnohistorical and anthropological data on human-animal interactions and historical processes.

Ethnohistorical and anthropological data necessitated analytical synthesis that accounted for diverse (generational, gender, livelihood) perspectives of our participants. To that end, we imported transcripts and observation notes into qualitative data analysis software to code and label inductively text segments. We inductively coded by reading transcripts and notes closely and highlighting recurrent phrases related to human-animal interactions, developed codes to describe these texts (such as avoidance of animals; interactions with rodents; interactions inside house), and then refined these codes. Aggregating our codes, we identified themes that characterized the full qualitative dataset, such as contact through hunting; sharing norms; livestock farming; declining undomesticated animals. To enrich our interpretations and develop comprehensive insights, we triangulated results from our quantitative and qualitative approaches. We therefore present results by integrating our insights from both data types.

#### Software used for analysis.

All statistical analyses were performed with R version 4.0.3 [39]. We used ade4, cluster and lmerTest packages to perform the PCA, HCA and GLMMs [37,40,41]. Spatial analyses determining the distance from village site of each sign of animal presence were performed with QGIS Madeira (version 3.4.3) [42]. We conducted our qualitative coding with NVivo 11 software (*Lumivero*, version 1.7.1 1534) [43].

## Results

Below we describe participants, current activities, and the diversity of animal species contacted. We then present historical changes in hunting and land use, the clustering animals by contact profile, and the results of models testing the influence of gender, village, animal relative abundance, and human activity on contact frequency. We conclude with ethnographic analysis of each cluster, which accounts for patterns that the models leave unexplained.

### Participant profiles

The profiles of participants in our data collection activities are detailed in Table 2. Gender and age imbalances reflect specific data collection needs (e.g., older men had deep historical knowledge of hunting) and challenges of recruiting women to participate in the contact investigation.

**Table 2:** Participant profiles in data collection tools.

Data collection tool	Gender		Age	Total
	F	M		
**Volunteer contact investigation**	7	17	18-30: 4 31-45: 8 46-59: 4 60 + : 8	24
**Interviews**	25	63	18-30: 7 31-45: 32 46-59: 14 60 + : 35	88

### Diversity of animal species, human activities and changes over time

#### Current human activities in forest and savanna environments.

Our quantitative data yielded no differences in activity frequencies between the three villages, except for net fishing between villages B and C (Table 3).

**Table 3:** Mean frequencies of human activities by gender and village.

	Gender		Villages			
	Women (n = 7)	Men (n = 17)	A (n = 8)	B (n = 7)	C (n = 9)	Total (n = 24)
Agriculture	0.68 (0.06)**	0.47 (0.16)**	0.56 (0.16)^a^	0.57 (0.16)^a^	0.49 (0.18)^a^	0.53 (0.17)
Gathering	0.65 (0.21)*	0.47 (0.16)*	0.49 (0.17)^a^	0.48 (0.21)^a^	0.59 (0.20)^a^	0.52 (0.19)
Fishing – all technics	0.12 (0.09)	0.09 (0.11)	0.09 (0.09)^a^	0.04 (0.05)^a^	0.15 (0.12)^a^	0.10 (0.10)
Fishing – dam	0.11 (0.08)**	0.004 (0.008)**	0.05 (0.09)^a^	0.02 (0.04)^a^	0.04 (0.06)^a^	0.04 (0.07)
Fishing – hook	0.0 (0.0)*	0.04 (0.06)*	0.02 (0.05)^a^	0.01 (0.03)^a^	0.05 (0.07)^a^	0.03 (0.06)
Fishing – net	0.0 (0.0)*	0.04 (0.07)*	0.02 (0.04)^a^	**0.0 (0.0)** ^**a,b**^	**0.06 (0.08)** ^**a,c**^	0.03 (0.06)
Hunting – all techniques	0.21 (0.17)	0.33 (0.16)	0.33 (0.18)^a^	0.31 (0.15)^a^	0.25 (0.17)^a^	0.30 (0.17)
Hunting – shotgun	0.0 (0.0)*	0.05 (0.08)*	0.03 (0.07)^a^	0.02 (0.04)^a^	0.05 (0.08)^a^	0.03 (0.07)
Hunting – snares	0.21 (0.17)	0.27 (0.14)	0.30 (0.19)^a^	0.26 (0.13)^a^	0.20 (0.13)^a^	0.25 (0.15)
Hunting – net	0.0 (0.0)	0.02 (0.03)	0.01 (0.03)^a^	0.02 (0.05)^a^	0.01 (0.02)^a^	0.01 (0.03)
Hunting – machete	0.001 (0.002)	0.002 (0.004)	0.001 (0.002)^a^	0.002 (0.003)^a^	0.003 (0.004)^a^	0.001 (0.003)
Hunting – pick up	0.002 (0.005)	0.004 (0.005)	0.003 (0.005)^a^	0.003 (0.005)^a^	0.005 (0.006)^a^	0.004 (0.005)
Cattle Breeding	0.0 (0.0)	0.007 (0.02)	0.0 (0.0)^a^	0.01 (0.03)^a^	0.005 (0.01)^a^	0.005 (0.02)
No forest or savanna activity	0.20 (0.11)	0.26 (0.11)	0.28 (0.13)^a^	0.21 (0.07)^a^	0.23 (0.13)^a^	0.24 (0.11)

Standard Deviation is indicated in parentheses. We tested comparisons with Wilcoxon tests for gender and Kruskal-Wallis test and if significant, followed by a pairwise Wilcoxon test with Bonferroni correction, for villages. Letters a, b, c designate results of the pairwise comparisons for villages. When p < 0.05 for two villages, the letters are different between these two villages. *p < 0.05, ** p < 0.01. Significant differences are in bold.

Most activities were conducted in the forest. Inhabitants – most frequently women – practiced swidden (slash-and-burn) agriculture, producing cassava (for subsistence and local trade), maize (for trade), plantain (subsistence and trade), and some fruits, legumes, and vegetables, such as pineapple and groundnuts (Table 3). Inhabitants gathered edible plants (e.g., *Gnetum africanum*)*,* mushrooms, caterpillars, medicinal plants, and building materials such as lianas for baskets or *Marantaceae* leaves for wrapping food. Women gathered more frequently than men (Table 3).

Hunting was the third most performed activity, conducted with multiple techniques which targeted diverse animal species and entailed different exposures to biological fluids. The first, shotgun hunting, was practiced exclusively by men (Table 3). This technique targeted arboreal monkeys, duikers, antelopes, carnivores, and sometimes large rodents or birds. As one hunter acknowledged, “When you hunt with shotgun […] you target everything that crosses your path, even birds.” (140921-B)

Other techniques included snare trapping, net hunting, machete hunting and live collection. Trappers, usually men, set up their snares in the forest and at the edges of their fields to capture medium-sized animals, such as large rodents, duikers, snakes, or carnivores (020419-C, 050819-A, 090921-B). Women often checked the traps set by their husbands when they visit their fields, collecting any captured animals. Net hunting, an exclusively male activity involving groups of 15–20 men of different ages, was performed only in the forest (150921-B, 160921-B). Both women and men engaged in machete hunting, primarily for slow animals like pangolins and turtles (150921-B) and used fire to extract Emin’s pouched rats from their burrows (030419-C).

Both women and men also practiced fishing, although they employed different techniques in forest and savanna streams (091021-C, 150921-B). Women performed more dam fishing (building a barrier in a running stream and emptying the water to capture fish) than men (Table 3), whereas men engaged in line and net fishing more than women (Table 3).

Two male volunteers out of 24 raised cattle, typically keeping 2–5 heads on their savanna ranches. Although few engaged in this activity (itself a smaller scale adaptation of the 1950s-era colonial cattle-based economic model), cattle-breeding, according to our participants, was reshaping habitats and settlement patterns, leading to the privatization of once-customary, communal savannas, and restricting historical practices of mobility, hunting and cultivation. Herding, our participants contended, has increased forest exploitation and has generated social and human-animal conflicts (040419-A, 020419-A, 260319-C, 040419-A, 120921-B).

#### Contacts with a large diversity of animal species.

Our volunteers were in contact at least once with 61 different animal species, including nine domesticated species. These 61 animals were recorded in the following Orders: Artiodactyla (n = 14 species), Carnivora (n = 14), Rodentia (n = 12), Primate (n = 6), Chiroptera (n = 3), Squamata (n = 2), Afrosoricida (1), Anseriforme (n = 1), Crocodilia (n = 1), Columbiformes (n = 1), Galliformes (n = 1), Hyracoidea (n = 1), Macroscelidea (n = 1), Pholidota (n = 1), Testudine (n = 1), Tubulidentata (n = 1). Over the five-month volunteer study, 97,917 contact events were recorded, with a mean of 4,080 contact events/volunteers (+/- SD = 1,084; range = 2299–6234). The volunteers reported contact with 7.5 different species per day on average (+/- SD = 1.6, range = 5.0-10.1), including 4.8 domestic species per day (+/- SD = 1.1). We found no statistical difference between men’s and women’s contact events or species diversity per day, but volunteers from Village A had a higher number of contact events and diverse animal species/day because of their higher contact frequency with domesticated species (Table 4).

**Table 4:** Mean number of contact events (Standard Deviation) - all contact types - and mean number of animal species (Standard Deviation) for all volunteers, by gender and village.

	Gender		Villages			
	Women (n = 7)	Men (n = 17)	A (n = 8)	B (n = 7)	C (n = 9)	Total (n = 24)
Contact events per volunteer over the study period (SD)	3,979 (960)	4,121 (1,156)	**4,737 (403)** ^**a**^	4,179 (1,192)^a, b^	**3,419 (1,113)** ^**b**^	4,080 (1,084)
Animal species (SD)	33 (7.3)	35 (5.0)	31 (4.5)^a^	37 (6.5)^a^	35 (4.4)^a^	34 (5.6)
Domestic animal species (SD)	9 (0.4)	9 (0.6)	9 (0.5)^a^	9 (0.8)^a^	9 (0.3)^a^	9 (0.5)
Animal species/day (SD)	7.2 (1.8)	7.6 (1.5)	**8.6 (0.9)** ^**a**^	7.4 (1.8)^a,b^	**6.5 (1.2)** ^**b**^	7.5 (1.6)
Domestic animal species/day (SD)	4.5 (1.2)	4.9 (1.1)	5.8 (0.3)^a^	4.6 (1.1)^a, b^	4.1 (0.9)^b^	4.8 (1.1)

Standard Deviation is indicated in brackets. We tested comparisons with Wilcoxon tests (Bonferroni correction for villages). Letters a, b, c designate the results of pairwise comparisons for villages. When p < 0.0.5 for two villages the letters are different between these two villages. Significant differences are in bold. Results of statistical tests are detailed in S1 Table.

#### Historical changes in hunting and wildlife profiles.

Our oral historical interviews helped to shed light on the longer-term processes that shaped these contact events with diverse animal species. These historical accounts revealed that past hunting practices had cumulative effects on current undomesticated fauna populations, and thus on human-animal contact.

Many elders recounted undomesticated faunal diversity has experienced important changes over the past several decades, and that prior generations had more interactions with a higher diversity of animal species. As one elder man put it,

“Past generations hunted more than today, with nets, guns, traps. [In previous times] the elders went 3 or 4 days into the forest to hunt with nets or traps. There were big hunting expeditions (*molao*) in the dry season. The last time we saw this here was in the 1990s” (090921-C).

Our participants frequently characterized their current relations with forest and savanna animals (*nyama a mofuru* and *nyama a ntsio*, respectively) in terms of the past. They complained about declining numbers of undomesticated fauna over their lifetime, as evidenced by their current difficulties encountering and hunting specific animals (020419-C; 300319-B). Former hunters regaled us with past stories of returning from the hunt with 10 large rodents or duikers, an offtake that would be considered impressive today. Most elder male participants explained that current wildlife population depletion resulted from high hunting pressures during their youth or their parents’ lives (010419-B; 020419-C). Before the 1960s, they explained, undomesticated animals ranged close to villages, making hunting relatively easy, and selling meat earned quick money (260319-B; 120921-E). Several monkey species (*Pilicolobus sp.*, *nkana* in Tio; *Angola colobus, mvu; Lophocebus sp., nzela*) were thus decimated in the 1960s and 1970s. Moreover, from the 1970s, bongos (*Tragelaphus eurycerus, ngonoju*), panthers (*Panthera pardus, ngo*), black-fronted duikers (*Cephalophus nigrifrons, bokeli*) and giant pangolins (*Smutsia gigantea, nkau*) nearly disappeared, accelerated by the democratization of 12-gauge shotguns. As another retired hunter regretfully observed,

“We have made bad use of the forest. Before, a hunter could kill 20 monkeys in one go. […] After 1972 there were a lot of hunters, a lot of shotguns, a lot of 12-gauges” (120921-B).

It was in response to this cumulative decline in undomesticated mammal populations that MMT was founded to develop hunting alternatives and eventually, to conserve bonobo populations. More recently, the MMT bonobo conservation project altered access to certain forest areas, where hunting is forbidden. This restriction, too, can affect not only inhabitant contacts with bonobos but also other forest mammals.

#### Changes in land use and perceived consequences on faunal structure.

Hunting was just one of multiple livelihood practices in these study sites and thus through oral historical interviews and field visits with participants to address land use, tenure, vegetation, and faunal changes, we investigated how past land use dynamics shaped participant engagements with animals.

Many oral historical interviews revealed that since the 1970s, several land tenures changes have affected ecological habitats and faunal structures. Just before DRC independence in 1960, cattle farming gradually expanded into savanna lands, which until then had been used for agricultural production and hunting. Expanding cattle raising thus appropriated these lands, displacing hunting and farming activities into forest lands. Thereafter hunters conducted most activities in the forest, reducing their hunting of savanna-dwelling animals. In addition, the displacement of agriculture into forested areas increased pressures on available lands, reducing fallows from 5-10 years to 2–3 years. Not only did decreased fallows lead to decreased productivity of agricultural lands, but they also produced what some informants called “dead forest”, that is, the colonization of fallowed lands by the invasive *Chromolaena odorata* (*felele*) and ferns, primarily *Pteridium spp.* (*enye*). As focus group participants explained,

- “After we work the fields for several years, ferns (*enye*) emerge.”

- “Yes, first we have the forest (*mokuna*), which is transformed into the fallow (*evuu*), which brings the “flower” (*felele*), after *felele* comes the ferns: and there it’s the end!” (140921-B, focus group).

Our participants explained that fallowed lands converted more easily into savanna, thus affecting animal diversity and abundance. They suggested that fern colonization of these sites could create habitats favorable to large rodents -- cane rats (*Thryonomys swinderianus, nsili*), Emin’s pouched rats (*Cricetomys emini, nkuli*), brush-tailed porcupines (*Atherurus africanus, nkeon*), and small carnivores (140921a-B).

### Types of contacts and categorization of animal species

#### Categorization of animal species according to their contact profile.

We categorized animal species according to their contact profile. Mean frequencies for contact with each species and each type of contact are detailed in S2 Table. The first two axes of the PCA explained 81% of the variance. Visualization of the 14 types of contact within the two first axes shows high correlation between two broad contact types (Fig 2): all physical contacts plus “seen dead”, and all environmental (direct and indirect, except for “seen dead”) contacts. We therefore consolidated these diverse contacts into two categories, physical and environmental. We reassigned the first two coordinates of the PCA to each animal species and performed HCAs. We obtained three clusters of animal species according to their contact profiles (Fig 3 and S3 Table). The pairwise comparisons of these clusters indicated significant differences (Fig 4 and S4 and S5 Table). The first cluster (n = 46 species) represented most animal species and was characterized by low contact frequency for all types of contact. The second cluster (n = 6) was characterized by animals with which humans had the highest physical contact rates; the third cluster (n = 9) had the highest frequency of environmental contacts. This latter cluster is composed of all domesticated species (except rarely-raised pigs, *Sus scrofa domesticus*, *ngulu*) and a commensal rodent species (*Mastomys natalensis*, *mpu*).

**Fig 2:**
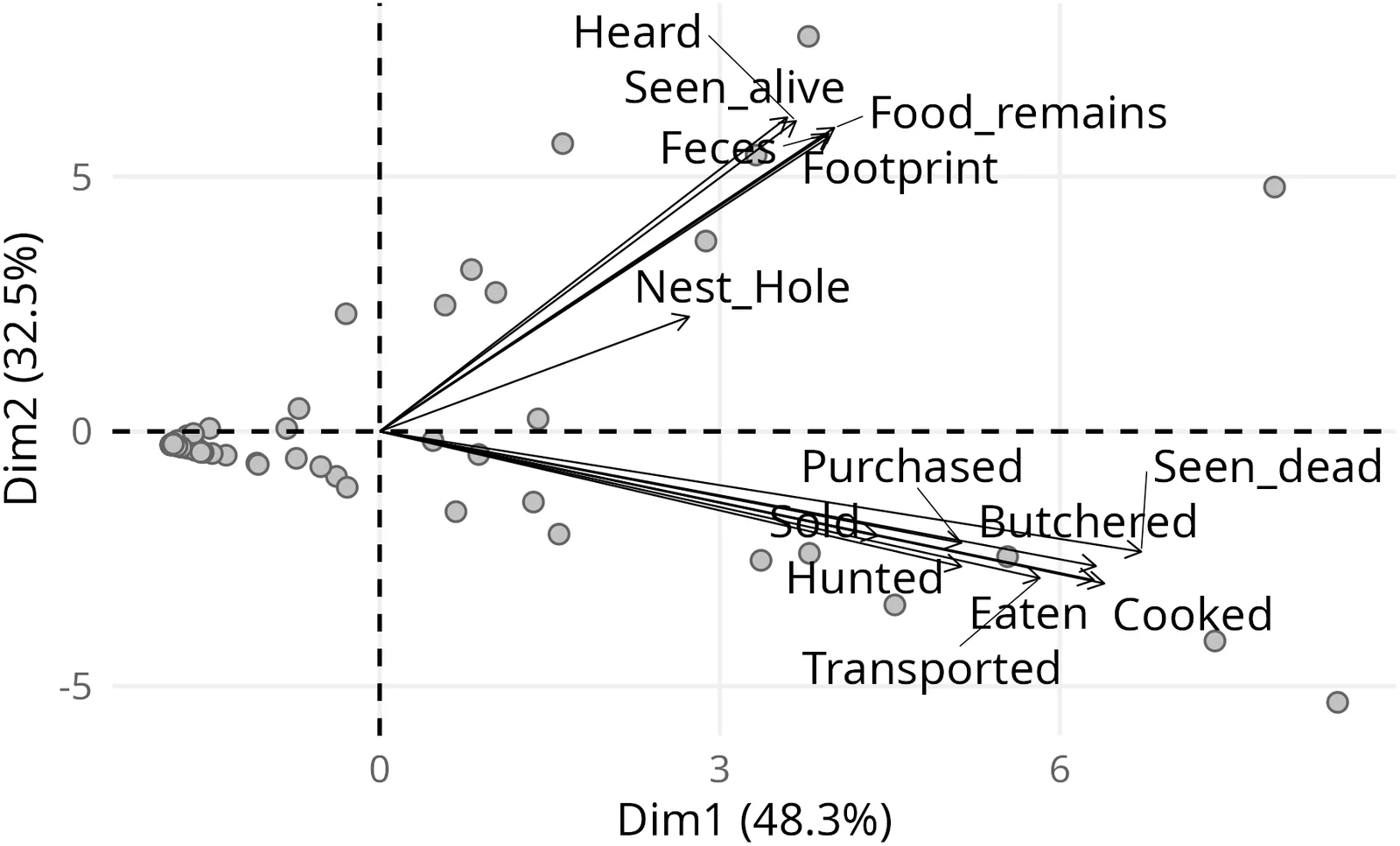
Principal Component Analysis of animal species according to frequency of contacts with humans for each type of contact. The two first axes explained 81% of variance.

**Fig 3:**
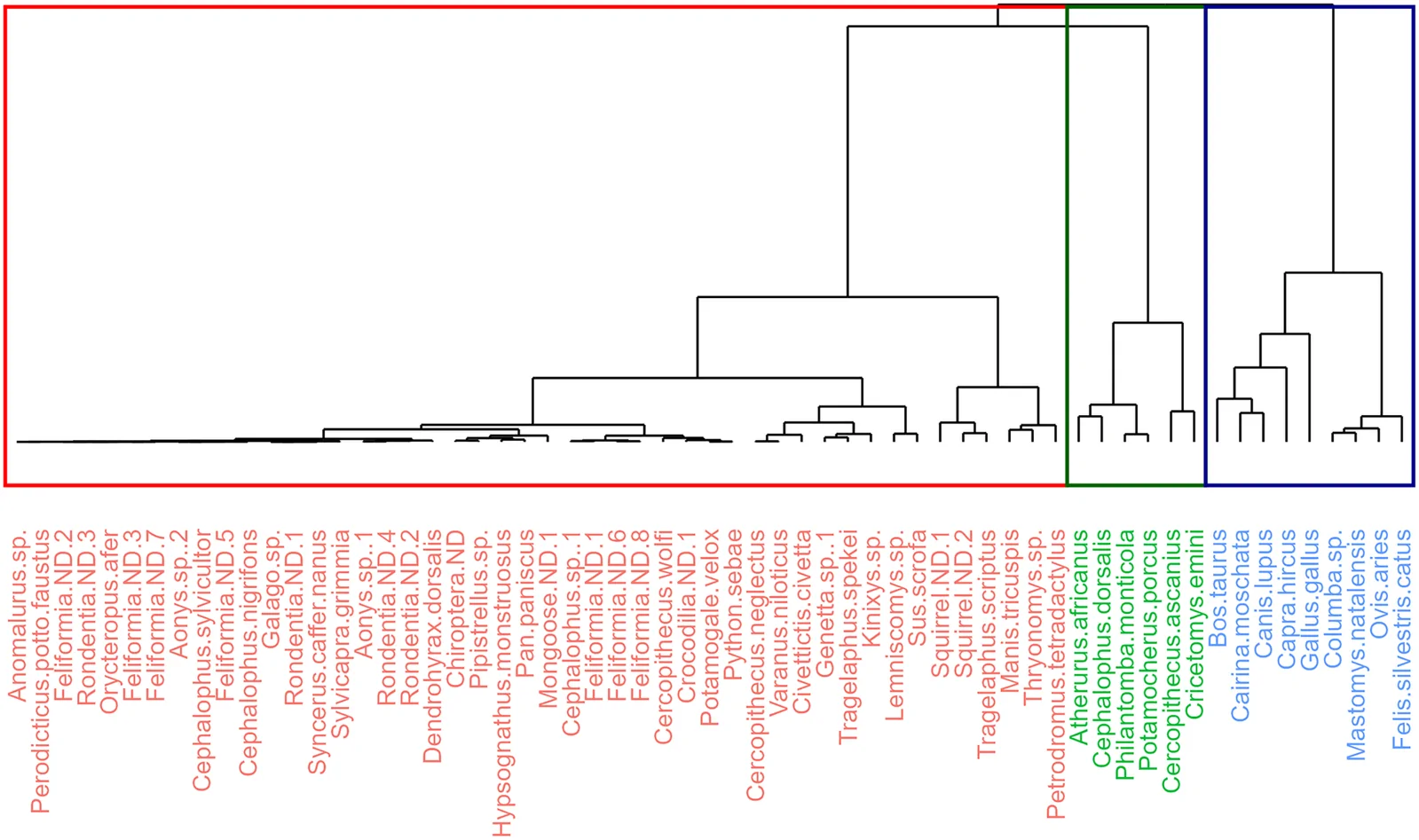
Hierarchical cluster analysis of animal species according to projected coordinates of the two first axes of the PCA.

**Fig 4:**
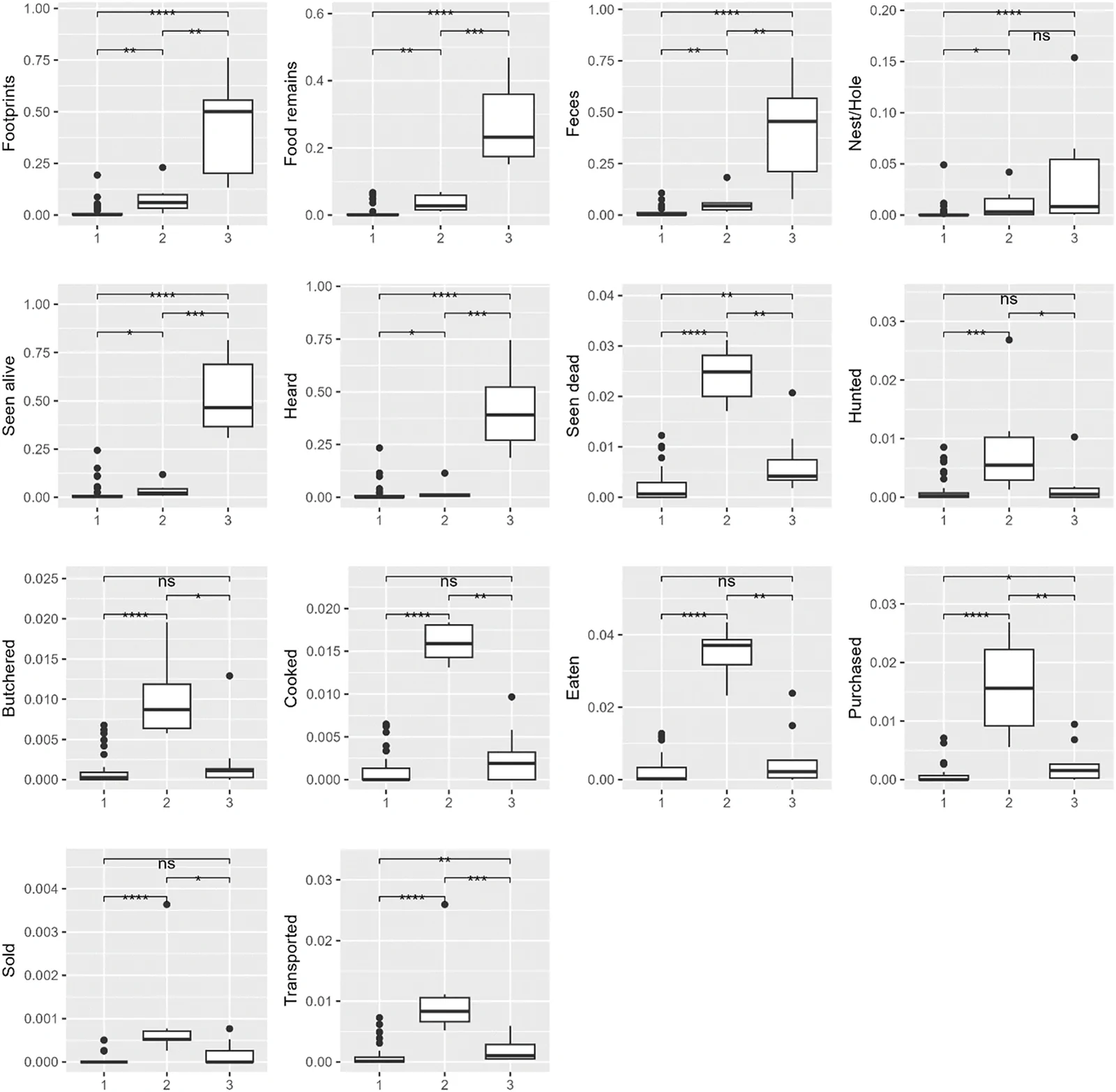
Boxplot comparisons of contact frequencies for each type of contact among the three clusters obtained with the HCA. For each boxplot, the black bar represents the median; white boxes display the interquartile range (25-75th percentile). The lower limits of the vertical lines represent the smallest value within 1.5 times the interquartile range below the 25th percentile, and the upper limits represent the largest value within 1.5 times the interquartile range above the 75th percentile. Black dots are values outside of these limits. ns = not significant; * p < 0.05; ** p < 0.01; ***p < 0.001; ****p < 0.0001.

#### Influence of village, gender, species relative abundance, and human activities on contact frequencies.

To test the influence of village, gender, species abundance, and human environmental practices on contact frequency with animals, we performed six GLMMs, one for each interaction between the contact category from the PCA (physical or environmental contact) and the animal species cluster from the HCA (cluster 1 with low contact frequency; cluster 2 with high physical contact frequency; and cluster 3 with high environmental contact frequency). We obtained relative abundance values for 32 of the 61 animal species recorded in the contact dataset and thus performed these GLMMs on these 32 animal species. Table 5 synthesizes the results (see S2 File for detailed result).

**Table 5:** Synthetic results of GLMMs.

	Cluster 1		Cluster 2		Cluster3	
	Environmental contacts	Physical contacts	Environmental contacts	Physical contacts	Environmental contacts	Physical contacts
Village	Village B weak positive	Village A, weak negative	/		Village A, positive, Village B tendency, positive	/
Gender			/			/
Relative abundance	weak positive	weak positive	/	weak positive	weak positive	/
Hunting – all techniques			/	weak positive		/
Fishing – all techniques		tendency, weak positive	/	tendency, weak positive		/
Gathering			/			/
Agriculture			/	negative		/
No forest activity	negative		/			/

When full model was significantly different from the null model, we report the potential effect of each variable. A blank cell means no significant effect. We used “/” to indicate that the full model was not significant compared to the null model.

Within the six GLMMs performed, four models were significant in comparison with the null model. Among these four models, gender had no influence on contact frequency. Village site influenced contact frequency but displayed no clear pattern. Relative abundance had a significant and positive association with contact frequency (weak effect) for all the four models. Hence, higher relative abundance was associated with higher environmental and physical contact for cluster 1 species, higher physical contact for cluster 2 species, and higher environmental contact for cluster 3 species. Cluster 2 species had higher contact frequency with humans heavily engaged in hunting and fishing, and lower contact frequency with those more heavily engaged in agricultural activities. Thus, the models’ parameters explained only a small part of contact frequencies with animals.

Our ethnographic, ethnohistorical analyses, however, offered potential insight into the processes shaping human-animal contact clusters.

#### A web of environmental contacts for cluster 3 animals.

Cluster 3 animals, largely domesticated species, lived near or with human inhabitants of the three village sites. We observed domestic animals roaming freely in these villages, sometimes ranging as far as the forest or savanna edges abutting on these villages. These domestic animals had different ranging behaviors and were treated differently by their owners.

Chickens and ducks remained close to households to which they belonged, sometimes entering homes in search of stored maize. At sundown, to protect flocks from theft or mongoose attacks, women owners coaxed their poultry into enclosures next to their houses or into separate kitchen structures. Our participants explained that poultry required little time investment, management, or physical contact. Current flock sizes, ranging from 2 to 10 birds, were much smaller than those of 20 or 30 years ago because of recurrent epidemics and owners’ more limited purchasing capacities (010821-A).

Goats, and to a lesser extent sheep, were also free ranging. Goat and sheep herds tended to mingle with those of different owners during the daytime. They also crossed paths with village poultry. At times, they entered open houses in search of drying cassava. Human contact with goat and sheep feces occurred frequently; children regularly cleaned this excrement and discarded it in open waste areas next to household concessions.

Pigs were rarely raised, and thus environmental contact occurred rarely. As several participants explained, village inhabitants ‘“evicted” them because they host parasites and damage property’ (060819-A; 091721-B).

Our interviewed participants frequently used to term *ngeyi* to characterize certain domesticated animals (*nyama a bola,* litt. *“*animal of the village”, e.g., ducks, sheep, goats). *Ngeyi* translates as “disgusting” or “repulsive”, emerging from local perceptions that an animal is “dirty” or “impure”. For cluster 3 animals, our participants evoked *ngeyi* because of their tendency to roam and feed in sites where human waste (feces, urine) was found. Older participants more frequently described cluster 3 animals as *ngeyi* than younger participants. Perceiving an animal as *ngeyi* led to avoidances, notably of touching the live animal or consuming its meat.

Additionally, domesticated animals generally functioned as a “living bank accounts” and were thus rarely eaten or touched while alive. An owner could slaughter a goat to obtain urgently needed cash (for health expenses, funerals), selling its small meat portions locally. Inhabitants sold their sheep to Muslim merchants from neighboring Republic of Congo, notably in Village A, closest to the market town and the Congo river (040819-A). Cattle were frequently sold and exported for revenue except when slaughtered for local festivities.

Engagement with dogs differed from that with other domesticated animals. Village inhabitants used dogs for solitary spear or collective net hunting and for guarding their homes. During the day, dogs roamed throughout the village searching for other dogs and leftovers in waste areas (the same sites where animal wastes were discarded and young children defecated) (210921-A, 110921-C). Dogs were rarely in physical contact with humans, except for their owners. The latter engaged in physical contact with dogs primarily when hunting and when managing, caring for, and feeding them. Dogs also maintained close environmental contact with their owners, sleeping in the main house or kitchen area.

Very few cats were kept in villages A, B, or C. The few cats we observed were acquired solely to rid homes of mice; they moved between different houses but were not cared for, handled, or fed.

The sole undomesticated species in cluster 3 was the mouse *Mastomys natalensis*. Our three-village survey with all households found that all respondents except three had mice in their houses, but that housing (roof and wall) materials had no effect on mouse presence. Our volunteers also reported important environmental contact frequencies with *M. natalensis* (notably, “seen alive” mean = 0.31, SD = 0.20, min = 0, max = 0.68). Village inhabitants in all sites considered household mice to be a pest *par excellence*, partly because of their destructiveness, but also because of their perceived impurity, in contrast to forest mice and rats. Survey and interview results showed that people did not eat house mice, although forest and savannah mice provided frequent supplemental meals for children (090821-C). Village inhabitants sought to control *M. natalensis* with human anti-inflammatory drugs, rat poison, salt, rarely cats, or by bludgeoning them with sticks or branches (150921-B).

Our participants actively avoided touching living or dead house mice or their feces. They would pick up a mouse carcass using a piece of plastic or cloth and then discard it in a refuse dump at their concession edge. As two participants noted,

“The mouse is considered the enemy of humans, this is why no one accepts it in their house” (140921b-B)

“It’s an animal of bad faith. It does not come to eat, but to destroy” (080921a-C).

This avoidance of physical contact resulted, participants contended, because mice frequented latrines and could transmit illness. These illnesses went unspecified, although infrequently participants referred to typhoid or “fever” (150921-B). Some used mosquito nets around their beds to avoid contact with mice or their feces. They claimed that mice were wily, blowing or nibbling on the feet of sleeping humans to divert attentions away from other mice destroying human possessions.

#### High physical contact with cluster 2 animals.

Our participants had the highest physical contact frequencies with cluster 2 animal species, which included multiple forest animals: two rodent species (*Atherurus africanus* and *Cricetomys emini*); three Artiodactyle (even-toed) species (*Philantomba monticola, Cephalophus dorsalis, Potamocherus porcus*) and one primate species (*Cercopithecus ascanius*). Our interviewees reported that these species were commonly hunted and killed. Human exposure to a hunted animal’s bodily fluids depended on the hunting technique used, game size and specific sharing rules (Table 6).

**Table 6:** Types of contacts according to species of the cluster 2 (high physical contacts). *[M]: men; [F]: women.

Species with high physical contact	Main situation of contact during capture [by whom]*	Contact through slaughtering and transport	Butchering	Meat sharing and sales (with implications for the scale of physical contact) [by whom]
**Brush-tailed porcupine** *(Atherurus africanus*) **Emin’s pouched rat** (*Cricetomys emini*)	Snare trapping [M,F]	Killed by snare or slaughtered by machete, and transported whole to village [M,F]	Butchered in village [M]	Limited sharing (household level)
	Fire or flushing out, with or without dogs [M]	Killed by human or dog, transported whole to village [M]		
**Blue duiker** (*Philantomba monticola*)	Collective net hunting with dogs [M]	Killed by dog or human (piece of wood or machete), handled fresh [M]	Butchered at hunting site [M], with fresh butchered pieces transported to village [M]	Extensive sharing (hunting team) on the hunting site
	Snare trapping [M,F]	Killed by snare or slaughtered by machete, and transported whole to village [M,F]	Butchered in village [M]	Limited sharing (household/kin group level) or sold in pieces
	Shotgun [M]	Killed and transported whole to village [M]	Butchered in village [M]	Limited sharing (household/kin group level) or sold in pieces
**Bay duiker** (*Cephalophus dorsalis*)	Shotgun [M]	Killed, with quarters transported to village [M]	Butchered in village [M]	Extensive sharing or selling (kin group and village level)
	Collective net hunting with dogs [M]	Killed by dog or human (machete), handled fresh [M]	Butchered at hunting site [M], with freshly butchered pieces transported to village [M]	Extensive sharing (hunting team) on the hunting site
**Red river hog** (*Potamocherus porcus*)	Shotgun [M]	Killed, with freshly butchered pieces transported to village [M]	Butchered at hunting site [M]	Extensive sharing and selling (kin group and village level)
**Red-tailed monkey** (*Cercopithecus ascanius*)	Shotgun [M]	Killed and transported whole (fresh) to village [M]	Butchered in village [M]	Limited sharing (household level) or sold in pieces

Generally, hunters avoided drawing blood during the hunt. If a hunting dog did not kill the game directly during net hunting, the hunter would break the neck of small animals (e.g., blue duiker) and kill larger game with a heavy blow to the animal’s head to prevent blood spillage (150921-B; field observations).

Our participant-observations showed complex meat sharing practices from trapping and individual or collective hunting, and thus uneven exposures to game meat and bodily fluids. The gift of wild meat, reinforcing social cohesion with kin and friends, followed a long-standing patterns with specific butchering rules and “rights” that defined what animal parts or organs a hunter owed to specific recipients; sharing obligations for small animals were less strict than for larger ones (310319-B, 140421-C; see Table 6).

More precisely, two distinct logics governed these obligations. First, we noted a kinship-debt logic in which the hunter allocated specific organs to designated kin group members. Second, we found a participation-based logic, in which a hunter rewarded others contributing to the killing of an animal. In this case, the person owning the dog, net, or shotgun or acting to secure the kill would receive a cut of meat (080921-C, 190821-B).

After a successful individual hunt (e.g., shotgun) or trap visit, a hunter would bring home game to be butchered discreetly by himself or one of his sons, but never by household women. The initial butchering was always a male activity, permitting the senior man to organize meat sharing; women traditionally managed secondary cuts of already-butchered meat when they cooked (310319-B). Guided by the kinship-debt logic, the hunter would then send fresh meat parts or organs of medium-sized animals to specific relatives, consistently privileging parents and maternal kin. A hunter killing a monkey with a shotgun would send its heart to his mother, kidneys to his father, chest to his maternal uncle (external to the patrilineage), and neck to his brothers (090921-C, 140421-C, 310319-B). The hunter and his household would consume the remaining animal parts (090921-C).

Hence, the most plausible exposures to a freshly killed or dead animal were concentrated within this kinship-based, non-commercial circuit: the hunter or son butchering the animal, his mother, father, maternal uncle, brothers, as well as the children cooking these organs. Kinship debt-sharing thus strengthened kinship ties but resulted in recurrent exposures to game tissues and bodily fluids.

The second participation-based logic of meat sharing, which compensated contributions to a successful hunt, also applied to net hunting, which occurred less frequently than individual hunting. Collective net hunting entailed animal bodily fluid exposure for a wider range of participants. Before returning home from a hunt, net hunters butchered all game killed at the forest edge, grilled some animal parts for a celebratory meal (*ozamba*) before sharing the rest among themselves according to precise rules. Sharing rules were based on net hunt participation, skill, and labor. The hunting dog’s owner had the customary right to the animal neck; the net owner received its head, back, and hide (if the animal died in his net); the person first finding the caught animal received its chest (190821-B). The collective effort to butcher and split all carcasses into small shares to be distributed (see Fig 5) involved many men in contact with hunted animal carcasses and bodily fluids.

**Fig 5:**
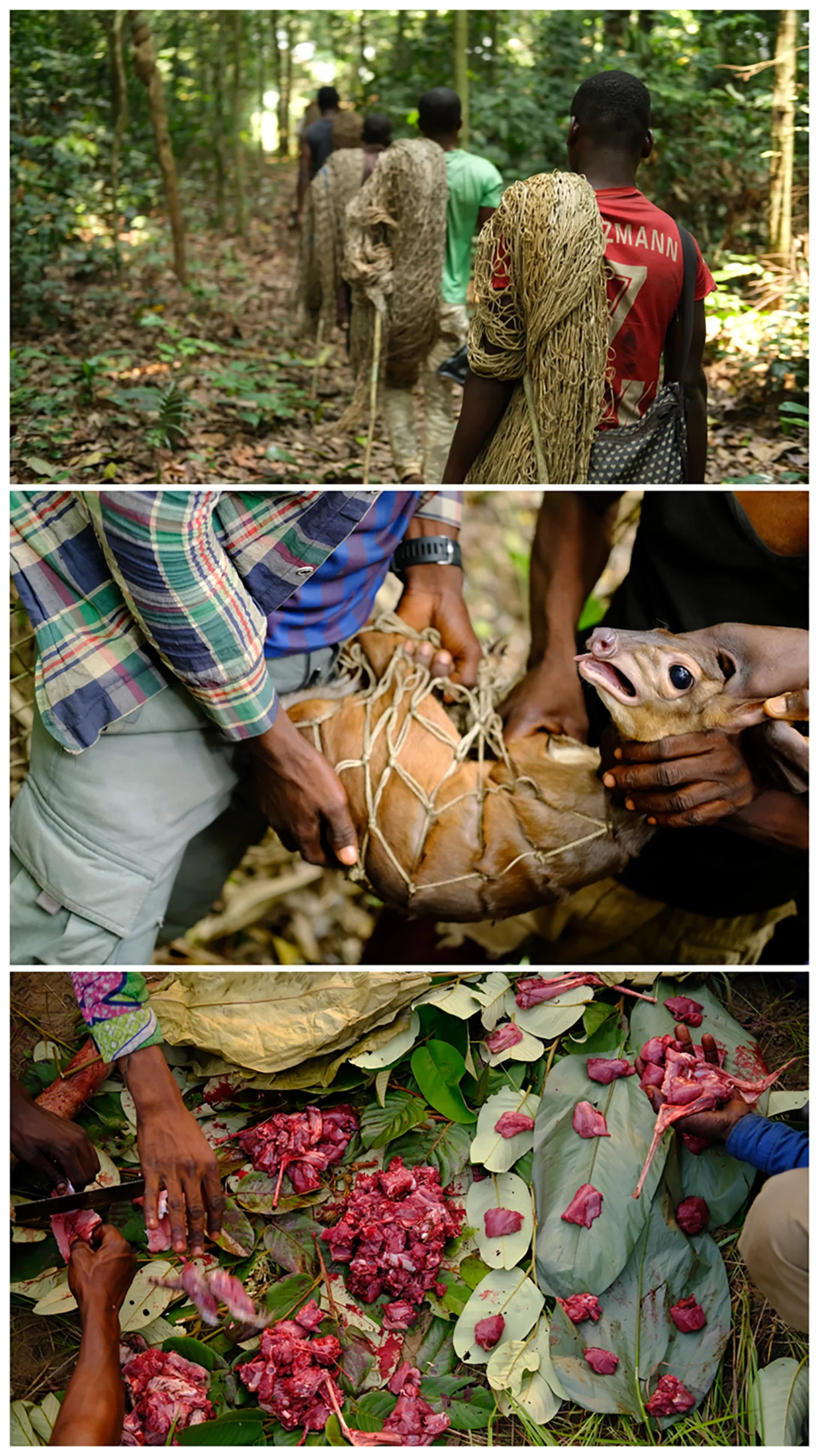
Contact with Cluster 2 animals (top: men leaving for net hunt; center: a duiker caught in a net; bottom: post-net hunt meat sharing (*ozamba*)) (credit: Romain Duda).

In general, larger wild meat led to a more extensive sharing and selling of smaller pieces among a larger group, whereas small animals were consumed among immediate family members. Moreover, in response to meat scarcity, participants indicated that hunters often hid small animals from the community to avoid jealousy and demands to share, so that sharing networks may be diminishing in response to declining animal populations. Kin group members exposed to a carcass may decrease with dwindling animal populations (090419-B, 080419-B).

Finally, inhabitants finding animals visibly suffering from illness would kill and eat them. They might also consume game found dead in the forest after examining it if they concluded that it was not too decomposed (190921-A). This practice could also entail exposure to animal bodily fluids.

#### Infrequent contact frequency with cluster 1 animals.

Cluster 1, which included some 46 animal species, was characterized by lower environmental and physical contact frequencies in comparison to the other clusters. Our relative abundance calculations for 17 species in this cluster revealed a significantly lower relative abundance compared to cluster 2 species (Mann-Whitney test, p < 0.05) and a tendency with cluster 3 species (Mann-Whitney test, p = 0.068). Certain cluster 1 species, however, had higher relative abundance compared to certain cluster 2 and 3 species but much lower contact frequencies.

Analyses of our ethnographic evidence provided additional insight into low contact frequencies with cluster 1 species. Here the term *ngeyi* shaped participants’ avoidance of these animals, in addition to social norms, family or individual sensitivities and past experiences with specific animals. Overall, participants labeled about 20 animal species as *ngeyi* (disgusting, repulsive*)*. Among these species, 6 appeared in cluster 3 (five “animals of the village”, discussed above, and *Mastomys natalensis*, a mouse present in houses); the other 14 appeared in cluster 1 (Fig 6). They included four carnivores (*Nandinia binotata, Civettictis civetta, Genetta sp., Herpestes sp.*), three primates (*Pan paniscus, Perodicticus* potto, *Galago sp.*)*,* but also *Lemniscomys sp., Petrodromus tetradactylus, Aonyx capensis, Varanus niloticus, Dendrohyrax dorsalis, and Pipistrella sp.*. Certain small terrestrial mammals (*Lemniscomys sp., Petrodromus tetradactylus*), although considered “dirty” by adults, were nonetheless often hunted and eaten by children.

**Fig 6:**
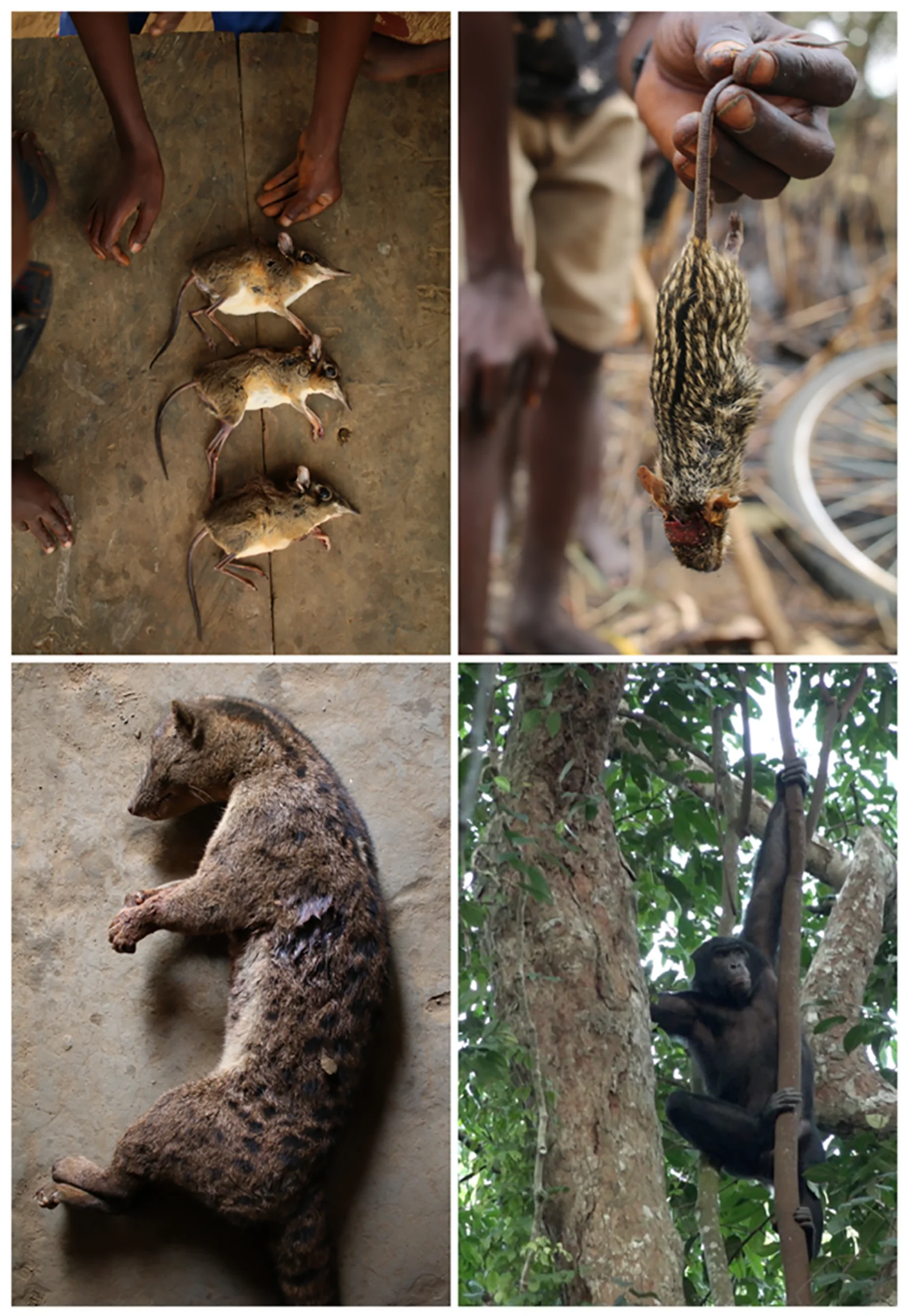
Selected cluster 1 animals considered *ngeyi* (top left: *Petrodromus tetradactylus*; top right: *Lemniscomys sp*.; bottom left: *Nandinia binotata*; bottom right: *Pan paniscus*) (credit: Romain Duda).

Participants used *ngeyi* to describe animals with a physical shape, behavior, or odor that is “abnormal” compared to other animals. Cluster 1 has many such animals. Pottos and galagos, for instance, are nocturnal animals with distinctive physical characteristics, notably their large eyes which local populations associate with sorcery and capacities to see the invisible. Animals inhabiting multiple ecosystems (e.g., monitor lizards and otters living in water and on land) were *ngeyi* because they were neither exclusively terrestrial nor aquatic. Carnivore species (locally categorized as *nyama a ka*, or “animals with padded paws”, which also include dogs and cats), too, were also described as *ngeyi*. Participants insisted that carnivores smelled bad, that they had relations with the spirit world and thus were suspicious because they could “see things” differently, that is, view malicious spirits (040819-A). Men and women volunteers did not display significant differences in contact with them, but women consumed these species half as frequently as men.

Inhabitants of the three villages avoided certain species for other reasons. Pregnant women, for instance, steered clear of monkeys so that their newborns would not resemble one (190921-L). Adults typically avoided bonobos (*Pan paniscus*; *ebubu*), hyrax (*Dendrohyrax dorsalis*; *ebuya*), and Potto (*Perodicticus potto*; *ekaru*) because these animals lacked a tail, which participants contended rendered them more like humans. Such avoided animals tended not to be hunted. With the exception of bonobos, such avoidances were reported to be more respected in the past than among current younger generations. Local myths accorded bonobos a special place, insisting that they had human forms in ancient times (020419-C; 290319-A; 300319-B). As one participant recounted,

“The ban on eating the bonobo has been in place since time immemorial, because the bonobo has characteristics resembling those of humans. It is said that the bonobo was originally a human who fled into the forest and avoided living with people in the village. And all animals have tails except the bonobo. Its hands look like a human’s. It also has a bad smell...” (290319-A)

A few cluster 1 species were an exception to the abundance-contact relationship identified in our models. *Civettictis Civetta*, for instance, figured among the abundant species recorded in our transects but fell into the cluster defined by lowest contact frequency. This result is better explained by local categorization of these animals as *ngeyi*.

## Discussion and conclusions

Overall, our participatory activity and contact survey revealed that across the three villages, participants were in physical and environmental contact with 61 different animal species. We categorized animal species according to their contact profile with humans, without preconceived categorization. Our modeling analysis of gender, village, relative abundance and human activities on animals clustered by contact frequency yielded few predictors of contact frequencies. It predicted proximate mechanisms (species abundance and proximity, hunting for physical contact with cluster 2 animals). But our historical analysis of land use and anthropological analysis of culturally-informed practices help elucidate why these factors operate on contact profiles. The following addresses these findings in engagement with broader literatures on human-animal contacts and their implications for zoonotic pathogen exposure.

### Human activities

We initially evaluated whether activity frequencies of participants differed by village and gender. Here we found no differences in activity frequencies between the three villages. Women performed agriculture and gathering more frequently than men across the three villages. Although these forest edge societies do display an important gender division of labor, we found that women also contributed to hunting activities, frequently visiting snare traps set by their husbands near fields and bringing home small, trapped mammals.

Our findings largely resemble the range of human activities in forest societies in central Africa [44–47], although the proportions of labor expended for agriculture, hunting, gathering, and other activities may vary across these forest societies. Nonetheless, whereas some studies of human activities in the forest have oversimplified the range of practices, we show that our participants performed wide-ranging forest activities. Hunting practices, from hunting with dogs to shotgun hunting, reflect those elsewhere in central Africa. In addition, although women’s hunting practices have frequently been overlooked in hunting literatures in Africa, multiple studies have reported such activities among women [48–50].

### Diversity of contact with animal species

We found that our participants were in physical and environmental contact with highly diverse animal species (n = 61), with a higher number of contact events and diverse species per day in Village A than in the other villages. This distinctive feature of Village A animal contacts resulted from higher contact frequencies with domesticated animal species in a more fragmented forest, and where small-scale sheep and goat production was more developed because of proximity to the riverine market town.

Here our results depart from and convene to a broader literature on human-animal contact. Older contact studies have tended to evaluate human contacts with selected species or animal host reservoirs of specific pathogens, thus limiting the diversity of animal species documented [14,33,51–53]. More recent contributions have expanded analyses of human-animal contacts to account for a range of domesticated and undomesticated animals [16,54,55]. The high diversity that we identified may differ from prior studies for multiple reasons: some studies develop data collection tools that request participants to report only their contacts with selected animal species; others aggregate animal categories (e.g., “monkeys”, rather than specific nonhuman primate species); still others may have been conducted in sites with less animal diversity.

### Clustering of animal species by contact

Rather than imposing pre-conceived categories on our analyses of human-animal contact frequencies, we categorized each animal species with which our participants had contact by their contact profile. Our HCA analysis yielded clustering into three distinct groups: cluster 3 animals, with a high frequency of environmental contacts (including the commensal rodent species *Mastomys natalensis* and all domesticated species except for pigs, which are rare in this region); cluster 2 animals, with high physical contact rates (which included a large rodent and small mammals); and cluster 1 animals, a wide range of undomesticated mammals with which our participants had infrequent physical or environmental contact.

This clustering departs from the human-animal contact literature, which has targeted selected animal species or pre-determined aggregated categories of animals [16,54,55]. Elsewhere, we have argued for abandoning analyses based on pre-conceived categories of “wild” and “captive” animals, which are neither homogeneous nor stable across diverse settings [56]. Here we would extend our reflections, arguing that we should use category labels critically and consider how grounded experiences can shed light on alternative categorizations. Although our cluster 3 species were roughly comprised of “domesticated” and commensal rodent species and cluster 2 species were frequently hunted mammals, these clusters emerged from nearly real-time human contacts, evaluated by frequency and type. The highest risk of pathogen exposure would, we anticipate, occur between humans and clusters 3 and 2 animals based on prior studies examining potential risks resulting from human-animal contact [16,57,58].

These clusters thus reveal critical insight into human exposures to zoonotic pathogens over time through the types and frequencies of contact with domesticated, commensal, and undomesticated animals. Although this article does not report on the pathogens carried by animal species in the three village sites and transmitted to human inhabitants, we show the real, cumulative risks of exposure to pathogens posed by animal clusters over time. Pathogenic exposure associated with cluster 1 animals, for instance, were likely lower than for other animals because our participants rarely had physical or spatial contact with these species. By contrast, exposure to pathogens through physical contact substantially increased with cluster 2 species because our participants hunted, butchered, and prepared them most frequently. Participants would be more exposed to environmentally persistent pathogens with cluster 3 species because of their frequent spatial contact with such species. Cluster 3 species can potentially carry pathogens to which village inhabitants may be exposed, including avian influenza from poultry, Q fever from small ruminants, and leptospirosis from commensal rodents. Elsewhere, transmission appears facilitated with frequent exposure [59–61].

Pathogen sharing can result from a single, rare contact, but the emergence of transmissible human infections has more often depended on repeated exposure over time. The example of simian immunodeficiency virus (SIV) spillover from chimpanzees to humans, setting the foundations for the HIV-1M pandemic, is telling. Phylogenetic evidence indicates that SIV from nonhuman primates infected humans on multiple occasions. Most of these spillovers produced no sustained human transmission, and central African forest inhabitants had hunted and butchered nonhuman primates for millennia without apparent incident. What changed in the early twentieth century was not contact with primates alone, but the addition of repeated parenteral exposures among human hosts [62,63], as colonial campaigns against sleeping sickness administered injections with unsterilized equipment on a mass scale. Contact frequency did not just increase the probability of a rare event; it shaped the very conditions under which that pathogen adapted to it new human host.

Our analysis offers potential guidance for animal surveillance systems in low-income countries with limited labor and resources to invest in such activities, by prioritizing the monitoring of species with which humans have most frequent physical or environmental contact. Targeting animal contact profiles could be a more effective means of monitoring animal populations in low-resource settings, although further research is needed to determine its effectiveness.

### Factors influencing contacts and potential risk of zoonotic pathogen exposure

Among the variables tested in our multivariate model, we identified two ecological factors explaining contact frequency, although other factors could not. We found no effect of gender on contact frequency in the significant models for each animal cluster, and no clear pattern for an effect of the village.

Frequencies of human activities were associated with physical contact frequencies with cluster 2 species. We found no differences in activity frequencies between our three villages. Our finding concerning gender on contact frequency may result from the fact that women performed agriculture and gathering most frequently across the three villages and contributed significantly to hunting activities. Forest edge societies do display an important gender division of labor, but women’s participation in hunting (by checking traps) echoes findings of studies elsewhere in central and west Africa [47–50,64].

We also found that relative abundance of animal species is associated with contact frequency: those species in higher abundance and closer to villages have higher contact frequency with humans. This result converges with a prior study on contact with nonhuman primates in Cameroon [15]. This effect, however, is weak and cannot fully explain the factors shaping human-animal contact. The results of our multivariate model illustrate that human-animal contact frequency is highly complex and difficult to distill into a discrete set of independent variables. These results highlight that modeling offers limited explanation for contact frequencies.

### Historical changes and contemporary understandings and practices shaping human-animal contacts

Other analyses, unable to be captured in multivariate models, assist us in understanding the contact profiles and frequencies identified. Our ethnohistorical and ethnographic analyses suggest that longer-term historical processes and contemporary sociocultural understandings of and practices around specific animals shaped human-animal contact, and more broadly, potential exposures to zoonotic pathogens. Such analyses, frequently neglected in contact studies, yield crucial light on why contact profiles clustered as they did.

Our ethnohistorical analyses revealed that changing animal raising practices and land use contributed to patterns of human contact with diverse animal species. Allowing domesticated animals to range freely is widely practiced throughout the African continent, so it is not surprising that cluster 3 composition reflected frequent environmental contacts [65]. The advent of large- and small-scale cattle farming from the mid-twentieth century appears not only to have shaped these contacts, but to have generated other alterations in land use, ecological zones, and human-animal contact. Our oral histories revealed that cattle farming precipitated substantial land use changes, transforming savannas into grazing lands for cattle, and leading to significant dispossession of arable savanna lands among women and the displacement of women’s cultivation and hunting activities from savannas into forest sites. These land use changes, our participants recounted, affected forest farming practices (shorter fallows) and additional ecological transformations, notably “dead forests” with different botanical composition and populated with certain rodent species, (brush-tailed porcupine, Emin’s pouched rat, cane rats, and small carnivores). Our participants had considerable physical contact with the former two species, belonging to cluster 2. Hence, the introduction of cattle raising appears to have had long-term ecological consequences, which in turn affected human-animal contact and potential increased risks in zoonotic transmission [26]. Our analysis reveals, then, that hunting is not the only factor shaping human-animal contact, proposing that changes in land use and animal husbandry can reshape the frequencies and types of contacts with specific animals.

Nevertheless, hunting pressures may have contributed to frequent human contact with these large rodents. Our older participants contended that overhunting depleted large, undomesticated animals, the consequence of the democratization of hunting rifles, as observed elsewhere [66]. This reported disappearance of larger animal species may explain why our participants had infrequent contact with wide ranging animal species (cluster 1) and frequent physical contact with small mammals (cluster 2). In Africa and elsewhere in the world, land use and animal husbandry changes, as well as long-term hunting intensification have been shown to transform the profiles of animal populations [67]. The replacement of large mammals by smaller ones echoes insights from other studies; large scale investigations show that hunting pressures and land use changes reduce nonhuman mammal diversity, facilitating increasing populations of robust, rapidly reproducing small mammals, including rodents [68–72].

In addition, human-animal contacts also seem to be shaped by local ways of perceiving, categorizing and using animals. Meat sharing logics affected different social groups in different ways, concentrating potential risk of exposure to zoonotic pathogens of frequently hunted animals among close kin of hunters (kinship-debt logic), but also among male hunters, including net hunters (participatory logic).

Our participants explained that they actively avoided certain animal species that they considered *ngeyi* – “dirty”, and thus not to be touched or eaten. These avoidance practices could explain the lack of physical contact with domestic animals and commensal rodents, as well as highly infrequent contact with certain cluster 1 animals. Several studies elsewhere in central Africa have explored the sociocultural understandings affecting contact with animals [73–76], in which animals’ unique behavioral, ecological, or morphological characteristics (e.g., strong odors or taste, semi-aquatic) lead certain societies to give them prominence in their local classifications; they are thus considered unsuitable for contact and consumption [77]. Consumption of these animals may also be locally associated with negative health consequences for oneself or one’s unborn child [74,78]. Elsewhere in central Africa, domesticated animals are capital, and thus not necessarily understood as “good to eat” compared to wild meat because of their spatial proximity to humans. Multiple societies associate the forest with purity but perceive the village as a space of accumulating waste and excrement to which domestic animals are exposed [74,79,80].

Overall, this multidisciplinary investigation yields broad, substantive insight into the diversity of human contacts with animals in this fragmented forest zone. Standard variables (animal relative abundance, activity frequency, village site) were associated with contact frequency but explained only a small portion of it. Longer-term histories of land use change and hunting intensification and sociocultural practices of avoidance helped to shed light on these contact frequency profiles.

These findings have implications for animal surveillance in resource-constrained settings. Exposure to zoonotic pathogens is not equally distributed across animal species, nor across contact profiles; inhabitants of our study zone had frequent contact with a relatively small group of animals, both domesticated and undomesticated. Prioritizing surveillance of cluster 2 and cluster 3 species would concentrate limited effort on those animals generating the greatest cumulative number of transmission opportunities. Low contact frequency does not eliminate risk. Rare contacts with cluster 1 species could produce consequential spillovers, and thus broad pathogen discovery is valuable. But in resource-constrained settings where surveillance capacity is limited, contact profiles offer an empirically grounded basis for prioritization, identifying species for which repeated exposure generates the greatest cumulative number of transmission opportunities.

More broadly, our findings show that human-animal contact profiles are historically produced and socioculturally mediated. “Contact” is not a singular, discrete event, but rather shaped by larger socioecological processes and sociocultural perceptions and practices [12]. Hence, contact profiles cannot be derived from species ecology, land cover analyses, or demographic variables alone; similar ecological configurations may yield divergent contact profiles depending on the histories and practices of the human communities inhabiting them. Anticipating how zoonotic exposure will shift as social and ecological conditions continue to change therefore requires attending to these processes, not just to their present-day traces.

### Limitations of the study

This study has several limitations that necessitate some caution in interpreting our results. First, our participatory contact survey had some important shortcomings. The sample size of volunteers was relatively small and biased in favor of men. It cannot be considered representative of the three village populations nor of the province, even though our limited sample generated a considerable quantity of data. Future studies could employ less labor-intensive tools to cover a representative sample of village and provincial populations. Limited participation among women may have negated any gender influence in the models. A species-level analysis of gender effects lies beyond the scope of this study. Our study also does not address children’s diverse contacts with animals and consequently neglects exposure risks to zoonotic pathogens among young age groups [81].

Our contact study covered only five months, and thus our activity and animal contact data could over- or underestimate the importance weight of certain activities or animal contacts. Nevertheless, inhabitants of these village sites conduct farming, hunting, and fishing year-round, even if specific techniques associated with these activities changed across seasons. Although most animal species reported here engaged in limited seasonal mobility, they displayed no major changes in distribution area and habitat use.

Our participatory contact survey generated highly granular, near real-time reporting of contacts, but it depended on participants’ individual ecological knowledge and their attention to and recollection of animals and animal traces encountered. We sought to compensate for inattention and recall problems by careful training of volunteers and frequent visits from a field supervisor to ensure robust field data. Finally, this tool relies on a highly constrained definition of “contact”, that is, physical or spatial. Local cultural understandings of contact were not integrated into the tool. Forest inhabitants frequently use their sense of smell to detect or identify animals and plants and consider odor to be a form of interspecies contact. Nevertheless, we concluded that integrating other understandings of contact would render our participatory collection even more complex than it already was, thus risking participant confusion and generating less reliable results.

Our animal contact data contained some omissions. We did not evaluate contact with non-domestic birds, arthropods, and fish, which could have yielded a wider array of contacts and potential pathogenic exposures. Nevertheless, including these animals would have entailed a vastly different protocol that extended well beyond project capacities and resources. Our identification of house-dwelling bats relied on household reporting rather than capture and identification of bats; our survey therefore cannot exclude the presence of other bat species in houses. Additionally, certain key animal species were missing from our transect survey. Heavy workloads and limited resources prevented our field team from evaluating numerous bat or monkey species. Future investigation should include these species.

The presence of a bonobo conservation project may have influenced participant responses, particularly about declining animal populations and prohibitions on bonobo hunting and consumption. We took several steps to mitigate this limitation. Interviews were conducted by researchers with long-standing relationships in these communities and participants were assured of confidentiality. We cannot exclude some influence on accounts concerning bonobos specifically, given that avoidance of bonobos is both a long-standing local practice and an object of ongoing conservation messaging.

Finally, this article addresses only risks associated with contact, not actual exposure to and infection with zoonotic pathogens. Such epidemiological and serological analysis is beyond the scope of this study.

## Supporting information

S1 File Data collection tools. (DOCX)

S2 File R script and results of the generalized linear mixed models. (PDF)

S1 Table Description of number and diversity of animal contacts and statistical results for the comparisons between gender and villages. (XLSX)

S2 Table Descriptive statistics for contact frequencies with each species and each type of contact for the total volunteers, by gender and by village. Detailed statistical results are shown for gender and village comparisons. (XLSX)

S3 Table Cluster assigned, vernacular name and relative abundance for each animal species. (XLSX)

S4 Table p-values of Kruskal-Wallis and Wilcoxon tests for comparisons of mean contact frequencies for each type of contact between clusters. (XLSX)

S5 Table Descriptive statistics of contact frequencies for each type of contact and each cluster. (XLSX)
