# Supplementary material for: Contact in context: How land use histories, sociocultural practices, and animal profiles shape human-animal contact and zoonotic pathogen exposure in a fragmented forest of the Democratic Republic of Congo
Source: PLoS Negl Trop Dis. 2026 Oct 26;20(10):e0013823. doi: 10.1371/journal.pntd.0013823 (PMC13727040; doi:10.1371/journal.pntd.0013823)
Supplement: Supplementary file 1 [file pntd.0013823.s002.pdf]

Supplementary Information 2: R script and results of the Generalized Linear Mixed Models

Anonymized

Contents

Data wrangling **1**

GLMM analysis **6**
Cluster 1 6
Cluster 2 14
Cluster 3 22

Comparing models **31**

Data wrangling

Basic settings, e.g., we read the basic packages and set the seed for reproducibility. The most relevant is the tidyverse work environment. See https://www.tidyverse.org/ for more details.

```
# load basic packages
library(ggplot2)
library(lmerTest)
```

```
## Loading required package: lme4

## Loading required package: Matrix

##
## Attaching package: 'lmerTest'

## The following object is masked from 'package:lme4':
##
##     lmer

## The following object is masked from 'package:stats':
##
##     step
```

```
library(caret)
```

```
## Loading required package: lattice
```

```
# packages for interpreting mixed models
library(sjPlot)
```

```
##
## Attaching package: 'sjPlot'

## The following object is masked from 'package:ggplot2':
```

```
##
##     set_theme
```

```
# set seed for reproducibility
set.seed(10)
# set visual options for numbers
options(scipen=999)
```

We read the basic data

```
# read the data
data_glmm = read.table("Dataset_contacts_Microtone_pour_modele.csv", sep=";", header=T)
# change some columns to factors
data_glmm$ID_vol=as.factor(data_glmm$ID_vol)
data_glmm$Village=as.factor(data_glmm$Village)
data_glmm$Contact_type=as.factor(data_glmm$Contact_type)
data_glmm$Sexe=as.factor(data_glmm$Sexe)
data_glmm$Animal_Species=as.factor(data_glmm$Animal_Species)
data_glmm$Cluster_CAH=as.factor(data_glmm$Cluster_CAH)
data_glmm$ACP_contact=as.factor(data_glmm$ACP_contact)

# visual check
str(data_glmm)
```

```
## 'data.frame':    13896 obs. of  14 variables:
##  $ Contact_type      : Factor w/ 14 levels "ACH","CRI","CRO",..: 2 3 9 14 1 2 3 5 6 9 ...
##  $ ACP_contact       : Factor w/ 2 levels "envt","physique": 1 1 1 1 2 1 1 1 2 1 ...
##  $ ID_vol            : Factor w/ 24 levels "v1","v10","v11",..: 1 1 1 1 1 1 1 1 1 1 ...
##  $ Village           : Factor w/ 3 levels "Village_A","Village_B",..: 3 3 3 3 3 3 3 3 3 3 ...
##  $ Sexe              : Factor w/ 2 levels "F","M": 2 2 2 2 2 2 2 2 2 2 ...
##  $ Animal_Species    : Factor w/ 61 levels "Anomalurus sp.",..: 1 1 1 1 2 2 2 2 2 2 ...
##  $ Freq_contact      : num  0 0 0 0 0 0 0 0 0 0 ...
##  $ Relative_abundance: num  NA NA NA NA NA NA NA NA NA NA ...
##  $ Cluster_CAH       : Factor w/ 3 levels "1","2","3": 1 1 1 1 1 1 1 1 1 1 ...
##  $ Hunting_freq      : num  0.386 0.386 0.386 0.386 0.386 ...
##  $ Fishing_freq      : num  0.114 0.114 0.114 0.114 0.114 ...
##  $ Gathering_freq    : num  0.651 0.651 0.651 0.651 0.651 ...
##  $ Fields_freq       : num  0.404 0.404 0.404 0.404 0.404 ...
##  $ Noforest_freq     : num  0.241 0.241 0.241 0.241 0.241 ...
```

We can visualize the distribution of the main variables

```
hist(data_glmm$Hunting_freq)
```

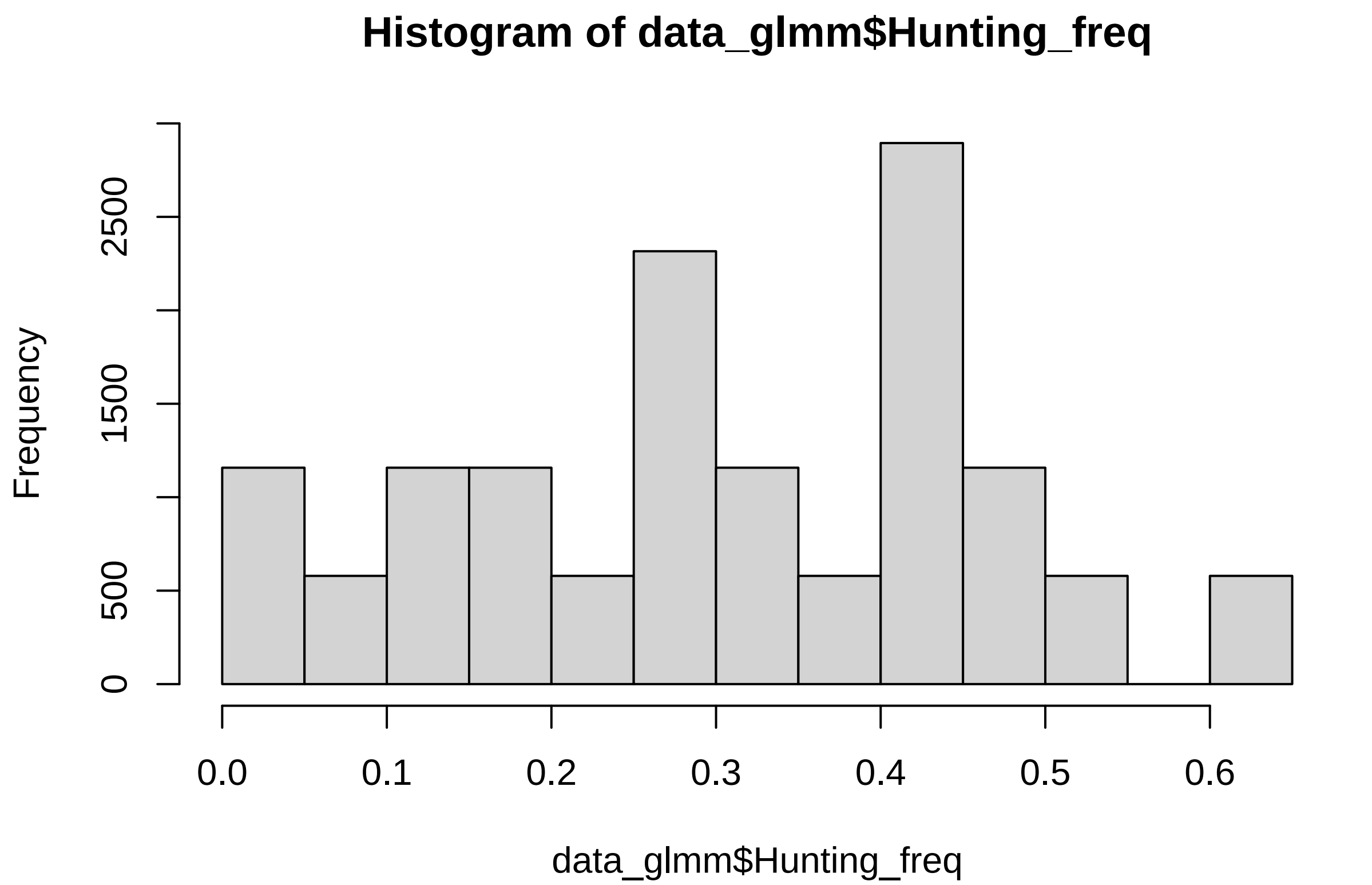


```
hist(data_glmm$Fishing_freq)
```

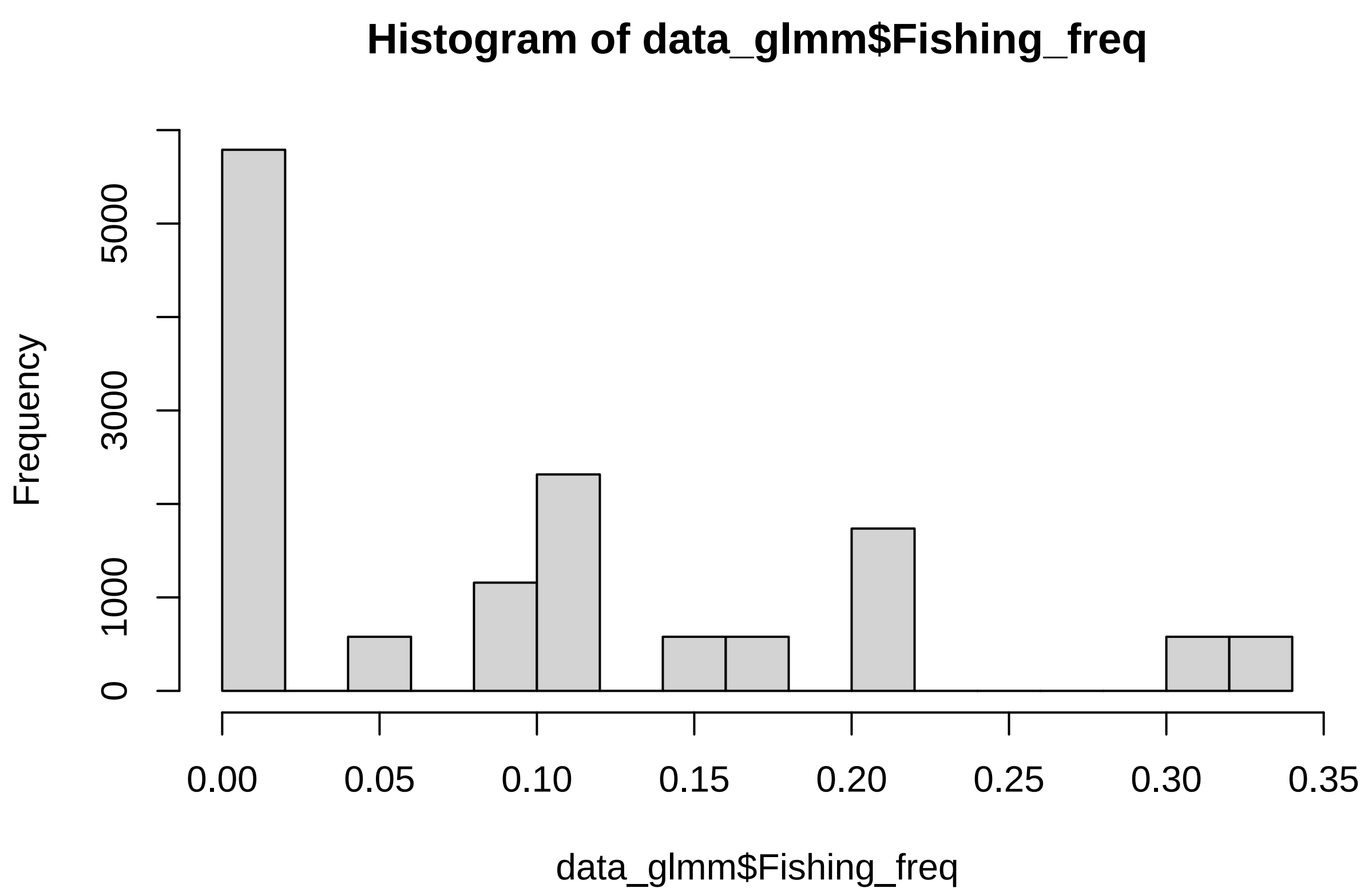


```
hist(data_glmm$Gathering_freq)
```

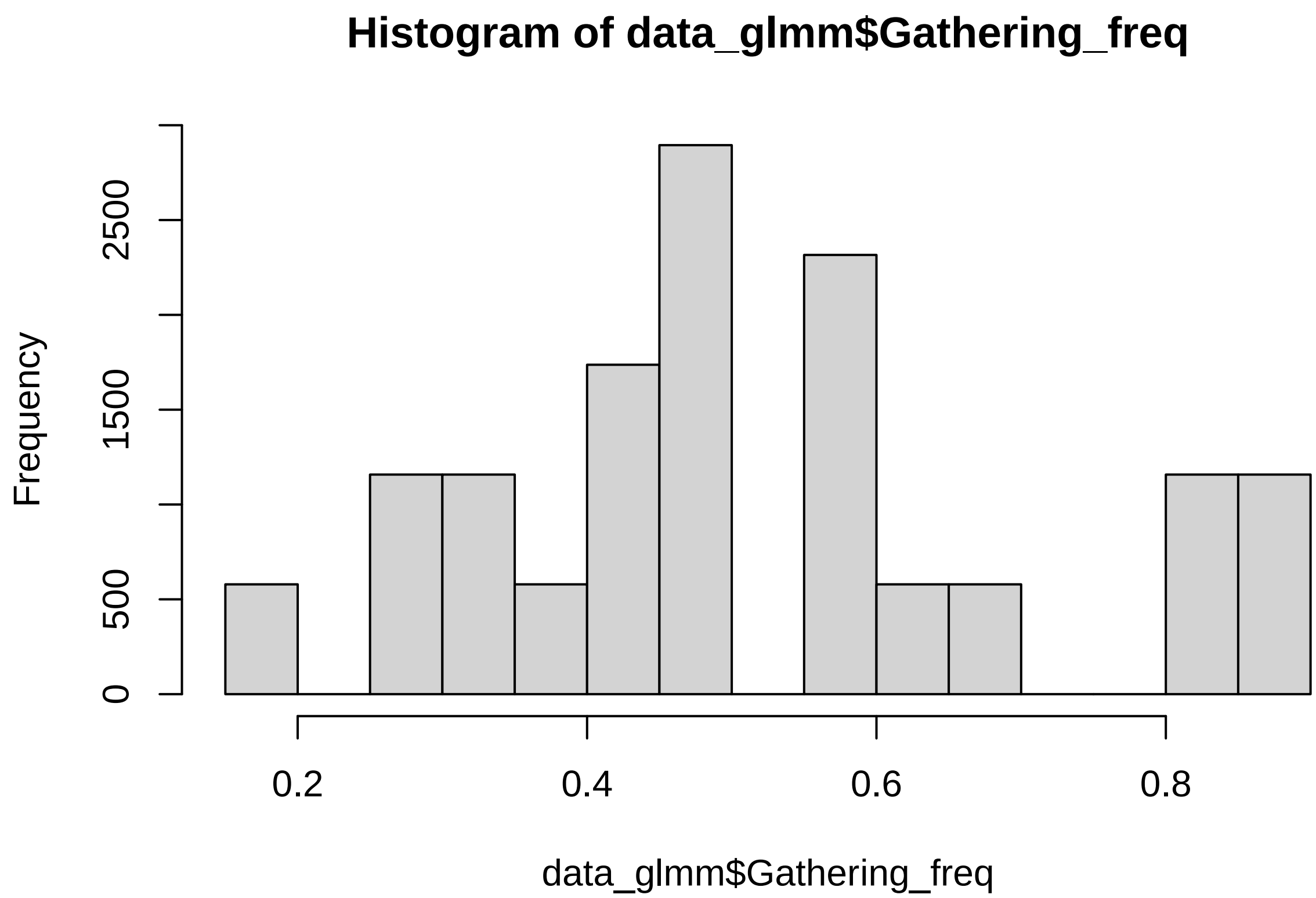


```
hist(data_glmm$Fields_freq)
```

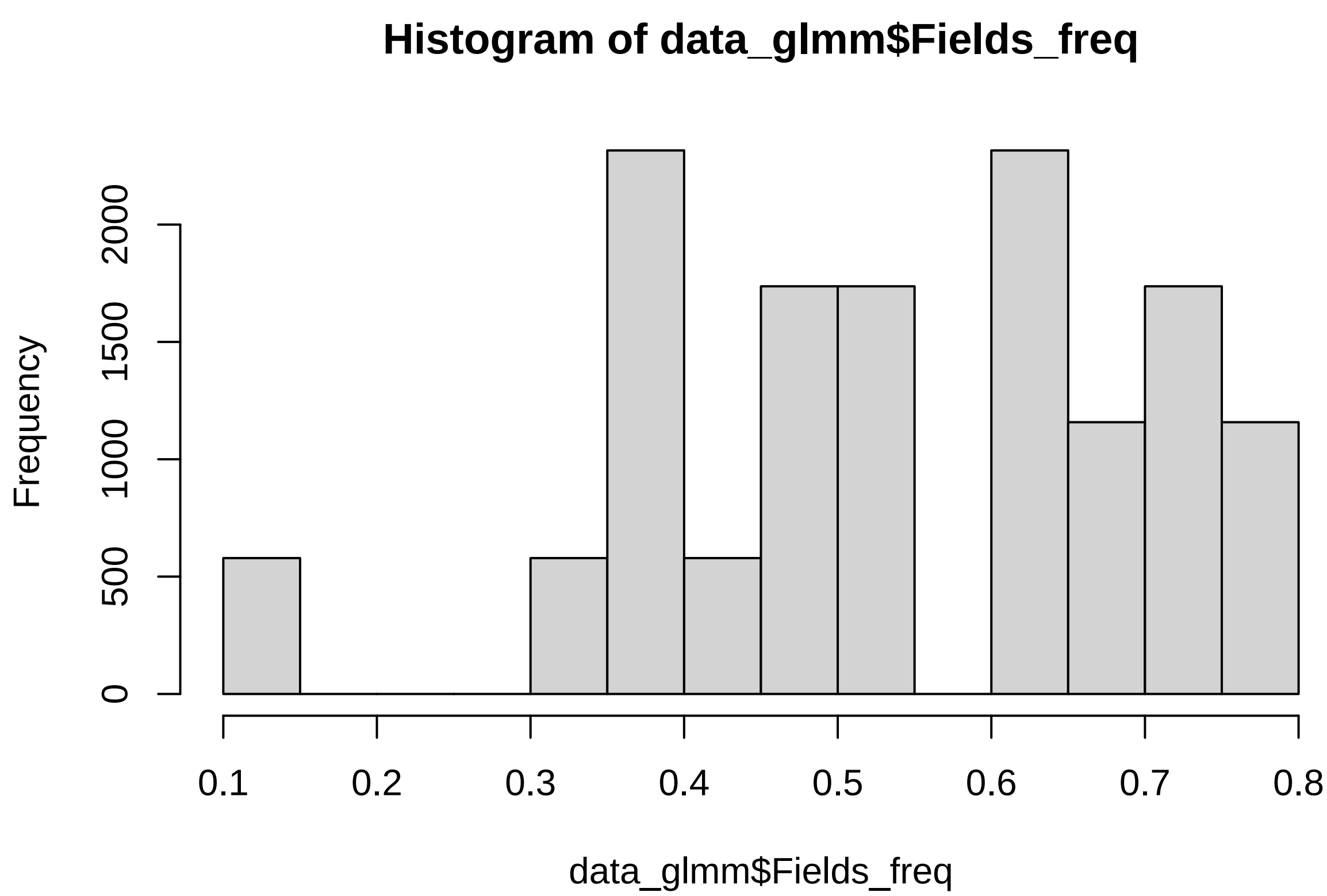


```
hist(data_glmm$Noforest_freq)
```

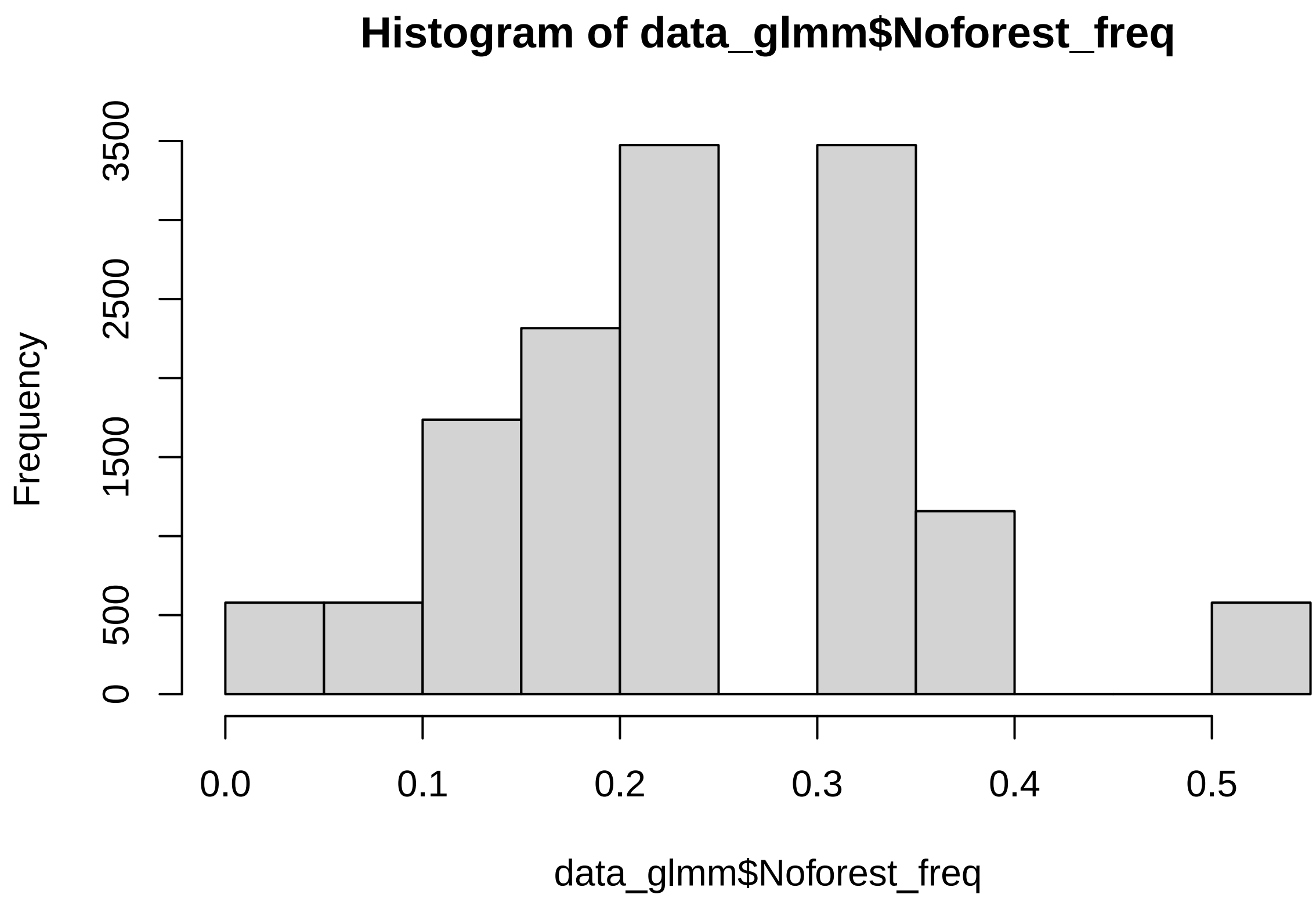


```
hist(data_glmm$Relative_abundance)
```

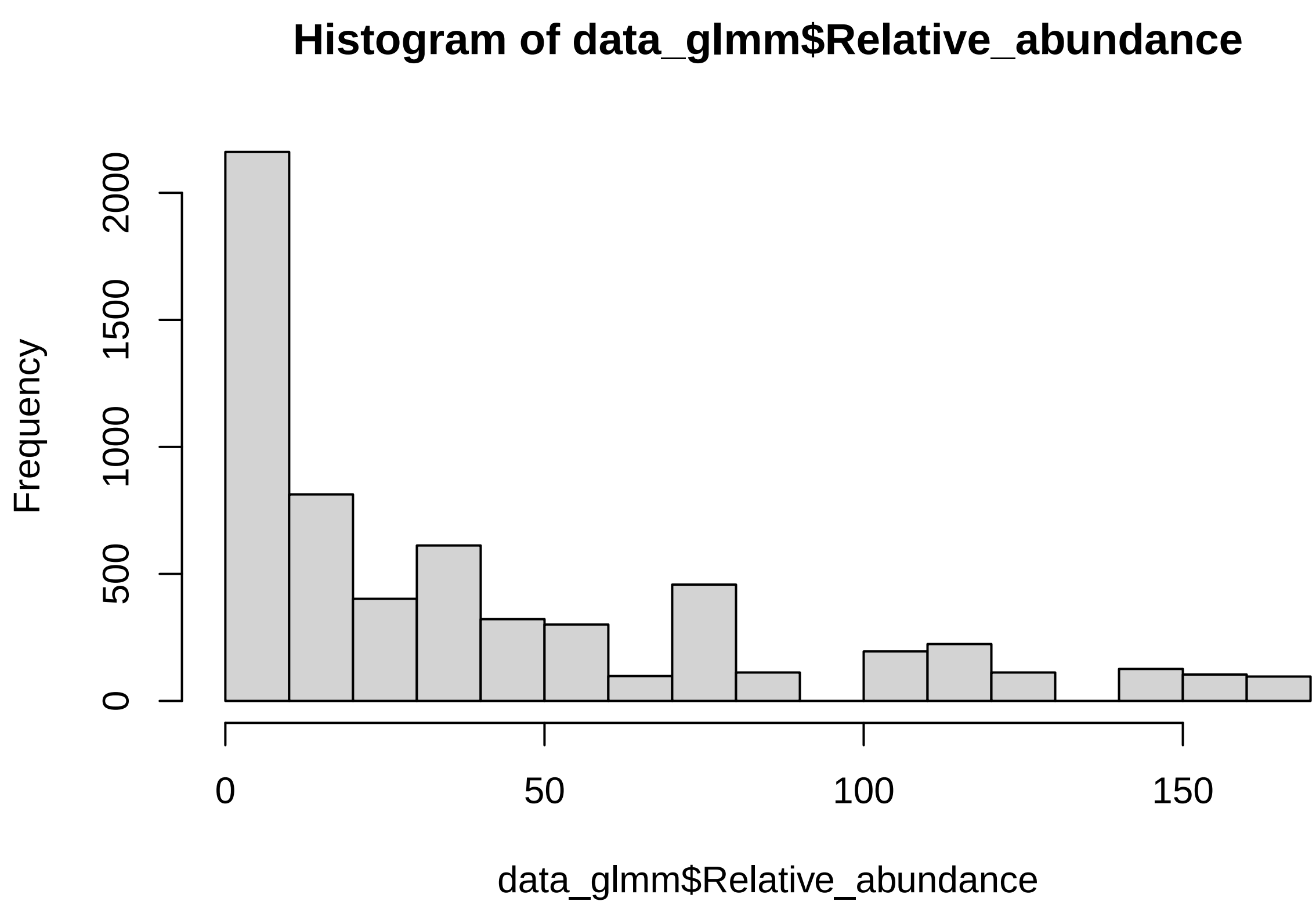


```
hist(data_glmm$Freq_contact)
```

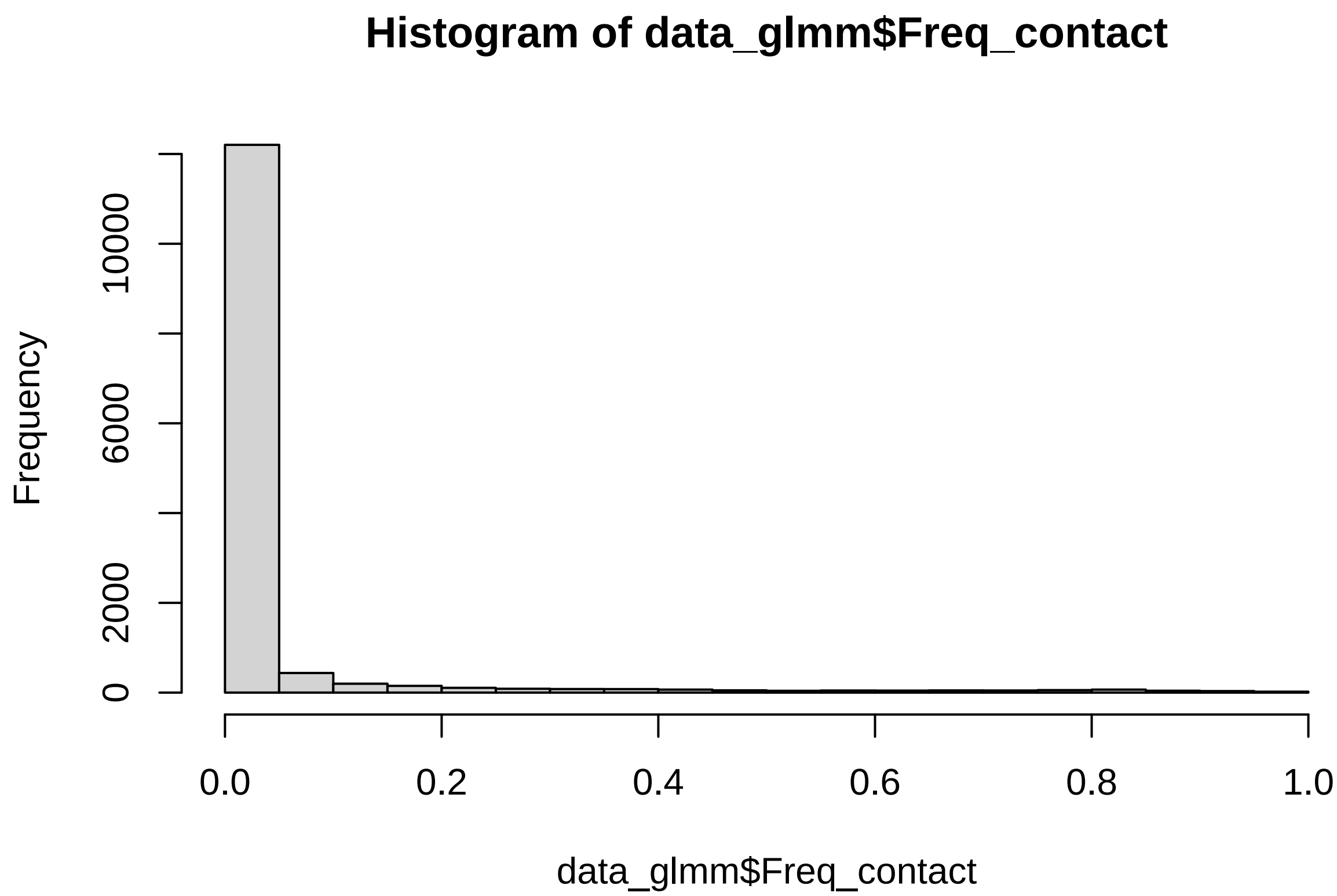

GLMM analysis

For each of the three clusters, we run two models: one for environmental contact and one for physical contact.

Cluster 1

envt

```
# set the data for cluster 1
data_glmm_RA_cluster1=data_glmm[which(data_glmm$Cluster_CAH=="1" & data_glmm$Relative_abundance!=""),]
# visual check
#str(data_glmm_RA_cluster1)

# make the model
glmm_cluster1_RA_envt=lmerTest::lmer(Freq_contact ~ Village + Sexe + Relative_abundance +
                                       Hunting_freq + Fishing_freq + Gathering_freq + Fields_freq +
                                       Noforest_freq + (1|ID_vol) + (1|Animal_Species),
                                     data=data_glmm_RA_cluster1[which(data_glmm_RA_cluster1$ACP_contact=
```

```
## boundary (singular) fit: see help('isSingular')
```

```
# model output
summary(glmm_cluster1_RA_envt)
```

```
## Linear mixed model fit by maximum likelihood . t-tests use Satterthwaite's
##   method [lmerModLmerTest]
## Formula: Freq_contact ~ Village + Sexe + Relative_abundance + Hunting_freq +
##     Fishing_freq + Gathering_freq + Fields_freq + Noforest_freq +
##     (1 | ID_vol) + (1 | Animal_Species)
##    Data: data_glmm_RA_cluster1[which(data_glmm_RA_cluster1$ACP_contact ==
```

```
##     "envt"), ]
##
##      AIC       BIC    logLik -2*log(L)  df.resid
##   -3459.7   -3392.6    1742.9   -3485.7      1276
##
## Scaled residuals:
##     Min      1Q  Median      3Q     Max
## -1.7390 -0.3491 -0.0940  0.0927  9.8183
##
## Random effects:
##  Groups         Name        Variance  Std.Dev.
##  ID_vol         (Intercept) 0.0000000 0.00000
##  Animal_Species (Intercept) 0.0008006 0.02829
##  Residual                   0.0037782 0.06147
## Number of obs: 1289, groups:  ID_vol, 24; Animal_Species, 18
##
## Fixed effects:
##                       Estimate   Std. Error          df t value  Pr(>|t|)
## (Intercept)          0.0184711    0.0196423  618.1169597   0.940    0.3474
## VillageVillage_B     0.0268868    0.0068024  652.9408060   3.953 0.0000858 ***
## VillageVillage_C     0.0079860    0.0079750  450.4908871   1.001    0.3172
## SexeM               -0.0059688    0.0058128 1270.3228466  -1.027    0.3047
## Relative_abundance   0.0006647    0.0001376  370.2610974   4.830 0.0000020 ***
## Hunting_freq        -0.0005069    0.0133090 1270.3228472  -0.038    0.9696
## Fishing_freq        -0.0048990    0.0261257 1270.3228473  -0.188    0.8513
## Gathering_freq      -0.0016659    0.0132530 1270.3228469  -0.126    0.9000
## Fields_freq          0.0108984    0.0158528 1270.3228465   0.687    0.4919
## Noforest_freq       -0.0447915    0.0216874 1270.3228456  -2.065    0.0391 *
## ---
## Signif. codes:  0 '***' 0.001 '**' 0.01 '*' 0.05 '.' 0.1 ' ' 1
##
## Correlation of Fixed Effects:
##             (Intr) VllV_B VllV_C SexeM  Rltv_b Hntng_ Fshng_ Gthrn_ Flds_f
## VillgVllg_B -0.353
## VillgVllg_C -0.287  0.582
## SexeM       -0.531  0.083  0.009
## Reltv_bndnc -0.181  0.524  0.678  0.000
## Hunting_frq -0.218  0.015  0.162 -0.320  0.000
## Fishing_frq -0.169  0.264 -0.153  0.274  0.000 -0.411
## Gathrng_frq -0.494  0.078 -0.053  0.240  0.000  0.285 -0.131
## Fields_freq -0.616  0.075  0.186  0.540  0.000 -0.122  0.081 -0.086
## Noforst_frq -0.660  0.301  0.114  0.084  0.000  0.241  0.269  0.329  0.294
## optimizer (nloptwrap) convergence code: 0 (OK)
## boundary (singular) fit: see help('isSingular')
```

We also check if the model is better than a null model.

```
# make the null model
null_cluster1_RA_envt=lmerTest::lmer(Freq_contact ~ (1|ID_vol) + (1|Animal_Species),
                                     data=data_glmm_RA_cluster1[which(data_glmm_RA_cluster1$ACP_contact=

# make the comparison
anova(glmm_cluster1_RA_envt, null_cluster1_RA_envt, test="chisq")
```

```
## Data: data_glmm_RA_cluster1[which(data_glmm_RA_cluster1$ACP_contact ==  ...
```

```
## Models:
## null_cluster1_RA_envt: Freq_contact ~ (1 | ID_vol) + (1 | Animal_Species)
## glmm_cluster1_RA_envt: Freq_contact ~ Village + Sexe + Relative_abundance + Hunting_freq + Fishing_f
##                       npar     AIC     BIC logLik -2*log(L)  Chisq Df
## null_cluster1_RA_envt    4 -3423.5 -3402.9 1715.8   -3431.5
## glmm_cluster1_RA_envt   13 -3459.7 -3392.6 1742.9   -3485.7 54.188  9
##                          Pr(>Chisq)
## null_cluster1_RA_envt
## glmm_cluster1_RA_envt 0.00000001738 ***
## ---
## Signif. codes:  0 '***' 0.001 '**' 0.01 '*' 0.05 '.' 0.1 ' ' 1
```

We visualize the fixed effects.

```
#png("plot.png", res=300, height=1100, width=1600)
sjPlot::plot_model(glmm_cluster1_RA_envt, type = "est", auto.label = FALSE,
                   vline.color = "green", sort.est = TRUE, show.values = TRUE) + ylim(-0.25,0.25)
```

```
## Warning: Using `size` aesthetic for lines was deprecated in ggplot2 3.4.0.
## i Please use `linewidth` instead.
## i The deprecated feature was likely used in the sjPlot package.
##   Please report the issue at <https://github.com/strengejacke/sjPlot/issues>.
## This warning is displayed once per session.
## Call `lifecycle::last_lifecycle_warnings()` to see where this warning was
## generated.

## Scale for y is already present.
## Adding another scale for y, which will replace the existing scale.
```

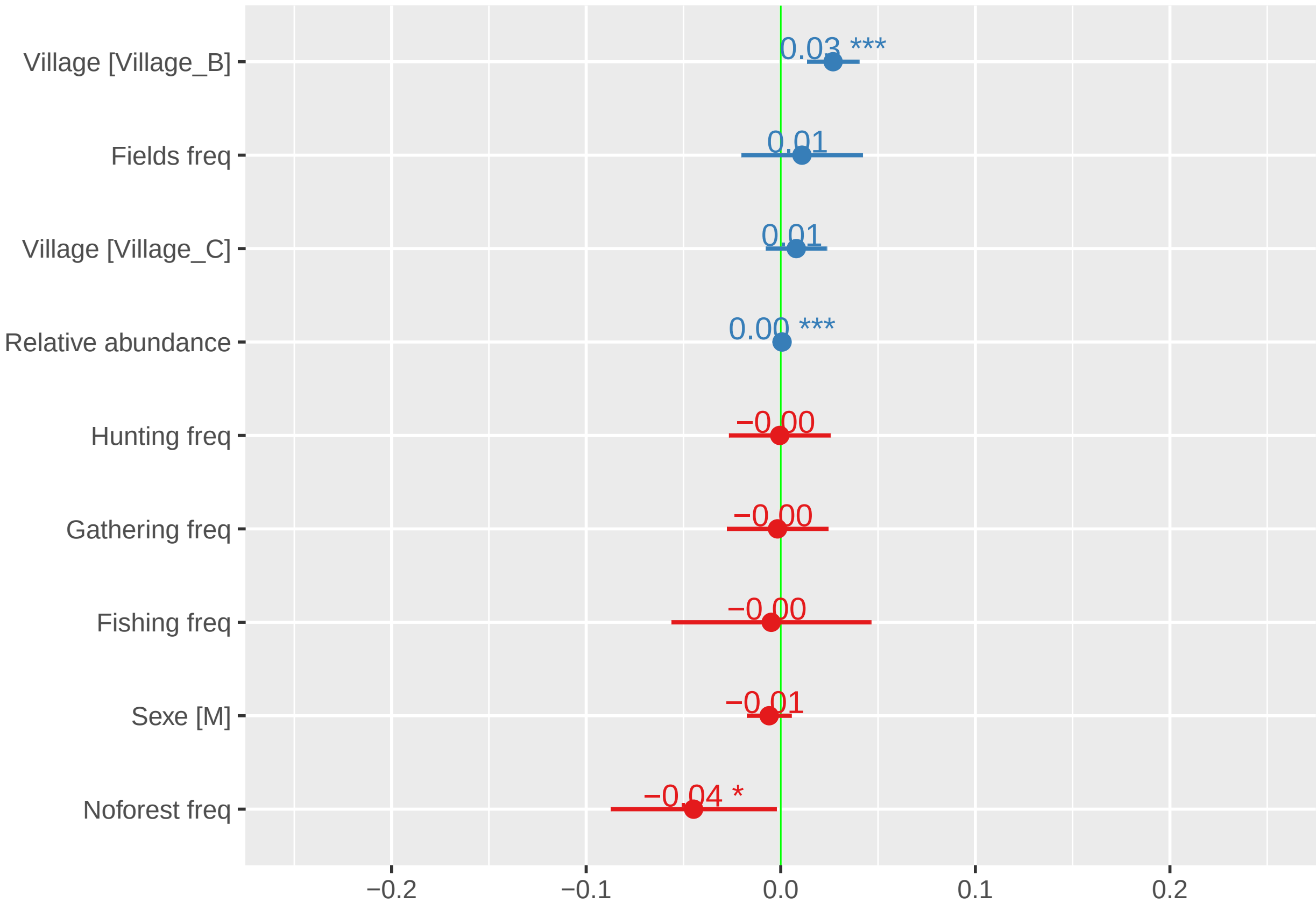

```
#dev.off()
#sjPlot::plot_model(glmm_cluster1_RA_envt, type = "pred")
#sjPlot::plot_model(glmm_cluster1_RA_envt, type = "slope")
```

We also plot the random effects

```
sjPlot::plot_model(glmm_cluster1_RA_envt, auto.label = FALSE, type = "re", # plots random effects
                   vline.color = "green", sort.est = TRUE, show.values = TRUE,
                   # add this to get the effects sorted for random effects
                   grid = FALSE)
```

```
## [[1]]
```

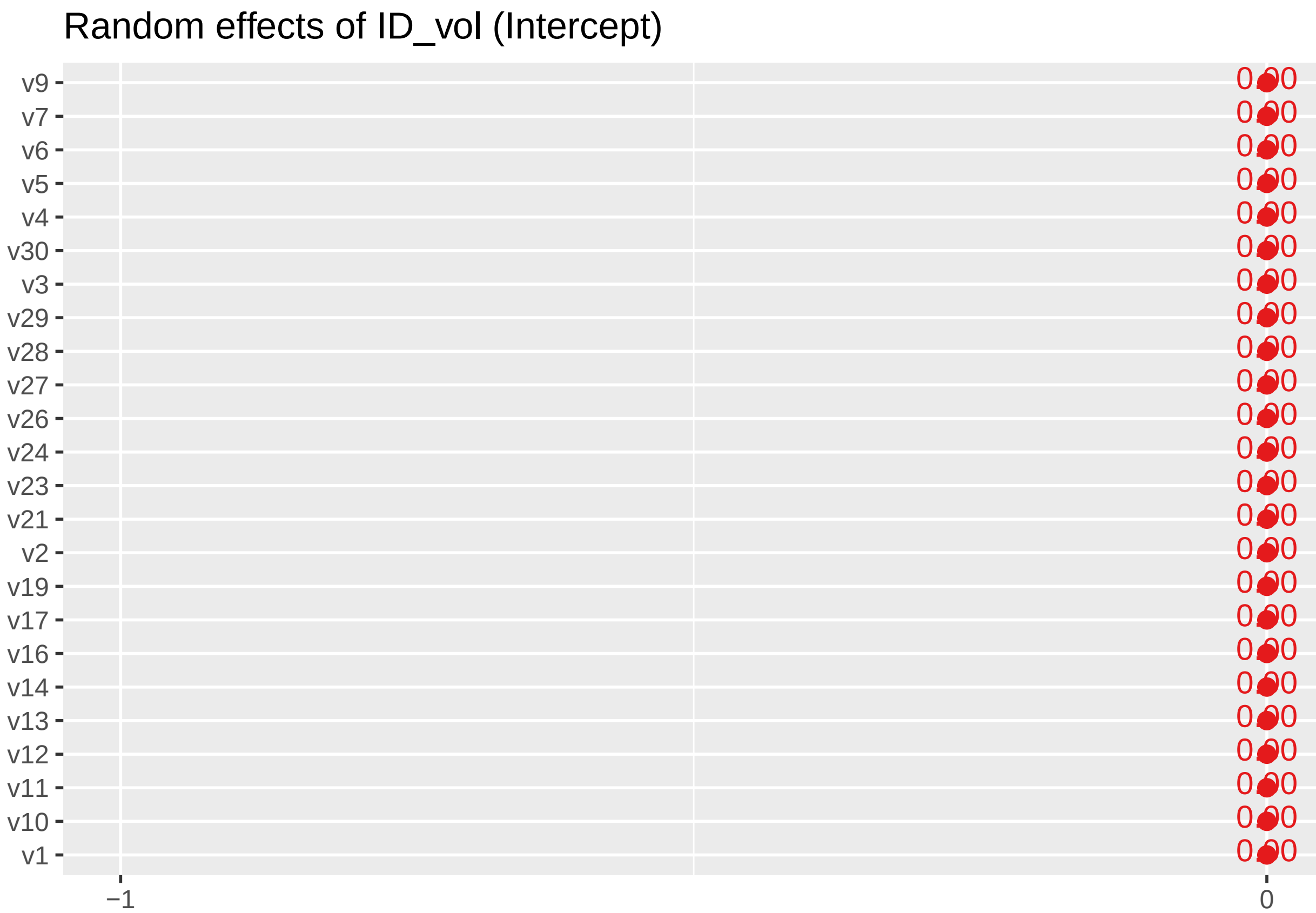


```
##
## [[2]]
```

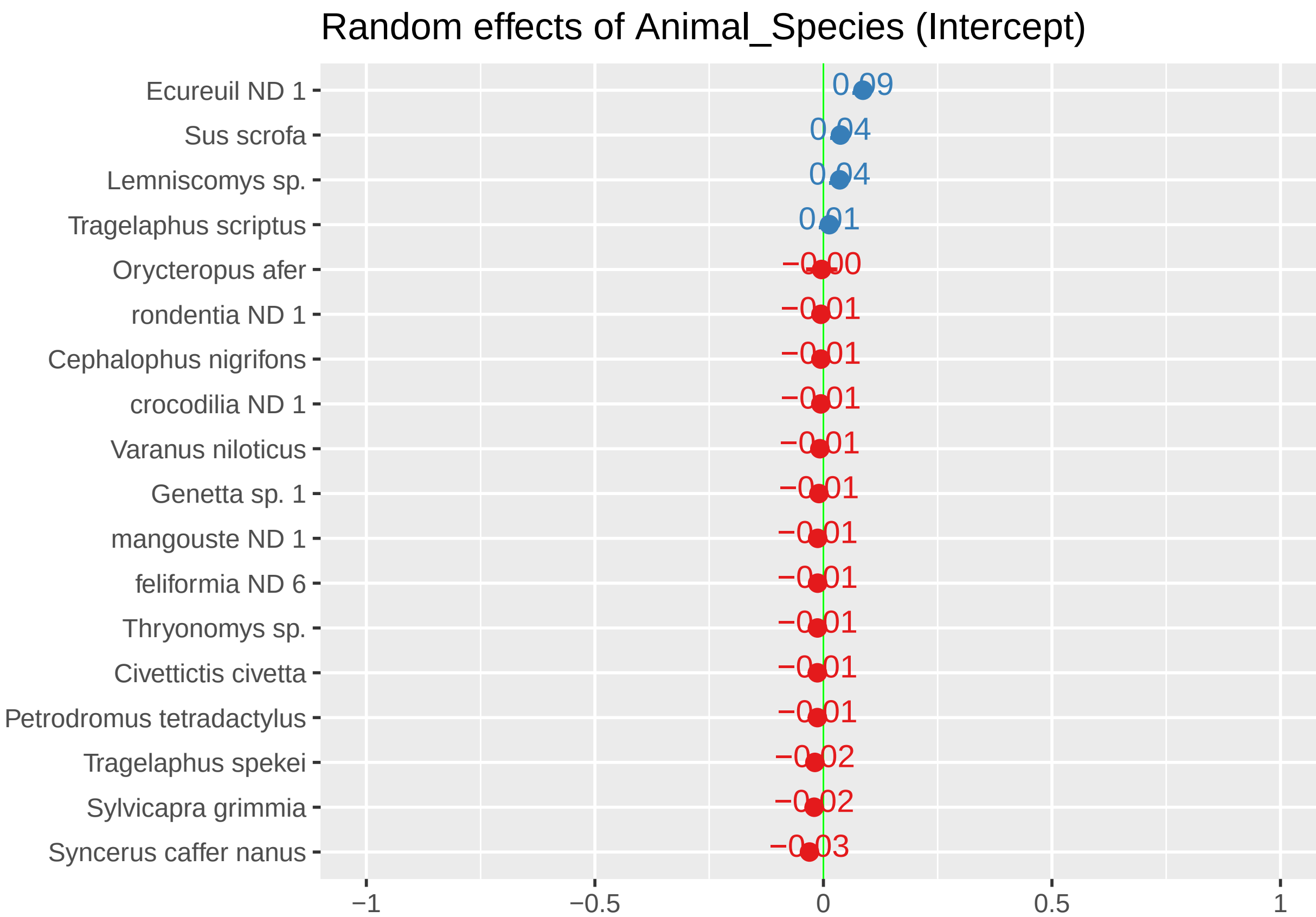

phys

```r
# make the model
glmm_cluster1_RA_phys=lmerTest::lmer(Freq_contact ~ Village + Sexe + Relative_abundance +
                                       Hunting_freq + Fishing_freq + Gathering_freq + Fields_freq +
                                       Noforest_freq + (1|ID_vol) + (1|Animal_Species),
                                     data=data_glmm_RA_cluster1[which(data_glmm_RA_cluster1$ACP_contact=

# model output
summary(glmm_cluster1_RA_phys)
```

```
## Linear mixed model fit by maximum likelihood . t-tests use Satterthwaite's
##   method [lmerModLmerTest]
## Formula: Freq_contact ~ Village + Sexe + Relative_abundance + Hunting_freq +
##     Fishing_freq + Gathering_freq + Fields_freq + Noforest_freq +
##     (1 | ID_vol) + (1 | Animal_Species)
##    Data: data_glmm_RA_cluster1[which(data_glmm_RA_cluster1$ACP_contact ==
##     "physique"), ]
##
##       AIC       BIC    logLik -2*log(L)  df.resid
##   -8474.6   -8407.7    4250.3   -8500.6      1255
##
## Scaled residuals:
##     Min      1Q  Median      3Q     Max
## -1.9313 -0.4749 -0.1842  0.1150  8.6388
##
## Random effects:
```

```
##  Groups          Name        Variance    Std.Dev.
##  ID_vol          (Intercept) 0.000008762 0.002960
##  Animal_Species (Intercept) 0.000007232 0.002689
##  Residual                    0.000067576 0.008220
## Number of obs: 1268, groups:  ID_vol, 24; Animal_Species, 15
##
## Fixed effects:
##                        Estimate  Std. Error          df t value   Pr(>|t|)
## (Intercept)         -0.00472315  0.00663420 25.04927039  -0.712    0.48308
## VillageVillage_B     0.00754100  0.00195920 27.60944950   3.849    0.00064 ***
## VillageVillage_C     0.00520404  0.00199654 34.70464634   2.607    0.01339 *
## SexeM               -0.00045264  0.00200067 24.71801236  -0.226    0.82287
## Relative_abundance   0.00008853  0.00001754 99.40734469   5.046 0.00000204 ***
## Hunting_freq         0.00620747  0.00521090 23.13498414   1.191    0.24563
## Fishing_freq         0.01944070  0.00968963 23.74673140   2.006    0.05634 .
## Gathering_freq      -0.00153525  0.00474847 24.33698580  -0.323    0.74922
## Fields_freq         -0.00335856  0.00561013 24.45172550  -0.599    0.55491
## Noforest_freq        0.01190245  0.00822758 23.61419670   1.447    0.16114
## ---
## Signif. codes:  0 '***' 0.001 '**' 0.01 '*' 0.05 '.' 0.1 ' ' 1
##
## Correlation of Fixed Effects:
##             (Intr) VllV_B VllV_C SexeM  Rltv_b Hntng_ Fshng_ Gthrn_ Flds_f
## VillgVllg_B -0.392
## VillgVllg_C -0.325  0.413
## SexeM       -0.512  0.078  0.043
## Reltv_bndnc -0.081  0.246  0.394  0.000
## Hunting_frq -0.254 -0.040  0.271 -0.282  0.000
## Fishing_frq -0.148  0.340 -0.236  0.187  0.000 -0.496
## Gathrng_frq -0.538  0.102 -0.048  0.175  0.000  0.286 -0.169
## Fields_freq -0.674  0.110  0.266  0.526  0.000 -0.030  0.111 -0.069
## Noforst_frq -0.701  0.395  0.125  0.071  0.000  0.106  0.304  0.372  0.284
```

We also check if the model is better than a null model.

```r
# make the null model
null_cluster1_RA_phys=lmerTest::lmer(Freq_contact ~ (1|ID_vol) + (1|Animal_Species),
                                     data=data_glmm_RA_cluster1[which(data_glmm_RA_cluster1$ACP_contact=
# make the comparison
anova(glmm_cluster1_RA_phys, null_cluster1_RA_phys, test="chisq") ## significatif
```

```
## Data: data_glmm_RA_cluster1[which(data_glmm_RA_cluster1$ACP_contact ==  ...
## Models:
## null_cluster1_RA_phys: Freq_contact ~ (1 | ID_vol) + (1 | Animal_Species)
## glmm_cluster1_RA_phys: Freq_contact ~ Village + Sexe + Relative_abundance + Hunting_freq + Fishing_fr
##                        npar     AIC     BIC logLik -2*log(L)  Chisq Df
## null_cluster1_RA_phys     4 -8457.0 -8436.4 4232.5   -8465.0
## glmm_cluster1_RA_phys    13 -8474.6 -8407.7 4250.3   -8500.6 35.588  9
##                        Pr(>Chisq)
## null_cluster1_RA_phys
## glmm_cluster1_RA_phys 0.00004691 ***
## ---
## Signif. codes:  0 '***' 0.001 '**' 0.01 '*' 0.05 '.' 0.1 ' ' 1
```

We visualize the fixed effects.

```r
#png("plot.png", res=300, height=1100, width=1600)
sjPlot::plot_model(glmm_cluster1_RA_phys, type = "est", auto.label = FALSE,
                   vline.color = "green", sort.est = TRUE, show.values = TRUE) + ylim(-0.25,0.25)
```

```
## Scale for y is already present.
## Adding another scale for y, which will replace the existing scale.
```

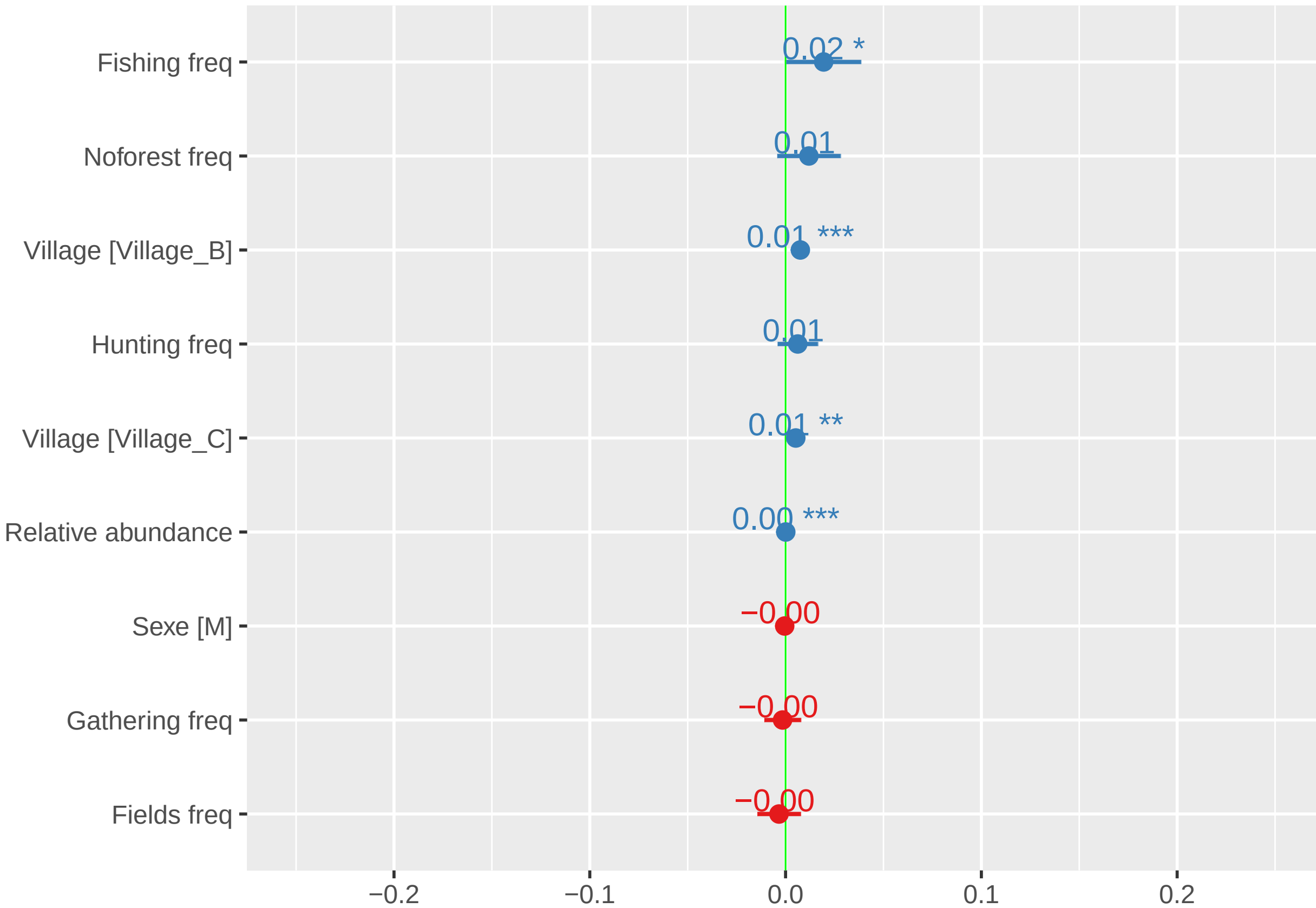


```r
#dev.off()
#sjPlot::plot_model(glmm_cluster1_RA_envt, type = "pred")
#sjPlot::plot_model(glmm_cluster1_RA_envt, type = "slope")
```

We also plot the random effects

```r
sjPlot::plot_model(glmm_cluster1_RA_phys, auto.label = FALSE, type = "re", # plots random effects
                   vline.color = "green", sort.est = TRUE, show.values = TRUE,
                   # add this to get the effects sorted for random effects
                   grid = FALSE)
```

```
## [[1]]
```

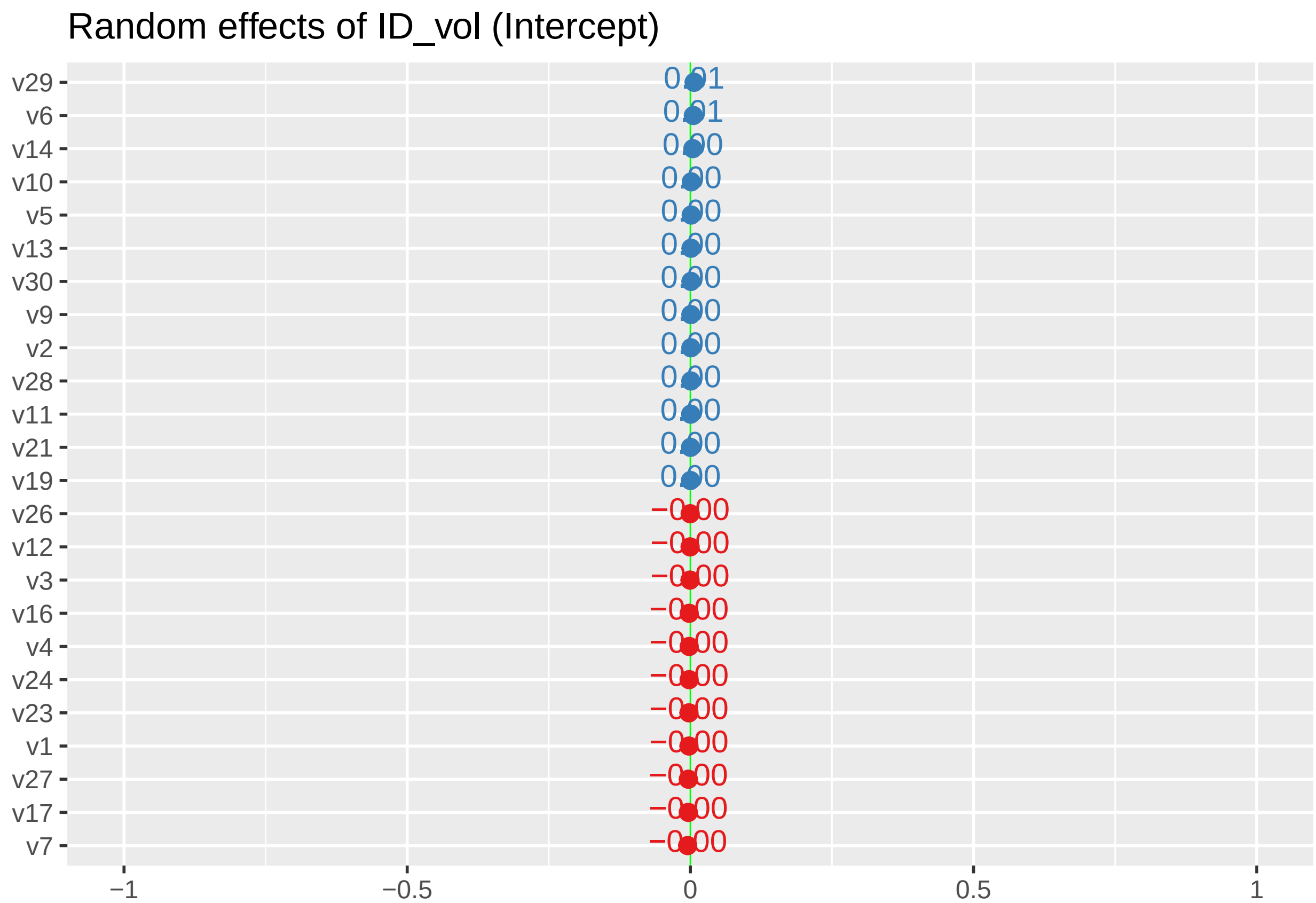
Random effects of ID_vol (Intercept)
v29
v6
v14
v10
v5
v13
v30
v9
v2
v28
v11
v21
v19
v26
v12
v3
v16
v4
v24
v23
v1
v27
v17
v7
0.01
0.01
0.00
0.00
0.00
0.00
0.00
0.00
0.00
0.00
0.00
0.00
0.00
−0.00
−0.00
−0.00
−0.00
−0.00
−0.00
−0.00
−0.00
−0.00
−0.00
−0.00
−1
−0.5
0
0.5
1

```
##
## [[2]]
```

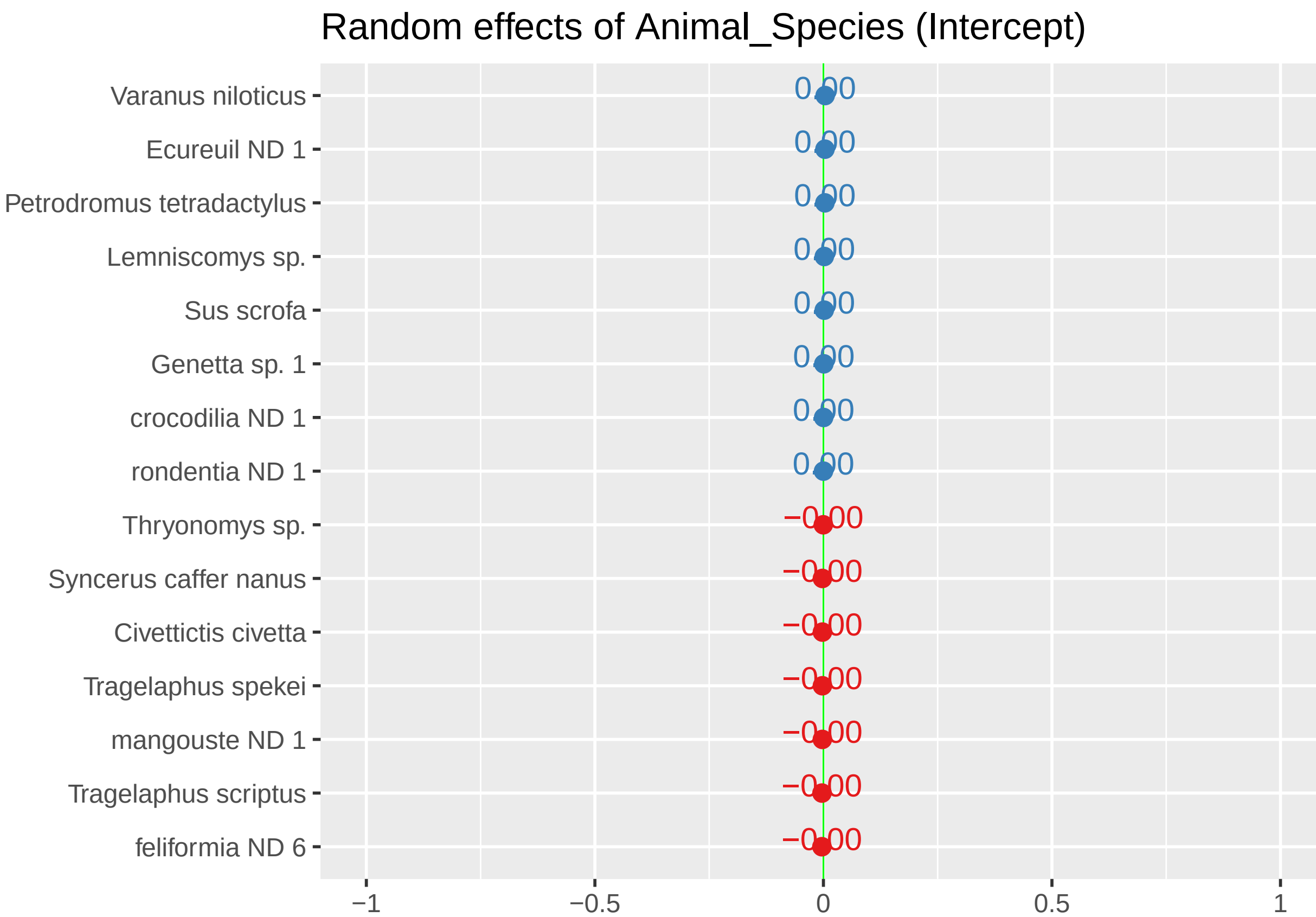

Cluster 2

envt

```
# set the data for cluster 2
data_glmm_RA_cluster2=data_glmm[which(data_glmm$Cluster_CAH=="2" & data_glmm$Relative_abundance!=""),]
# visual check
#str(data_glmm_RA_cluster1)

# make the model
glmm_cluster2_RA_envt=lmerTest::lmer(Freq_contact ~ Village + Sexe + Relative_abundance +
                                       Hunting_freq + Fishing_freq + Gathering_freq +
                                       Fields_freq + Noforest_freq + (1|ID_vol) +
                                       (1|Animal_Species),
                                     data=data_glmm_RA_cluster2[which(data_glmm_RA_cluster2$ACP_contact=

# model output
summary(glmm_cluster2_RA_envt)
```

```
## Linear mixed model fit by maximum likelihood . t-tests use Satterthwaite's
##   method [lmerModLmerTest]
## Formula: Freq_contact ~ Village + Sexe + Relative_abundance + Hunting_freq +
##     Fishing_freq + Gathering_freq + Fields_freq + Noforest_freq +
##     (1 | ID_vol) + (1 | Animal_Species)
##    Data: data_glmm_RA_cluster2[which(data_glmm_RA_cluster2$ACP_contact ==
##     "envt"), ]
##
```

```
##       AIC       BIC    logLik -2*log(L)  df.resid
##   -1692.0   -1632.9     859.0   -1718.0       683
##
## Scaled residuals:
##     Min      1Q  Median      3Q     Max
## -1.8270 -0.4918 -0.1788  0.1442  7.4910
##
## Random effects:
##  Groups         Name        Variance  Std.Dev.
##  ID_vol         (Intercept) 0.0003507 0.01873
##  Animal_Species (Intercept) 0.0003581 0.01892
##  Residual                   0.0046905 0.06849
## Number of obs: 696, groups:  ID_vol, 24; Animal_Species, 5
##
## Fixed effects:
##                        Estimate  Std. Error          df t value Pr(>|t|)
## (Intercept)           0.0165128   0.0473845  25.2342690   0.348   0.7304
## VillageVillage_B      0.0029083   0.0134347  23.9481781   0.216   0.8305
## VillageVillage_C     -0.0047614   0.0127775  23.4018852  -0.373   0.7128
## SexeM                 0.0051702   0.0140417  23.3977580   0.368   0.7160
## Relative_abundance    0.0003219   0.0001256 198.6776455   2.564   0.0111 *
## Hunting_freq         -0.0250249   0.0371647  23.3977580  -0.673   0.5073
## Fishing_freq          0.0333127   0.0686553  23.3977579   0.485   0.6320
## Gathering_freq        0.0273101   0.0334415  23.3977580   0.817   0.4224
## Fields_freq          -0.0025627   0.0394676  23.3977580  -0.065   0.9488
## Noforest_freq         0.0067032   0.0583715  23.3977580   0.115   0.9096
## ---
## Signif. codes:  0 '***' 0.001 '**' 0.01 '*' 0.05 '.' 0.1 ' ' 1
##
## Correlation of Fixed Effects:
##             (Intr) VllV_B VllV_C SexeM  Rltv_b Hntng_ Fshng_ Gthrn_ Flds_f
## VillgVllg_B -0.396
## VillgVllg_C -0.326  0.356
## SexeM       -0.498  0.077  0.050
## Reltv_bndnc -0.103  0.108  0.009  0.000
## Hunting_frq -0.251 -0.050  0.304 -0.277  0.000
## Fishing_frq -0.144  0.352 -0.264  0.177  0.000 -0.505
## Gathrng_frq -0.533  0.106 -0.051  0.169  0.000  0.283 -0.170
## Fields_freq -0.666  0.114  0.294  0.523  0.000 -0.019  0.112 -0.067
## Noforst_frq -0.689  0.409  0.131  0.068  0.000  0.087  0.311  0.376  0.280
```

We also check if the model is better than a null model.

```
# make the null model
null_cluster2_RA_envt=lmerTest::lmer(Freq_contact ~ (1|ID_vol) + (1|Animal_Species),
                                     data=data_glmm_RA_cluster2[which(data_glmm_RA_cluster2$ACP_contact=

# make the comparison
anova(glmm_cluster2_RA_envt, null_cluster2_RA_envt, test="chisq")
```

```
## Data: data_glmm_RA_cluster2[which(data_glmm_RA_cluster2$ACP_contact ==  ...
## Models:
## null_cluster2_RA_envt: Freq_contact ~ (1 | ID_vol) + (1 | Animal_Species)
## glmm_cluster2_RA_envt: Freq_contact ~ Village + Sexe + Relative_abundance + Hunting_freq + Fishing_f
##                       npar     AIC     BIC logLik -2*log(L)  Chisq Df
```

```
## null_cluster2_RA_envt    4 -1702.6 -1684.5 855.32   -1710.6
## glmm_cluster2_RA_envt   13 -1692.0 -1632.9 858.99   -1718.0 7.3463  9
##                        Pr(>Chisq)
## null_cluster2_RA_envt
## glmm_cluster2_RA_envt     0.6011
```

We visualize the fixed effects.

```r
#png("plot.png", res=300, height=1100, width=1600)
sjPlot::plot_model(glmm_cluster2_RA_envt, type = "est", auto.label = FALSE,
                   vline.color = "green", sort.est = TRUE, show.values = TRUE) + ylim(-0.25,0.25)
```

```
## Scale for y is already present.
## Adding another scale for y, which will replace the existing scale.
```

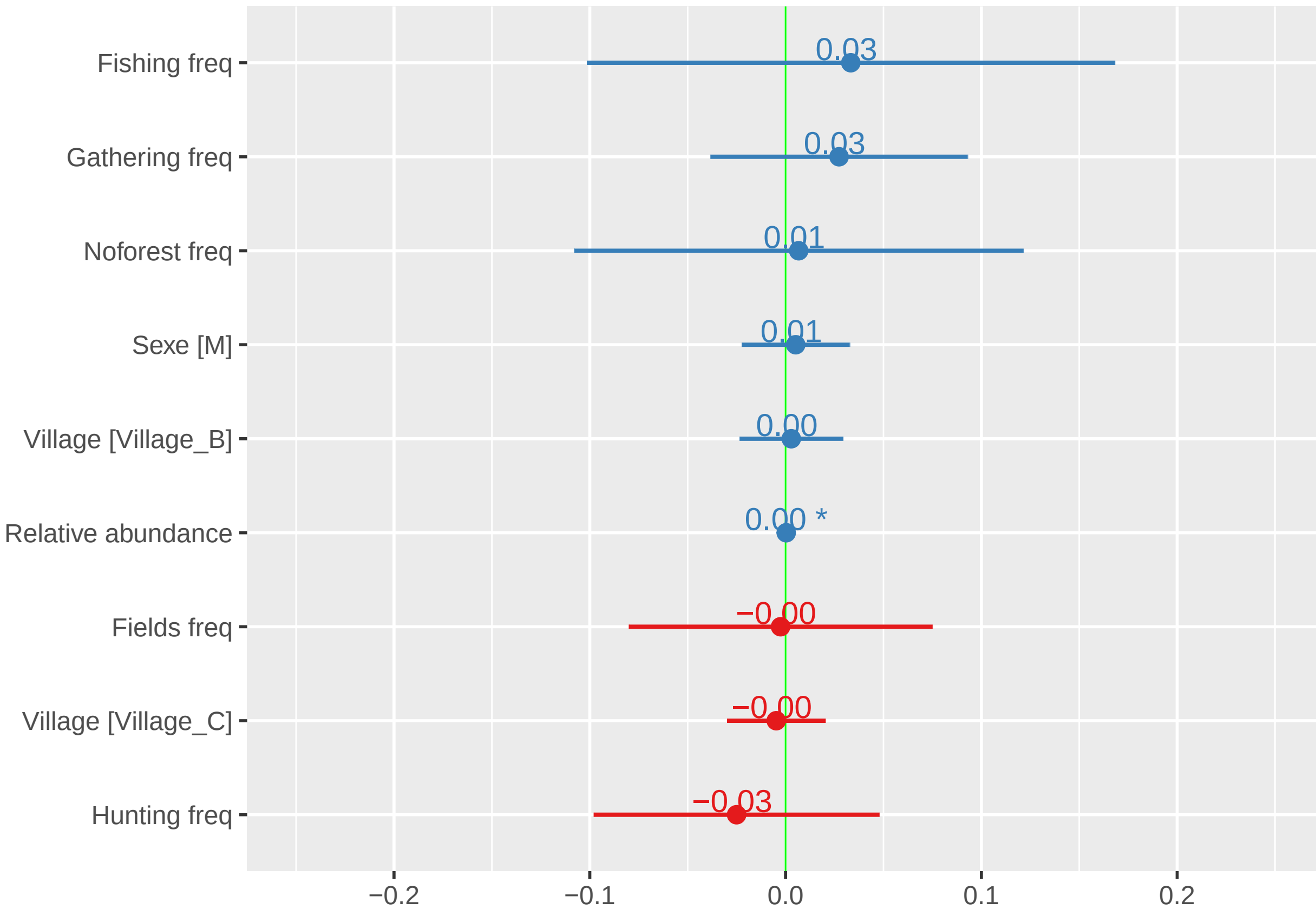


```r
#dev.off()
#sjPlot::plot_model(glmm_cluster1_RA_envt, type = "pred")
#sjPlot::plot_model(glmm_cluster1_RA_envt, type = "slope")
```

We also plot the random effects

```r
sjPlot::plot_model(glmm_cluster2_RA_envt, auto.label = FALSE, type = "re", # plots random effects
                   vline.color = "green", sort.est = TRUE, show.values = TRUE,
                   # add this to get the effects sorted for random effects
                   grid = FALSE)
```

```
## [[1]]
```

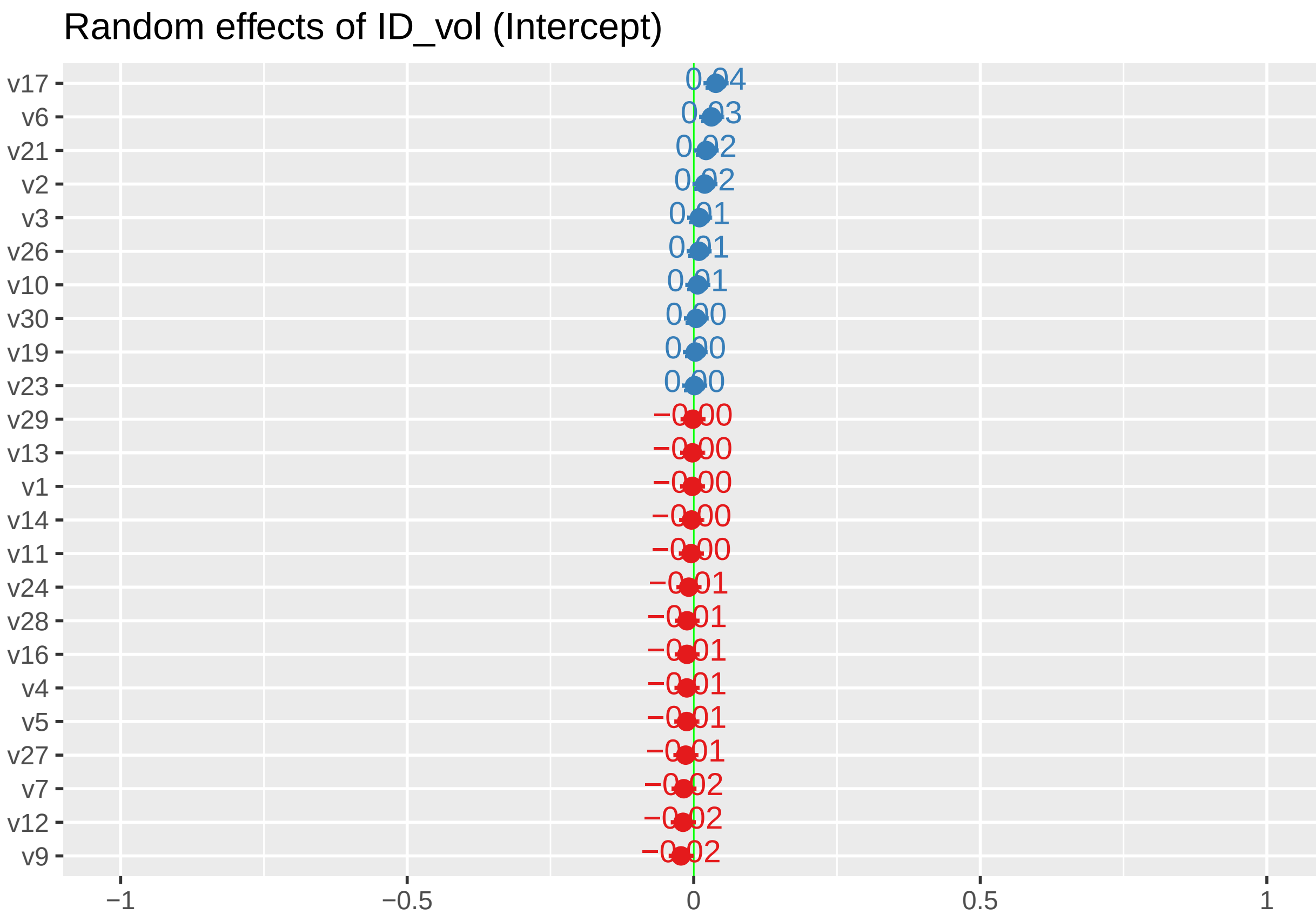
Random effects of ID_vol (Intercept)
v17
v6
v21
v2
v3
v26
v10
v30
v19
v23
v29
v13
v1
v14
v11
v24
v28
v16
v4
v5
v27
v7
v12
v9
0.04
0.03
0.02
0.02
0.01
0.01
0.01
0.00
0.00
0.00
−0.00
−0.00
−0.00
−0.00
−0.00
−0.01
−0.01
−0.01
−0.01
−0.01
−0.01
−0.02
−0.02
−0.02
−1
−0.5
0
0.5
1

```
##
## [[2]]
```

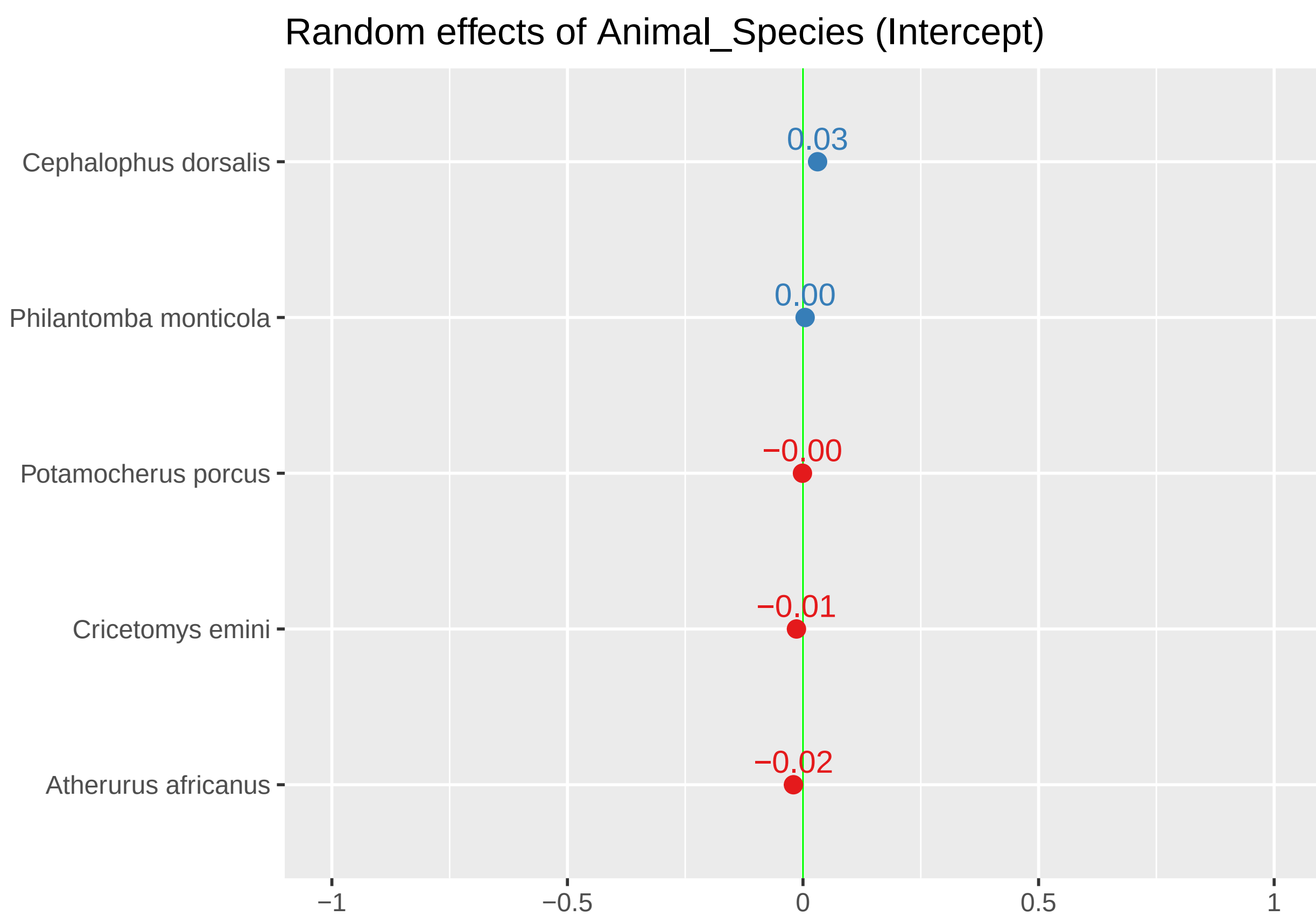

phys

```
# make the model
glmm_cluster2_RA_phys=lmerTest::lmer(Freq_contact ~ Village + Sexe + Relative_abundance +
                                       Hunting_freq + Fishing_freq + Gathering_freq +
                                       Fields_freq + Noforest_freq + (1|ID_vol) +
                                       (1|Animal_Species),
                                     data=data_glmm_RA_cluster2[which(data_glmm_RA_cluster2$ACP_contact=

# model output
summary(glmm_cluster2_RA_phys)
```

```
## Linear mixed model fit by maximum likelihood . t-tests use Satterthwaite's
##   method [lmerModLmerTest]
## Formula: Freq_contact ~ Village + Sexe + Relative_abundance + Hunting_freq +
##     Fishing_freq + Gathering_freq + Fields_freq + Noforest_freq +
##     (1 | ID_vol) + (1 | Animal_Species)
##    Data: data_glmm_RA_cluster2[which(data_glmm_RA_cluster2$ACP_contact ==
##     "physique"), ]
##
##       AIC       BIC    logLik -2*log(L)  df.resid
##   -4685.7   -4622.5    2355.9   -4711.7       947
##
## Scaled residuals:
##     Min      1Q  Median      3Q     Max
## -2.3472 -0.5956 -0.2516  0.3591  4.8136
##
```

```
## Random effects:
##  Groups         Name        Variance   Std.Dev.
##  ID_vol         (Intercept) 0.00003399 0.005830
##  Animal_Species (Intercept) 0.00001269 0.003562
##  Residual                   0.00041332 0.020330
## Number of obs: 960, groups:  ID_vol, 24; Animal_Species, 5
##
## Fixed effects:
##                        Estimate  Std. Error          df t value Pr(>|t|)
## (Intercept)         -0.00102882  0.01378032 24.57063179  -0.075   0.9411
## VillageVillage_B     0.00986152  0.00394378 24.02827039   2.501   0.0196 *
## VillageVillage_C     0.00634442  0.00375761 23.65163310   1.688   0.1045
## SexeM               -0.00473885  0.00412935 23.64685759  -1.148   0.2626
## Relative_abundance   0.00007885  0.00003080 98.17144307   2.560   0.0120 *
## Hunting_freq         0.02637516  0.01092933 23.64685764   2.413   0.0239 *
## Fishing_freq         0.03799497  0.02019002 23.64685759   1.882   0.0722 .
## Gathering_freq       0.01041475  0.00983441 23.64685763   1.059   0.3003
## Fields_freq         -0.02438924  0.01160657 23.64685756  -2.101   0.0465 *
## Noforest_freq        0.03033073  0.01716578 23.64685751   1.767   0.0901 .
## ---
## Signif. codes:  0 '***' 0.001 '**' 0.01 '*' 0.05 '.' 0.1 ' ' 1
##
## Correlation of Fixed Effects:
##             (Intr) VllV_B VllV_C SexeM  Rltv_b Hntng_ Fshng_ Gthrn_ Flds_f
## VillgVllg_B -0.397
## VillgVllg_C -0.330  0.357
## SexeM       -0.504  0.077  0.050
## Reltv_bndnc -0.087  0.090  0.010  0.000
## Hunting_frq -0.253 -0.050  0.304 -0.277  0.000
## Fishing_frq -0.145  0.353 -0.264  0.177  0.000 -0.505
## Gathrng_frq -0.539  0.107 -0.051  0.169  0.000  0.283 -0.170
## Fields_freq -0.673  0.114  0.294  0.523  0.000 -0.019  0.112 -0.067
## Noforst_frq -0.697  0.410  0.131  0.068  0.000  0.087  0.311  0.376  0.280
```

We also check if the model is better than a null model.

```
# make the null model
null_cluster2_RA_phys=lmerTest::lmer(Freq_contact ~ (1|ID_vol) + (1|Animal_Species),
                                     data=data_glmm_RA_cluster2[which(data_glmm_RA_cluster2$ACP_contact=

# make the comparison
anova(glmm_cluster2_RA_phys, null_cluster2_RA_phys, test="chisq") ## significatif
```

```
## Data: data_glmm_RA_cluster2[which(data_glmm_RA_cluster2$ACP_contact ==  ...
## Models:
## null_cluster2_RA_phys: Freq_contact ~ (1 | ID_vol) + (1 | Animal_Species)
## glmm_cluster2_RA_phys: Freq_contact ~ Village + Sexe + Relative_abundance + Hunting_freq + Fishing_f
##                       npar     AIC     BIC logLik -2*log(L) Chisq Df Pr(>Chisq)
## null_cluster2_RA_phys    4 -4676.9 -4657.5 2342.5   -4684.9
## glmm_cluster2_RA_phys   13 -4685.7 -4622.5 2355.9   -4711.7  26.8  9   0.001509
##
## null_cluster2_RA_phys
## glmm_cluster2_RA_phys **
## ---
## Signif. codes:  0 '***' 0.001 '**' 0.01 '*' 0.05 '.' 0.1 ' ' 1
```

We visualize the fixed effects.

```
#png("plot.png", res=300, height=1100, width=1600)
sjPlot::plot_model(glmm_cluster2_RA_phys, type = "est", auto.label = FALSE,
                   vline.color = "green", sort.est = TRUE, show.values = TRUE) + ylim(-0.25,0.25)
```

```
## Scale for y is already present.
## Adding another scale for y, which will replace the existing scale.
```

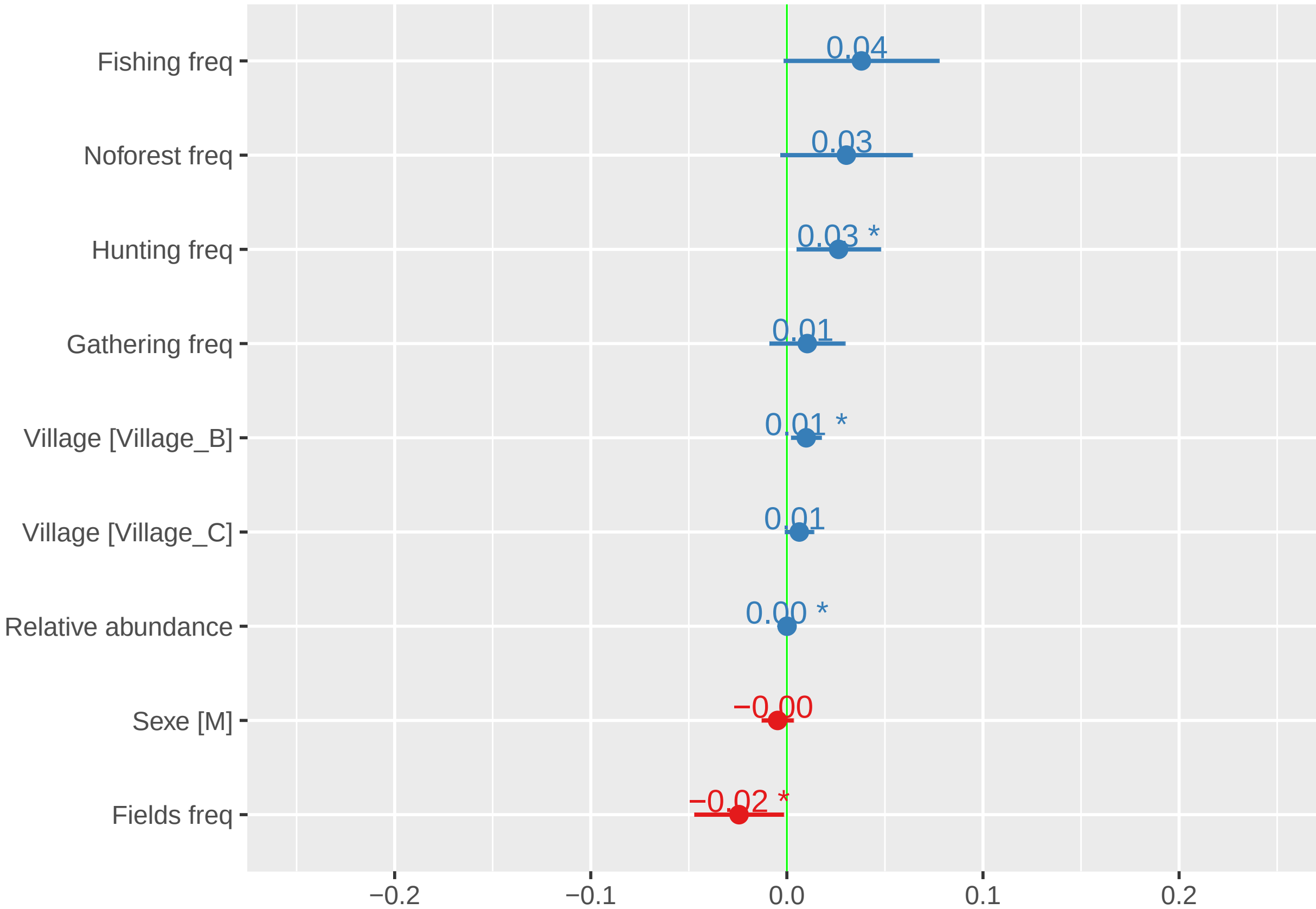


```
#dev.off()
#sjPlot::plot_model(glmm_cluster1_RA_envt, type = "pred")
#sjPlot::plot_model(glmm_cluster1_RA_envt, type = "slope")
```

We also plot the random effects

```
sjPlot::plot_model(glmm_cluster2_RA_phys, auto.label = FALSE, type = "re", # plots random effects
                   vline.color = "green", sort.est = TRUE, show.values = TRUE,
                   # add this to get the effects sorted for random effects
                   grid = FALSE)
```

```
## [[1]]
```

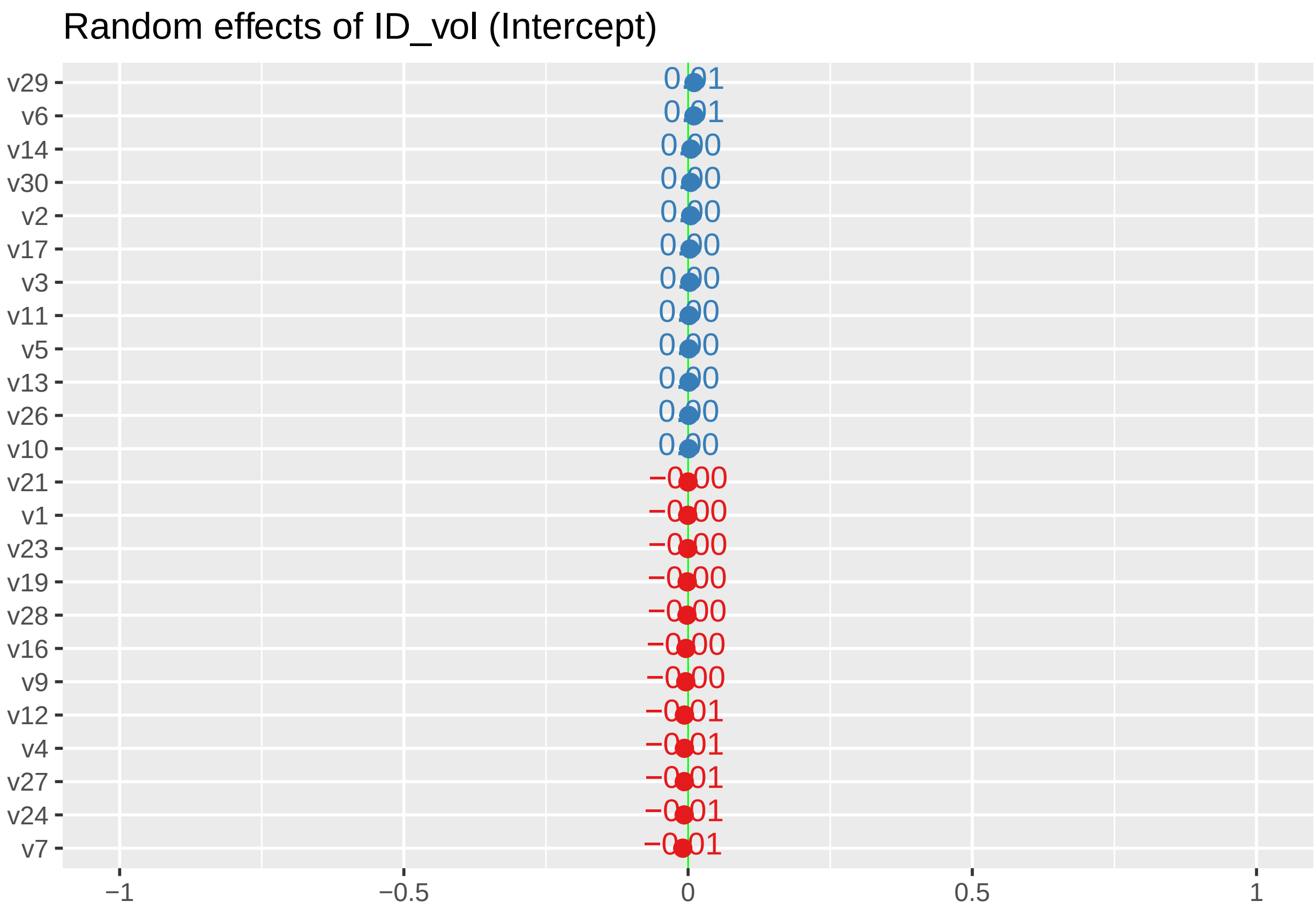
Random effects of ID_vol (Intercept)
v29
v6
v14
v30
v2
v17
v3
v11
v5
v13
v26
v10
v21
v1
v23
v19
v28
v16
v9
v12
v4
v27
v24
v7
0.01
0.01
0.00
0.00
0.00
0.00
0.00
0.00
0.00
0.00
0.00
0.00
−0.00
−0.00
−0.00
−0.00
−0.00
−0.00
−0.00
−0.01
−0.01
−0.01
−0.01
−0.01
−1
−0.5
0
0.5
1

```
##
## [[2]]
```

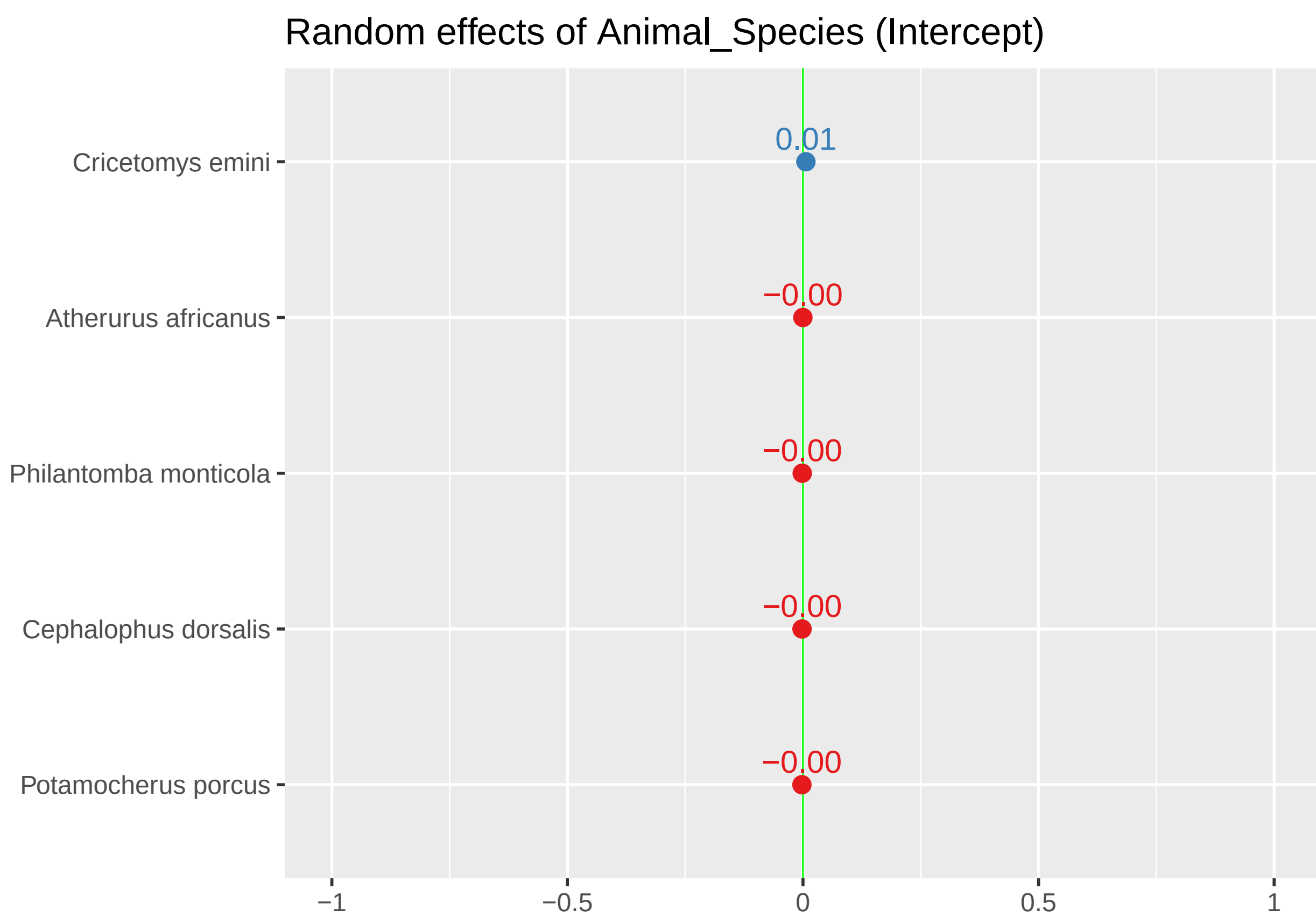

Cluster 3

envt

```
# set the data for cluster 2
data_glmm_RA_cluster3=data_glmm[which(data_glmm$Cluster_CAH=="3" & data_glmm$Relative_abundance!=""),]
# visual check
#str(data_glmm_RA_cluster1)

# make the model
glmm_cluster3_RA_envt=lmerTest::lmer(Freq_contact ~ Village + Sexe + Relative_abundance +
                                       Hunting_freq + Fishing_freq + Gathering_freq +
                                       Fields_freq + Noforest_freq + (1|ID_vol) +
                                       (1|Animal_Species),
                                     data=data_glmm_RA_cluster3[which(data_glmm_RA_cluster3$ACP_contact=

# model output
summary(glmm_cluster3_RA_envt)
```

```
## Linear mixed model fit by maximum likelihood . t-tests use Satterthwaite's
##   method [lmerModLmerTest]
## Formula: Freq_contact ~ Village + Sexe + Relative_abundance + Hunting_freq +
##     Fishing_freq + Gathering_freq + Fields_freq + Noforest_freq +
##     (1 | ID_vol) + (1 | Animal_Species)
##    Data: data_glmm_RA_cluster3[which(data_glmm_RA_cluster3$ACP_contact ==
##     "envt"), ]
##
```

```
##       AIC       BIC    logLik -2*log(L)  df.resid
##     335.3     397.8    -154.6     309.3       893
##
## Scaled residuals:
##     Min      1Q  Median      3Q     Max
## -2.5011 -0.6454  0.2240  0.7554  1.9453
##
## Random effects:
##  Groups         Name        Variance Std.Dev.
##  ID_vol         (Intercept) 0.005014 0.07081
##  Animal_Species (Intercept) 0.014698 0.12123
##  Residual                   0.077729 0.27880
## Number of obs: 906, groups:  ID_vol, 24; Animal_Species, 8
##
## Fixed effects:
##                       Estimate  Std. Error         df t value       Pr(>|t|)
## (Intercept)          0.1839127   0.1802393  26.2687128   1.020         0.3169
## VillageVillage_B    -0.0001292   0.0501888  23.3673687  -0.003         0.9980
## VillageVillage_C    -0.1063177   0.0492369  25.8334304  -2.159         0.0403
## SexeM               -0.0121151   0.0527529  23.3237175  -0.230         0.8204
## Relative_abundance   0.0017075   0.0002683 555.1774066   6.365 0.000000000409
## Hunting_freq         0.1857829   0.1376993  22.0053836   1.349         0.1910
## Fishing_freq         0.3051088   0.2571574  22.9708984   1.186         0.2476
## Gathering_freq       0.0447284   0.1257486  23.3372491   0.356         0.7253
## Fields_freq          0.0237061   0.1485991  23.4689505   0.160         0.8746
## Noforest_freq        0.1669718   0.2180839  22.7235593   0.766         0.4518
##
## (Intercept)
## VillageVillage_B
## VillageVillage_C   *
## SexeM
## Relative_abundance ***
## Hunting_freq
## Fishing_freq
## Gathering_freq
## Fields_freq
## Noforest_freq
## ---
## Signif. codes:  0 '***' 0.001 '**' 0.01 '*' 0.05 '.' 0.1 ' ' 1
##
## Correlation of Fixed Effects:
##             (Intr) VllV_B VllV_C SexeM  Rltv_b Hntng_ Fshng_ Gthrn_ Flds_f
## VillgVllg_B -0.390
## VillgVllg_C -0.325  0.351
## SexeM       -0.498  0.084  0.047
## Reltv_bndnc -0.107  0.131  0.184  0.000
## Hunting_frq -0.252 -0.039  0.289 -0.279  0.000
## Fishing_frq -0.147  0.352 -0.253  0.193  0.000 -0.494
## Gathrng_frq -0.522  0.106 -0.052  0.167  0.000  0.294 -0.169
## Fields_freq -0.654  0.114  0.288  0.526  0.000 -0.031  0.112 -0.076
## Noforst_frq -0.686  0.409  0.136  0.074  0.000  0.112  0.303  0.373  0.284
```

We also check if the model is better than a null model.

```r
# make the null model
null_cluster3_RA_envt=lmerTest::lmer(Freq_contact ~ (1|ID_vol) + (1|Animal_Species),
                                     data=data_glmm_RA_cluster3[which(data_glmm_RA_cluster3$ACP_contact=

# make the comparison
anova(glmm_cluster3_RA_envt, null_cluster3_RA_envt, test="chisq")
```

```
## Data: data_glmm_RA_cluster3[which(data_glmm_RA_cluster3$ACP_contact ==  ...
## Models:
## null_cluster3_RA_envt: Freq_contact ~ (1 | ID_vol) + (1 | Animal_Species)
## glmm_cluster3_RA_envt: Freq_contact ~ Village + Sexe + Relative_abundance + Hunting_freq + Fishing_f
##                       npar    AIC    BIC  logLik -2*log(L) Chisq Df
## null_cluster3_RA_envt    4 371.83 391.07 -181.91    363.83
## glmm_cluster3_RA_envt   13 335.25 397.77 -154.62    309.25 54.58  9
##                          Pr(>Chisq)
## null_cluster3_RA_envt
## glmm_cluster3_RA_envt 0.00000001464 ***
## ---
## Signif. codes:  0 '***' 0.001 '**' 0.01 '*' 0.05 '.' 0.1 ' ' 1
```

We visualize the fixed effects.

```r
#png("plot.png", res=300, height=1100, width=1600)
sjPlot::plot_model(glmm_cluster3_RA_envt, type = "est", auto.label = FALSE,
                   vline.color = "green", sort.est = TRUE, show.values = TRUE) + ylim(-0.25,0.25)
```

```
## Scale for y is already present.
## Adding another scale for y, which will replace the existing scale.

## Warning: Removed 1 row containing missing values or values outside the scale range
## (`geom_point()`).

## Warning: Removed 1 row containing missing values or values outside the scale range
## (`geom_text()`).
```

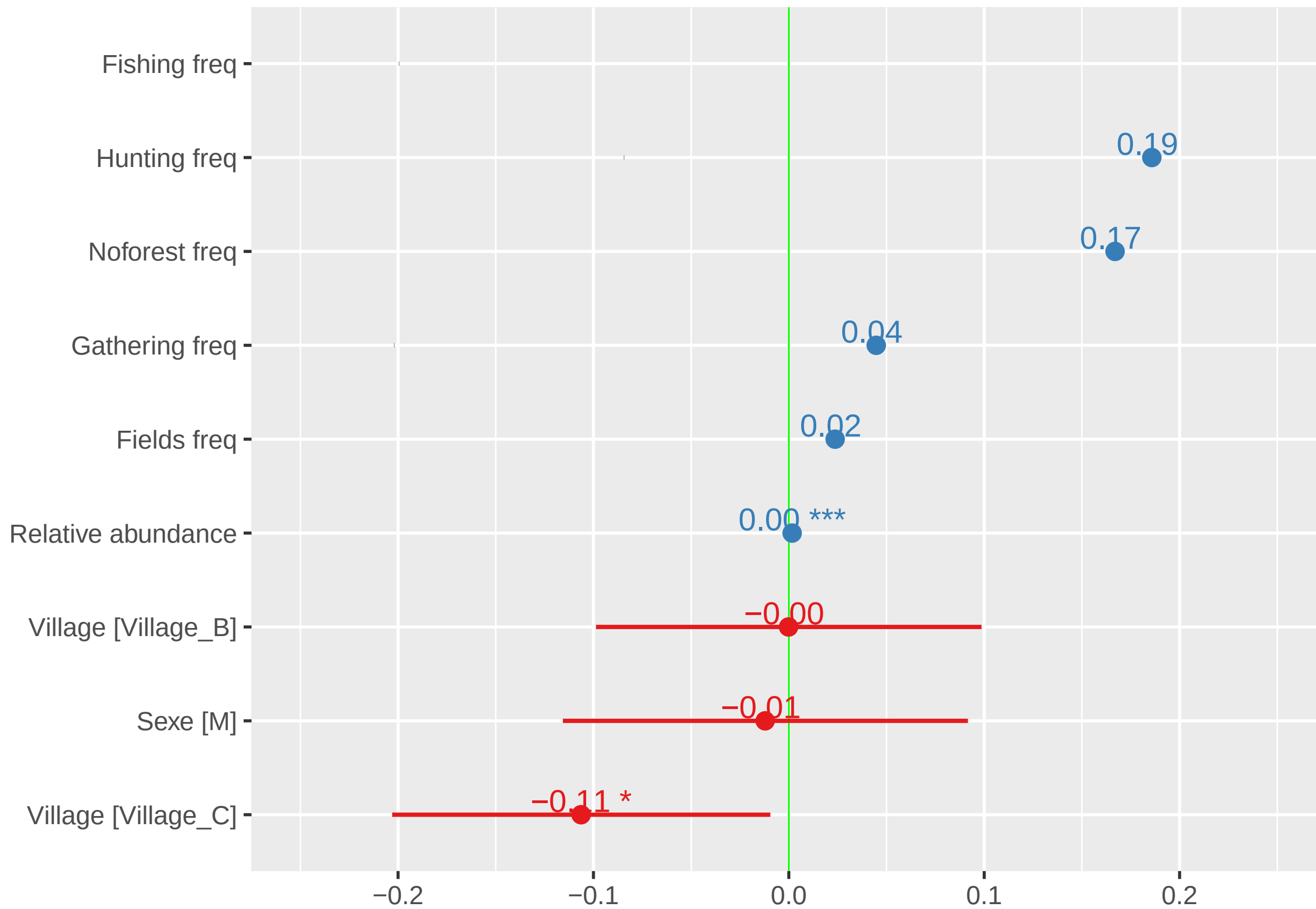


```
#dev.off()
#sjPlot::plot_model(glmm_cluster1_RA_envt, type = "pred")
#sjPlot::plot_model(glmm_cluster1_RA_envt, type = "slope")
```

We also plot the random effects

```
sjPlot::plot_model(glmm_cluster3_RA_envt, auto.label = FALSE, type = "re", # plots random effects
                   vline.color = "green", sort.est = TRUE, show.values = TRUE,
                   # add this to get the effects sorted for random effects
                   grid = FALSE)
```

```
## [[1]]
```

Random effects of ID_vol (Intercept)

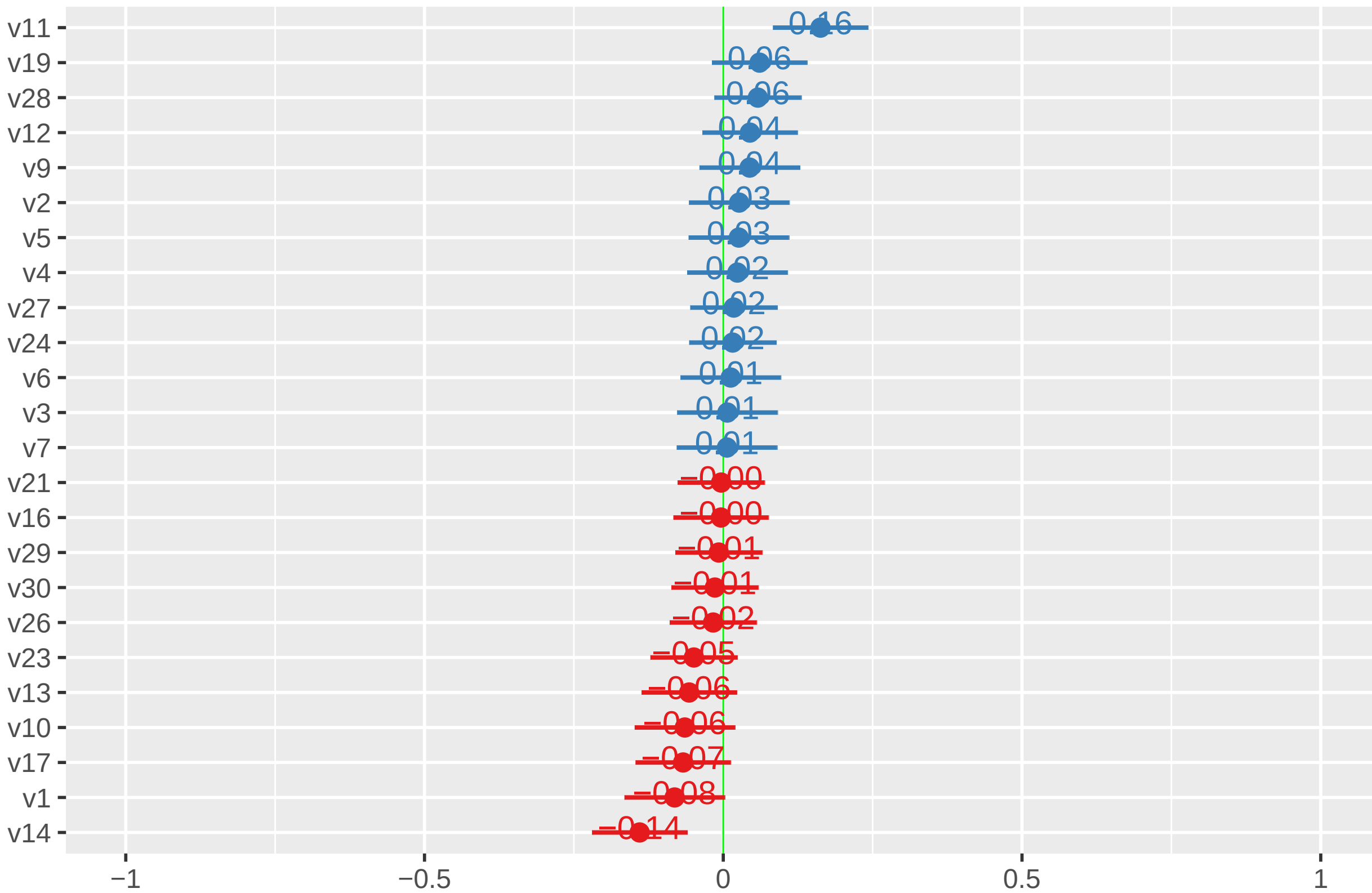


```
##
## [[2]]
```

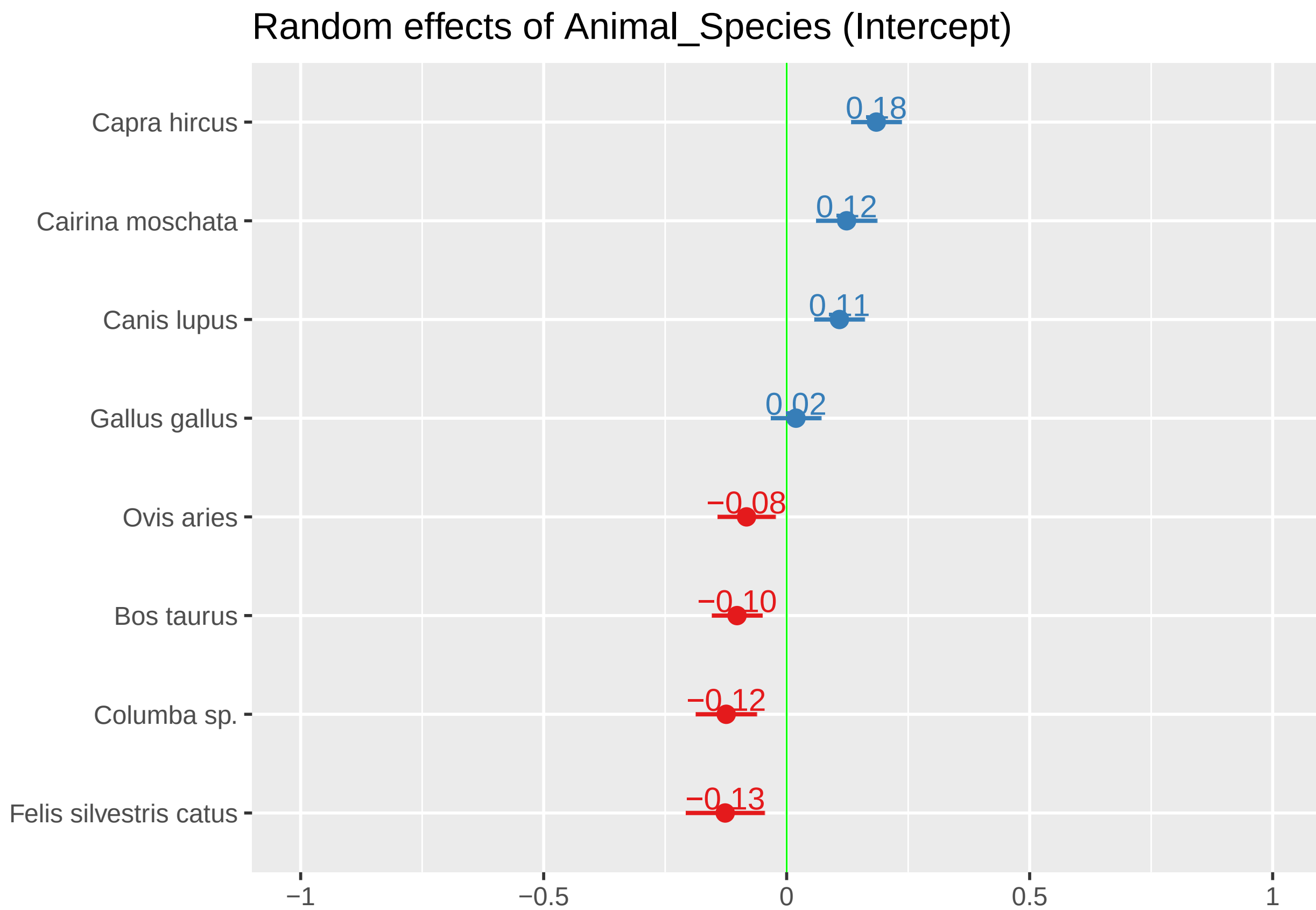

phys

```
# make the model
glmm_cluster3_RA_phys=lmerTest::lmer(Freq_contact ~ Village + Sexe + Relative_abundance +
                                      Hunting_freq + Fishing_freq + Gathering_freq +
                                      Fields_freq + Noforest_freq + (1|ID_vol) +
                                      (1|Animal_Species),
                                    data=data_glmm_RA_cluster3[which(data_glmm_RA_cluster3$ACP_contact=

# model output
summary(glmm_cluster3_RA_phys)
```

```
## Linear mixed model fit by maximum likelihood . t-tests use Satterthwaite's
##   method [lmerModLmerTest]
## Formula: Freq_contact ~ Village + Sexe + Relative_abundance + Hunting_freq +
##     Fishing_freq + Gathering_freq + Fields_freq + Noforest_freq +
##     (1 | ID_vol) + (1 | Animal_Species)
##    Data: data_glmm_RA_cluster3[which(data_glmm_RA_cluster3$ACP_contact ==
##     "physique"), ]
##
##       AIC       BIC    logLik -2*log(L)  df.resid
##   -6177.1   -6113.1    3101.5   -6203.1      1004
##
## Scaled residuals:
##     Min      1Q  Median      3Q     Max
## -2.4162 -0.3589 -0.0196  0.1828 11.0749
##
```

```
## Random effects:
##  Groups         Name        Variance   Std.Dev.
##  ID_vol         (Intercept) 0.00003062 0.005534
##  Animal_Species (Intercept) 0.00001138 0.003373
##  Residual                   0.00012193 0.011042
## Number of obs: 1017, groups:  ID_vol, 24; Animal_Species, 8
##
## Fixed effects:
##                         Estimate    Std. Error           df t value Pr(>|t|)
## (Intercept)          -0.004942554   0.011965607  24.932884407  -0.413    0.683
## VillageVillage_B      0.000428011   0.003420154  24.209767620   0.125    0.901
## VillageVillage_C      0.001298672   0.003292677  24.829996107   0.394    0.697
## SexeM                -0.001059163   0.003597540  24.261289308  -0.294    0.771
## Relative_abundance    0.000005732   0.000009656 381.438981131   0.594    0.553
## Hunting_freq          0.012463537   0.009496715  24.003683710   1.312    0.202
## Fishing_freq          0.006999805   0.017592785  24.273577133   0.398    0.694
## Gathering_freq        0.002800601   0.008574599  24.333774329   0.327    0.747
## Fields_freq          -0.002178175   0.010122587  24.361460436  -0.215    0.831
## Noforest_freq         0.017572619   0.014947957  24.210270693   1.176    0.251
##
## Correlation of Fixed Effects:
##             (Intr) VllV_B VllV_C SexeM  Rltv_b Hntng_ Fshng_ Gthrn_ Flds_f
## VillgVllg_B -0.395
## VillgVllg_C -0.332  0.354
## SexeM       -0.507  0.080  0.049
## Reltv_bndnc -0.059  0.065  0.087  0.000
## Hunting_frq -0.256 -0.048  0.301 -0.277  0.000
## Fishing_frq -0.147  0.354 -0.262  0.181  0.000 -0.503
## Gathrng_frq -0.540  0.107 -0.051  0.168  0.000  0.286 -0.170
## Fields_freq -0.675  0.114  0.292  0.524  0.000 -0.021  0.112 -0.070
## Noforst_frq -0.701  0.411  0.132  0.070  0.000  0.093  0.309  0.376  0.281
```

We also check if the model is better than a null model.

```
# make the null model
null_cluster3_RA_phys=lmerTest::lmer(Freq_contact ~ (1|ID_vol) + (1|Animal_Species),
                                     data=data_glmm_RA_cluster3[which(data_glmm_RA_cluster3$ACP_contact=

# make the comparison
anova(glmm_cluster3_RA_phys, null_cluster3_RA_phys, test="chisq")
```

```
## Data: data_glmm_RA_cluster3[which(data_glmm_RA_cluster3$ACP_contact ==  ...
## Models:
## null_cluster3_RA_phys: Freq_contact ~ (1 | ID_vol) + (1 | Animal_Species)
## glmm_cluster3_RA_phys: Freq_contact ~ Village + Sexe + Relative_abundance + Hunting_freq + Fishing_f
##                       npar     AIC     BIC logLik -2*log(L)  Chisq Df
## null_cluster3_RA_phys    4 -6190.5 -6170.8 3099.2   -6198.5
## glmm_cluster3_RA_phys   13 -6177.1 -6113.1 3101.6   -6203.1 4.6142  9
##                       Pr(>Chisq)
## null_cluster3_RA_phys
## glmm_cluster3_RA_phys     0.8666
```

We visualize the fixed effects.

```
#png("plot.png", res=300, height=1100, width=1600)
sjPlot::plot_model(glmm_cluster3_RA_phys, type = "est", auto.label = FALSE,
```

```
                    vline.color = "green", sort.est = TRUE, show.values = TRUE) + ylim(-0.25,0.25)
```

```
## Scale for y is already present.
## Adding another scale for y, which will replace the existing scale.
```

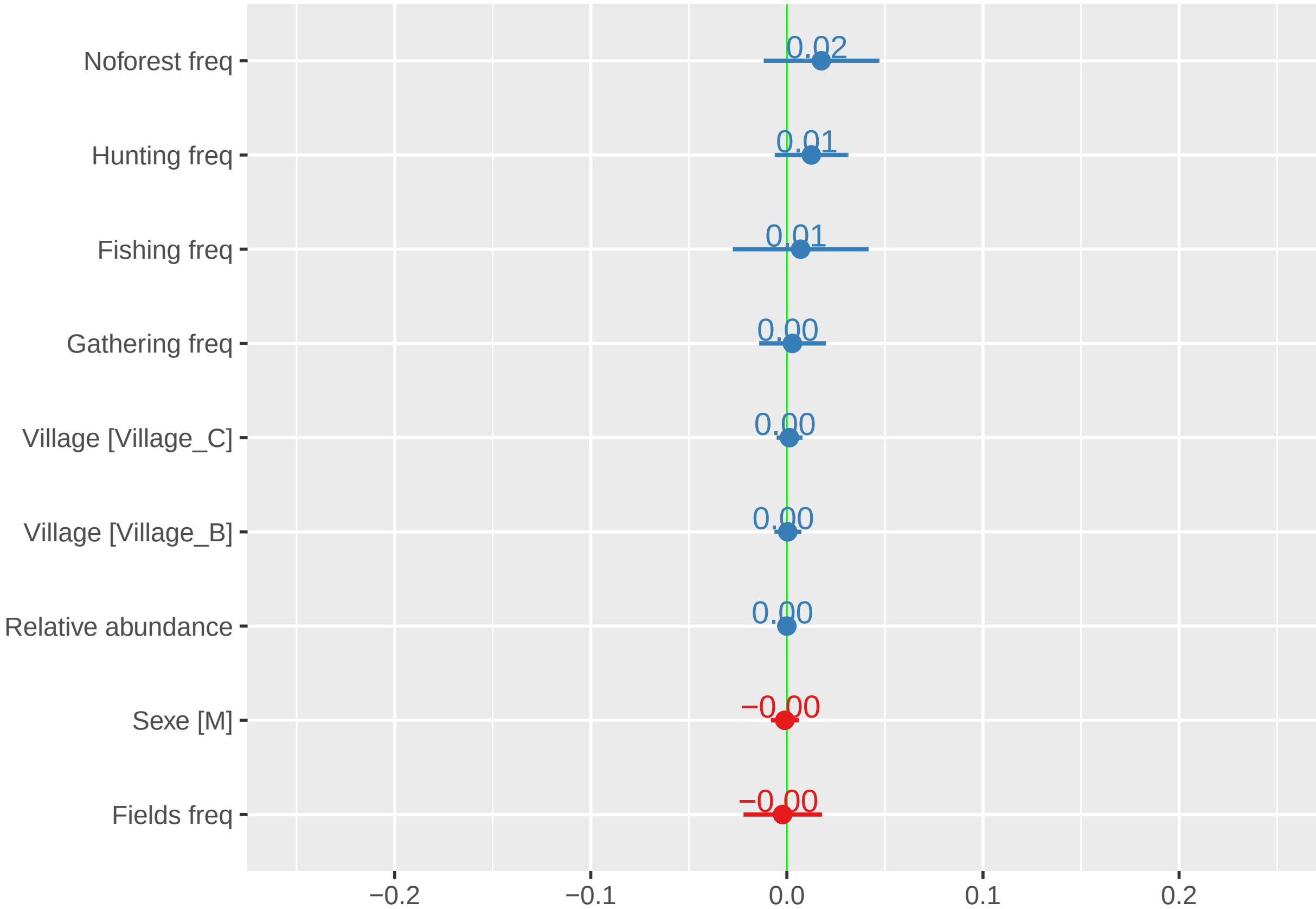


```
#dev.off()
#sjPlot::plot_model(glmm_cluster1_RA_envt, type = "pred")
#sjPlot::plot_model(glmm_cluster1_RA_envt, type = "slope")
```

We also plot the random effects

```
sjPlot::plot_model(glmm_cluster3_RA_phys, auto.label = FALSE, type = "re", # plots random effects
                   vline.color = "green", sort.est = TRUE, show.values = TRUE,
                   # add this to get the effects sorted for random effects
                   grid = FALSE)
```

```
## [[1]]
```

Random effects of ID_vol (Intercept)

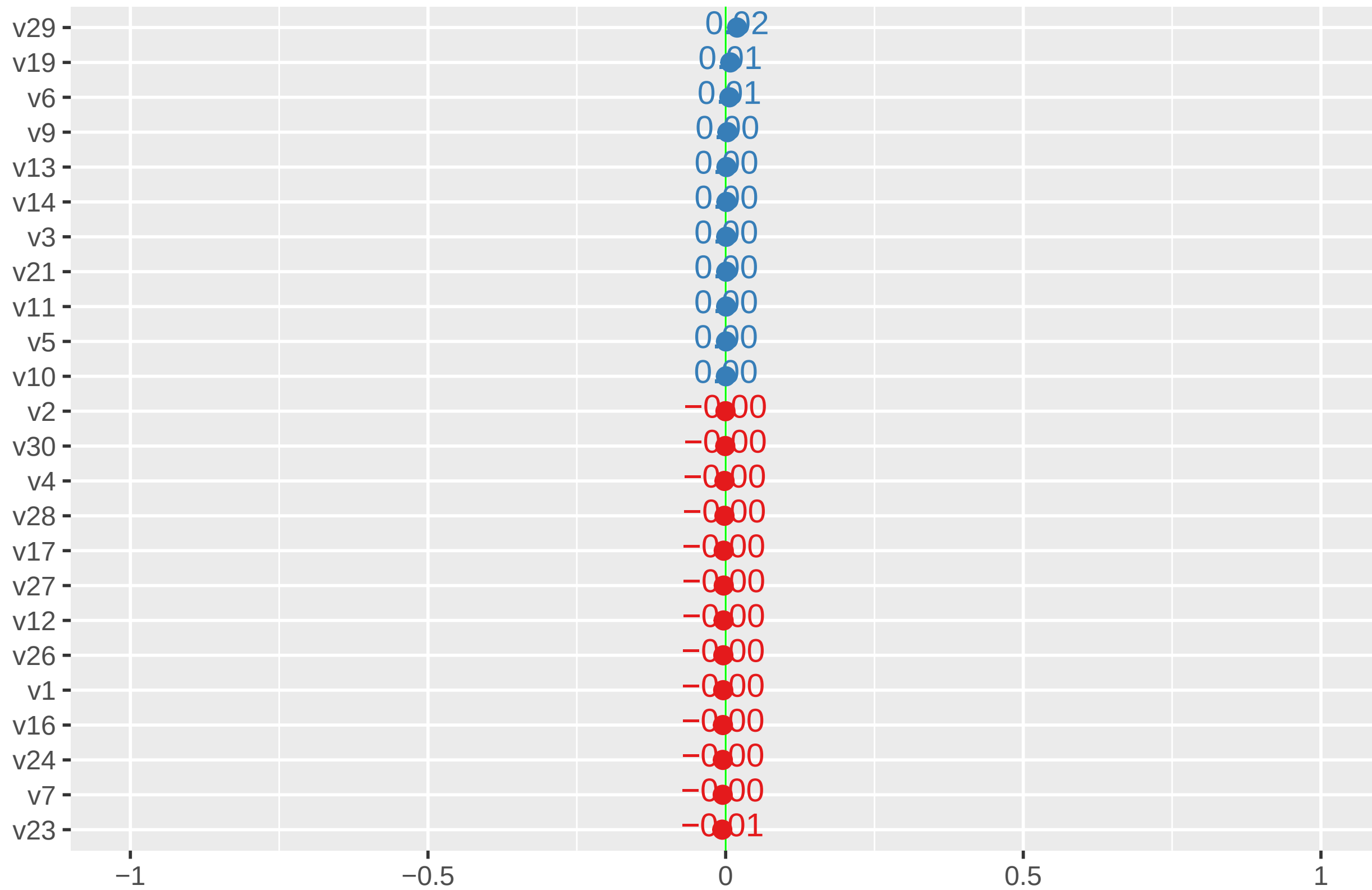


```
##
## [[2]]
```

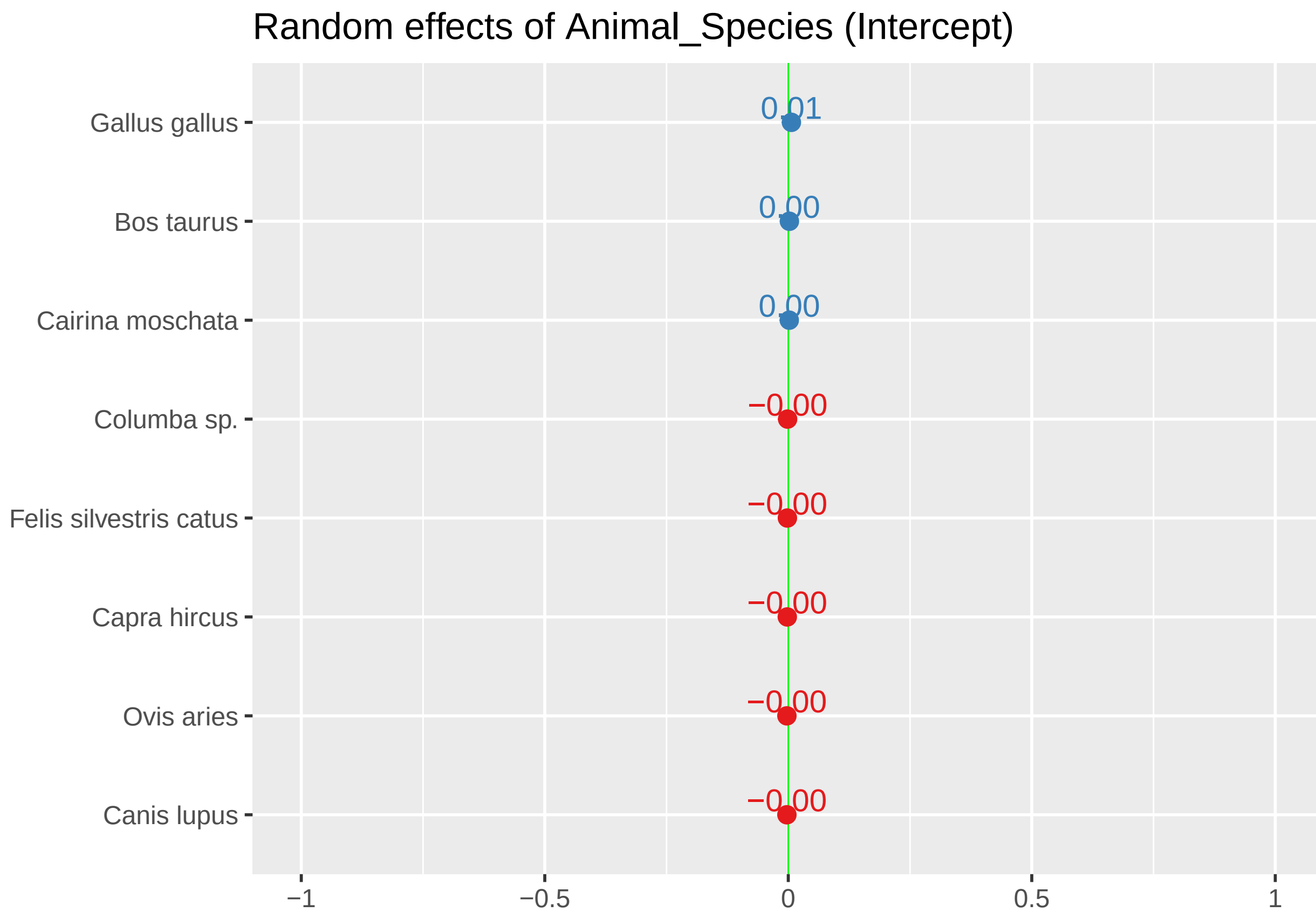

Comparing models

First, we gather all the models we generated.

```
# listing all the models
models <- c(glmm_cluster1_RA_envt, glmm_cluster1_RA_phys,
            glmm_cluster2_RA_envt, glmm_cluster2_RA_phys,
            glmm_cluster3_RA_envt, glmm_cluster3_RA_phys)
# listing all the model types
types <- c("cluster1_envt", "cluster1_phys",
           "cluster2_envt", "cluster2_phys",
           "cluster3_envt", "cluster3_phys")
```

We can visualize how the predictions align with the actual values.

```
# create an empty table for the output
data.tmp <- as.data.frame(NULL)

# for each model
for(i in 1:length(models)){
  # combine predictions and actual values
  tmp <- cbind(predict(models[[i]]), models[[i]]@frame$Freq_contact)
  # to data frame
  tmp <- as.data.frame(tmp)
  # rename the columns
  colnames(tmp) <- c("Predictions", "Actual")
  # add a column for the model name
```

```
  tmp$model = types[i]

  # combine the output with the main table
  data.tmp <- rbind(data.tmp, tmp)
}

# make a plot
ggplot(aes(x = Predictions, y = Actual), data = data.tmp) +
  geom_point(size=1, alpha = 0.2) +
  geom_line(aes(y = Predictions), color = "red") +
  facet_wrap(~model, scales = "free")
```

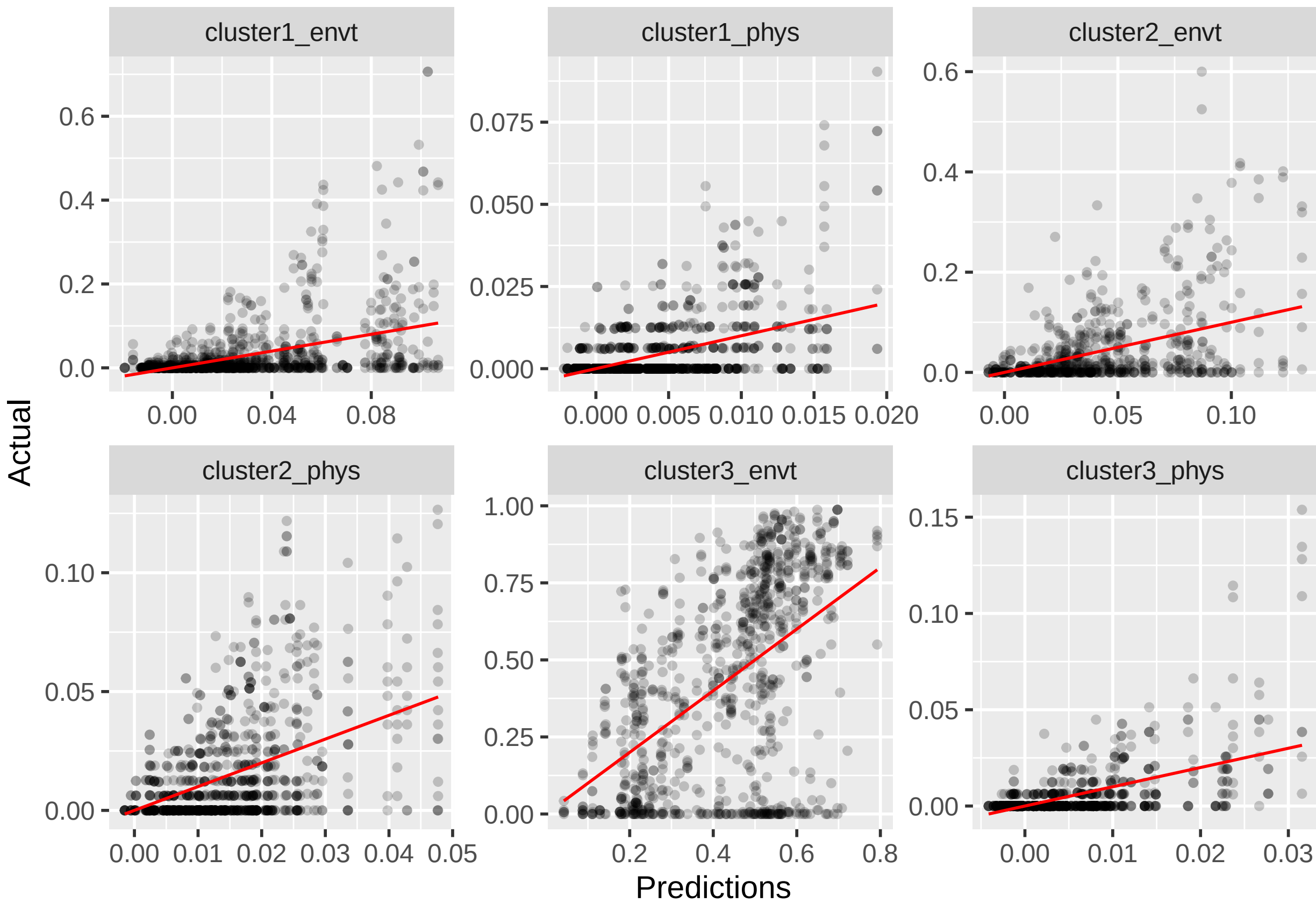

The RMSE (Root Mean Squared Error) represents the average difference between the actual values in the data and the predicted values. The lower the RMSE, the better the model. For the error rate (Scatter index), as a rule of thumb, lower than 0.1 is a good model, lower than 0.05 is a very good model.

```
# create an empty table for the output
data.tmp <- as.data.frame(NULL)

# for each model
for(i in 1:length(models)){

  # get the RMSE
  RMSE <- caret::RMSE(predict(models[[i]]), models[[i]]@frame$Freq_contact)
  RMSE <- round(RMSE, digits = 2)
  # get the mean frequency contact of the data
  mean <- mean(models[[i]]@frame$Freq_contact)
```

```
  mean <- round(mean, digits = 3)

  # get the error rate
  err.rate <- round(RMSE/mean, digits = 2)

  # combine the output with the main table
  data.tmp <- rbind(data.tmp, c(RMSE, mean, err.rate, types[i]))
}

# rename the columns
colnames(data.tmp) <- c("RMSE", "mean", "err.rate", "model")

# visualize
knitr::kable(data.tmp)
```

RMSE	mean	err.rate	model
0.06	0.025	2.4	cluster1_envt
0.01	0.004	2.5	cluster1_phys
0.07	0.041	1.71	cluster2_envt
0.02	0.015	1.33	cluster2_phys
0.28	0.431	0.65	cluster3_envt
0.01	0.005	2	cluster3_phys

The R-square (R2) represents the correlation between the actual values and the predicted values. The higher the R2, the better the model (can interpret it as a correlation coefficient). The difference between R squared and adjusted R squared value is that R squared value assumes that all the independent variables considered affect the result of the model, whereas the adjusted R squared value considers only those independent variables which actually have an effect on the performance of the model. Here we differentiate between the marginal R square, which represents the variance explained by the fixed effects and the conditional R square, which is interpreted as a variance explained by the entire model.

```
# create an empty table for the output
data.tmp <- as.data.frame(NULL)

# for each model
for(i in 1:length(models)){

  # get the R2m and the R2c
  R2m <- MuMIn::r.squaredGLMM(models[[i]])[1]
  R2c <- MuMIn::r.squaredGLMM(models[[i]])[2]
  # get the ratio
  R2m <- round(R2m/R2c, digits = 2)
  R2c <- 1 - R2m

  # use another function to compare the R2
  R2 <- round(caret::R2(predict(models[[i]]), models[[i]]@frame$Freq_contact),
              digits = 2)

  # combine the output with the main table
  data.tmp <- rbind(data.tmp, c(R2m, R2c, R2, types[i]))
}

# rename the columns
```

```r
colnames(data.tmp) <- c("ratio fixed effects", "ratio random effects", "R2 total", "model")

# visualize
knitr::kable(data.tmp)
```

ratio fixed effects	ratio random effects	R2 total	model
0.35	0.65	0.21	cluster1_envt
0.45	0.55	0.24	cluster1_phys
0.17	0.83	0.21	cluster2_envt
0.58	0.42	0.22	cluster2_phys
0.41	0.59	0.3	cluster3_envt
0.13	0.87	0.32	cluster3_phys
