# Supplementary material for: Contact in context: How land use histories, sociocultural practices, and animal profiles shape human-animal contact and zoonotic pathogen exposure in a fragmented forest of the Democratic Republic of Congo
Source: PLoS Negl Trop Dis. 2026 Oct 26;20(10):e0013823. doi: 10.1371/journal.pntd.0013823 (PMC13727040; doi:10.1371/journal.pntd.0013823)
Supplement: Supplementary file 2 [file pntd.0013823.s001.docx]

**Supplementary information 1 : Data collection tools**

**A – Human-animal contact survey** (top : activities survey ; bottom : contacts survey)

**B - Questionnaire on commensal species presence in houses and housing type**

1. Do mice (mpu) live in your house?

2. Do insectivorous bats (lempuru ngiemu) live in your house?

3. What material is the roof of your house made of: sheet metal or palm leaves?

4. What are the walls of your house made of: bricks or mud plastered on a wood structure?

**C – Semi-structured interviews guides**

Unlike questionnaires, the collection of qualitative data in semi-structured interviews took the form of free-flowing conversations guided by a set of essential questions. This allowed the researcher to ease the flow of the conversation and enabled participants to elaborate on key points related to their personal experience if they wished.

1. VILLAGE ANIMALS

1. Characterization of livestock farming owned (composition, size)
2. Livestock diseases and veterinary care
3. Animal feeding practices
4. Movement and mobility of animals within the village and its edges with forest/savanna
5. Animal handling and management, locations at night
6. Family members responsible for the animals (gender, age)
7. Consumption, slaughtering, and butchering
8. Sale and trade
9. Past livestock-keeping practices and changes over time
10. Known differences between zones and villages concerning livestock
11. Animal names, individual and collective behavior, and personality
12. Relationships with wild animals and categorization
13. History of domestication and relationships with invisible forces

2. PERCEIVED CHANGES, FOREST AND SAVANA ANIMALS

1. Social and economic changes experienced and perceived since adolescence
2. Significant dates in the person’s life or in the village’s history
3. Environmental changes experienced and perceived since adolescence (resources, seasonality, forest–savanna dynamics)
4. Perceived changes in game abundance and the reasons explaining these changes
5. Are there wild animals that come close to the village or even enter it at times?
6. Animals frequently encountered in the savanna (free list) and in the forest (free list), with a discussion of their behaviors for each species mentioned
7. Changes in hunting techniques (firearms, traps, nets) since the colonial period and during the participant’s life
8. Gender dynamics in hunting practices (women’s roles)
9. Hunting practices in the savanna
10. Trapping practices around agricultural fields
11. Slaughtering and carcass-handling practices for each technique
12. Rules for butchering and sharing game meat (who participates? how? sharing within kinship groups or hunting parties)
13. Consumption of animals found dead in the forest without known cause
14. Knowledge of wild animal diseases and their transmission to humans
15. Animals prohibited by the customary law
16. Avoidance or refusal to eat certain animals
17. Animals considered dirty or disgusting (ngeyi)
18. Animals considered intelligent
19. Areas most frequently used for hunting, and areas that are forbidden or avoided
20. Areas where animals or animal tracks are easier to encounter
21. Subsistence vs. commercial purposes for different species
22. Local taxonomies and scientific correspondances based on pictures (Kindgon, 2015)
23. Collection of proverbs or folktales, if any exist

3. COMMENSAL SPECIES LIVING IN HOUSES

1. List of unwanted animals in the house or kitchen
2. Types of contacts and reactions to their presence
3. Known reasons for their presence
4. Control techniques
5. Knowledge of diseases transmitted by these animals
6. Were their presence similar in the past?
7. Representations of these animals (repulsion, disgust, consumption)
8. Children’s relationship to these animals
9. Collection of proverbs or folktales, if any exist

4. ILLNESSES, HUMAN AND ANIMAL HEALTH

1. List of diseases that particularly affect the village
2. Dynamics/evolution of these diseases at the local scale in the last 20-30 years
3. Categorisation of the different "types" of diseases/harm/misfortunes
4. Causes of the different diseases that affect the Bateke
5. How does the disease act in the individual's body?
6. Local conceptualisation of infectious agents, vectors of disease through bodies
7. Modes of transmission of the diseases cited
8. Healing practices
9. Can animals get sick?
10. Difference between animal disease and human disease
11. Animals are known as vectors of pathogens
12. Do farm animals present any particular risks to humans?
13. Contamination between domestic and wild animals
14. Explanation/etiologies of diseases affecting livestock/wild animals
