# Supplementary material for: Contact in context: How land use histories, sociocultural practices, and animal profiles shape human-animal contact and zoonotic pathogen exposure in a fragmented forest of the Democratic Republic of Congo
Source: PLoS Negl Trop Dis. 2026 Oct 26;20(10):e0013823. doi: 10.1371/journal.pntd.0013823 (PMC13727040; doi:10.1371/journal.pntd.0013823)
Supplement: Supplementary file 3 [file pntd.0013823.s009.docx]

**PNTD-D-25-02174**

**Response to Reviewers**

We sincerely thank the Reviewers for their careful, critical read of this manuscript.

1. **Methods**

*Reviewer 1*

*The motivations underlying several methodological choices are not always clearly explained, which makes it difficult to fully understand the data collection and analysis framework.*

Thank you for signaling this lack of clarity. Below we explain our modifications for each of your points.

*Participatory data collection. The overall framework for the participatory data collection is insufficiently described. For instance, the manuscript states that “we adapted a participatory data collection tool previously developed by research team members” (L. 181), but this tool is not explained. A brief description of its structure and implementation would be important for reproducibility.*

We have provided fuller explanation of its structure and implementation on lines 189-200 and 209-223. Here we described the training of volunteers, the types of data that they collected on the data sheets, and regular follow up to ensure the robustness of the This fuller explanation, along with the supplemental files of the data sheets, should ensure reproducibility.

*In addition, the target population for data collection is not clearly defined. While it becomes apparent that the intended sampling criteria were not fully achieved (e.g., “Despite our guidelines, village chiefs selected more men than women”), the implications of this deviation are not discussed.*

The target population is described in lines 195-198 (healthy adults with regular forest and savanna activities). We acknowledge the gender imbalance in our Limitations section (lines 960-964), recognizing that it may have biased our results.

*Animal contact frequencies and clustering analysis. The motivation for the frequency model is not entirely clear. The authors state that the aim is to provide “broad insight into human exposures to animals” rather than a detailed, disaggregated analysis (L. 263). However, this raises questions about the level of aggregation: is the analysis based on total contact frequency across all species, or on species-specific interactions? This is particularly unclear given that the definition of F_v,p refers to interactions between “animal species*type of contact.” Clarification of this formulation would be helpful.*

We appreciate this critical comment and have sought to respond to it, clarifying the formula as well as its description (lines 285-299). To avoid confusion, we have deleted the first sentence of this paragraph. The analysis is based on species-specific interactions. We indicated that we do not analysis each species in detail. Instead, based on these detailed data, we conduct a categorization to obtain clusters according to frequency and type of contact with humans for each animal species.

*Clustering analysis. The rationale for using principal component analysis (PCA) and clustering is also unclear. The manuscript notes that clustering was used to avoid imposing predefined categories such as “domestic” or “wild,” yet in many cases these distinctions are relatively well established. It would therefore be helpful to better justify why a data-driven classification is preferred in this context. In addition, the description of the PCA (e.g., “performed on mean contact frequencies for each type of contact and each animal species”) is difficult to interpret. Clarifying the objective of this step—whether it is to explore correlations, reduce dimensionality, or support clustering—would improve transparency.*

We concur with R1 that this rationale for PCA and clustering, as well as the justification for avoiding pre-conceived categories of “wild” and “domesticated” could have been clearer. To that end, please see lines 308-329. Specifically, on line 319 we added that “We performed a Principal Component Analysis (PCA) to search for correlations between contact types and to reduce the number of dimensions.” Concerning our avoidance of pre-cooked categories, we contend that “Our goal was to cluster by *contact profile*, not by species biology, because we wanted to understand human exposure patterns. Predefined categories of “domestic” and “wild” obscure diverse animal ecologies and behaviors and may simultaneously impose assumptions about differences across a presumed “domestic-wild” divide. “Wild” animals on the forest edge are highly diverse in their phylogenetics, ecologies, and behaviors, and thus may bear little resemblance to one another, thus reducing the relevance of the category “wild” in assessments of contact and potential pathogenic exposures. Simultaneously, presumed differences between certain “wild” and “domesticated” mammals may be less appropriate: free-range goats, for instance, may forage in sites where small antelope also forage.”

*Influence of relative abundance, gender, and village. The assumption that proximity to villages increases the probability of contact with a given species is plausible from a human-density perspective, but not necessarily from an ecological one (i.e., it does not imply higher animal abundance near villages, except for species associated with human activity). In this context, the formulation of the relative abundance index (e.g., RA_s = sigma(1 / D_i, s) would benefit from further justification and clearer interpretation.*

We have clarified on line 331 that this proximity is “from the human perspective”.

We are not just considering “proximity to the village”. We are accounting for *distance* to the village. We weigh the value of this indicator, *distance to the village*. This allows us to weigh the relative abundance by giving more weight to indicators closer to the village, just as we have in previous published analyses of human-nonhuman primate physical contact (see Narat et al. 2018).

*Reviewer #2: (No remarks provided)*

*Reviewer #3:*

*This is an interdisciplinary work that is beyond some of my specialist skills which concern qualitative anthropology. I cannot comment on the quantitative dimension to this work. This paper develops new methods and analytical tools for understanding human animal contact, focused on three villages in Democratic Republic of Congo. Its significance is both methodological and empirical. The paper conducts cluster analysis to classify animal contact frequency to provide broad insight into regularities of human exposures to animals. This clustering eschews a priori classifications (such as domestic and wild) and its results show that such classifications would not necessarily be useful for understanding the nature and frequency of animal contact.*
*As the paper articulates, ‘This clustering constitutes a departure from human-animal contact literature, which has targeted selected animal species or pre-determined aggregated categories of animals. “Elsewhere, we have argued for abandoning analyses based on pre-conceived categories of “wild” and “captive” animals, which are neither homogeneous nor stable across diverse settings. Here we would extend our reflections, arguing that we should use category labels critically and consider how grounded experiences can shed light on alternative categorizations.” This is, as I read it, the central thrust of the paper.*

We sincerely thank Reviewer #3 for this assessment and the recognition that the clustering approach provides a means of identifying animal contacts without imposing a priori distinctions of « wild » or « domestic ».

2. **Results**

*Reviewer #1: T**he resulting dataset is rich and detailed and has the potential to be a valuable contribution for this study area. However, the conclusions implied in the title are not fully supported by the analysis presented. In particular, the manuscript does not appear to meaningfully examine land-use histories or their influence on human–animal contact patterns, nor does it provide sufficient evidence to substantiate implications regarding zoonotic spillover. Rather, the study reads primarily as a descriptive account of human–animal interactions in villages in Central Africa.*

**We have multiple responses to this assessment, which we appreciate because it has helped us to clarify several points and the overall manuscript.**

**First, we respectfully disagree that the manuscript does not “meaningfully examine land-use histories or their influence on human-animal contact patterns”.** We conducted oral historical accounts and many hours of participant-observation to collect and analyze data on land use and landscape change resulting from hunting, land cultivation, and animal production, linking these changes to historical processes affecting land use, hunting, agriculture and animal production. The Results are on lines 462-519; Discussion of these results appears on lines 866-902. This analysis is essential: without it, we would have limited insight into why contact frequency profiles look as they do.

**It is possible that R1 understands “land use history” as** reconstructed land-cover change from satellite imagery or GIS, with quantitative evidence supporting an association with contact frequency. We employ "land use history" in an anthropological, historical sense, well established in these disciplines and fully accepted in multiple others (see, for instance, Fairhead and Leach’s many publications in West Africa. We have also used this approach, including in our analyses of the histories of HIVs). Moreover, we would caution strongly against conflating “qualitative data and analysis” with “description.” Anthropology and ethnohistory have rigorous methodologies for generating and analyzing data: although such findings are presented differently from ecological analysis, they are no less rigorous or substantive than quantitative approaches.

We appreciate R1’s point in the sense that it can signal potential for misreading. We have thus have clarified what we mean by land use histories (lines 264-266) as well as multiple sections (abstract, results, and discussion) to present historical land use changes and transformations in human activities as processes that help to explain why contemporary contact patterns look as they do. See lines 497-521, 870-904.

*Reviewer #2: (no comment provided)*

*Reviewer #3: The paper needs a good edit. The authors observe in the introductory that ‘zoonotic spillovers are not only the consequence of broad scale changes, but also the result of specific human-animal-pathogen interactions at specific sites and moments.’ The methods developed here endeavour to resolve greater precision concerning these specificities. Whilst the paper focuses on gaining greater specificity in human animal contact, and considers the implications in relation to animal to human transmission, it only examines those contacts that are reflected on by inhabitants in their self-reporting and that are thus linked to everyday life.* *The study thus excludes contact with non-domestic birds, arthropods and fish, limiting analysis, and in particular it excludes consideration of symbionts and parasites even of those animals and their complex ecology. It also excludes fish. More justification and acknowledgement of the limitations that these exclusions places on the study would be appropriate.*

We thank R3 for these helpful comments. We have gone over the manuscript to clean up various errors. Concerning the focus on terrestrial human-animal contact and our exclusion of arthropods, non-domestic birds and fish, we recognize these limitations and clarified our choice of excluding arthropods in the Methods section (lines 215-218). We also acknowledge the limitation of these exclusions in the Limitation section (lines 974-977). Effectively the inclusion of arthropods, undomesticated birds, and fish would have necessitated different protocols and extended our data collection beyond our human and financial resources for this project.

*The study acknowledges several other limitations, in particular the low sample size of informants, and data collection for only 5 month of the year (thus not accounting for seasonal migrations of mammals that might include, for example, fruit bats and ground living animals). Whilst these limitations are acknowledged, the significance of the limitations are not evaluated, and whether or not these are significant for the novel methodology that the paper forwards, or the novel empirical findings.*

These are important points.

We have acknowledged in the Limitations section that undomesticated mammals living in the study area and appearing in our study engage in seasonal mobility in response to resource availability, but their movements remain highly limited (lines 968-970).

We also acknowledge the small sample size and data collection for only five months of the year in our Limitations, which could have over- or under-estimated human-animal contacts. We note that even 24-30 volunteers generated a very large quantity of data (line 959), documenting some 97,917 contact events over the five months. Moreover, it is true that five months is a limited duration, and we note this in the Limitations. But we also note that farming, hunting, and fishing continue throughout the year, although techniques change seasonally (see lines 958-963).

*The mean age of informants was 48, and this is relatively old. The paper does not examine children’s encounters with the animals, which in this region is extensive, differing with age, of course. This is also a limitation and needs to be acknowledged and reflected on.*

We thank R3 for raising this point. On the mean age, we agree that this is relatively old, and we have suggested on lines 397-399 that this imbalance reflected in part our data collection needs (to access oral historical knowledge of past land use changes and practices, we needed to speak to older adults).

The exclusion of children is indeed a shortcoming of the study. We explain in the Methods section (lines 195-198) that we excluded children and teenagers for ethical reasons. We targeted “healthy adult participants with regular activities in forest and savanna”. We acknowledge the limitations of our exclusion of children, an age group that may be highly exposed to zoonotic pathogens through their work and play (lines 955-957)

*The paper has not considered how the presence of the Bonobo project, and conservation policy and history might have shaped informant’s responses. What laws are there, and what enforcement of them might have shaped the responses?*

Lines 161-174 offer a much fuller description of MMT, the bonobo project, and legal restrictions on forest use and hunting in community forests. We situate hunting and animal population declines in the establishment of MMT in the Results (lines 490-496). We also acknowledged in the limitations the potential influence of the Bonobo conservation project on local perceptions and practices (lines 983-988).

*Concerning the findings, some of the results reported are rather obvious – that ‘those species in higher abundance and closer to villages have higher contact frequency with humans’; that hunting is not the only factor shaping human-animal contact; that changes in land use and animal husbandry can reshape the frequencies and types of contacts to specific animals’, and that human-animal contacts are shaped by human perceptions and uses of these animals.*

We agree that some patterns identified in our study may appear intuitive. Our objective, however, was to quantify and contextualize how these patterns operate within and are shaped by the specific socioecological setting of our study using granular ethnographic and quantitative data from this region. Our findings demonstrate that human-animal contact cannot be explained only by ecological availability or hunting practices alone. We show that contact patterns emerge from the interplay between ecological conditions and economic social, cultural practices/traits (e.g. preferences, avoidances) which are locally specific. We contend this finding contributes to a more nuanced understanding of exposure pathways.

*I am a little confused with the first of these results as in the methods, this finding was actually an assumption: “We assumed that the closer to a village an animal species is, the higher the probability of contact with this species.” Some clarification is needed.*

Thank you for catching this. To avoid confusion, we clarified the sentence to read “We hypothesized…” (line 335)

*The significance of the paper is found in some of the more surprising results. The paper calls into question some frequent assumptions concerning gender and the frequency of animal contact. “We found no effect of gender on contact frequency in the significant models, and no clear pattern for an effect of the village.” There is a concern, however, that the paper’s analytical methods do not resolve this gendered contact with particular species, but only with categories of species that are more or less regularly encountered. Are gender differentiated encounters with particular species within these categories? Are such specificities not important to the aim to help resolve ‘specific human-animal-pathogen interactions at specific sites and moments.’?*

These are excellent points. We have recognized from the beginning that our evidence called for a closer analysis of gender and the frequency of animal contact. That said, we think that gender needs to be addressed in a paper focusing exclusively on it. We frame the present contribution as an overview of contact profiles and analyses of factors and processes shaping them. A future piece will address these questions exclusively. Our Limitations section acknowledges these shortcomings of our gender imbalance (lines 953-957) and their possible effect on our results.

*Concerning ethics: All participants provided written informed consent, but many were not literate. A sentence on the nature of consent procedures in those circumstances would be appropriate.*

We have clarified this ethical concern in elaborating processes of obtaining informed consent, specifically for participants who were not literate (lines 381-385). Specifically, we fully explained the information notice and participant rights for all participants and obtained oral consent from those unable to provide written consent.

3. **Conclusions**

***Reviewer #1:***

*I don’t see any analysis (or data collection for that matter) on land-use, not to mention ‘historical land-use’. The discussion is also absent from this aspects. The implication to zoonosis outbreak is very little, as there is not analysis on disease side. Most of animals recorded are domestic animals as expected. It’s not clear how these contacts with chickens, ducks, goats, sheeps, pigs and dogs work as the starting point of zoonoses. It thus feel quite a stretch to have a title “Animal profiles, human activities, and land use histories shape human-animal contacts with implications for zoonotic spillover in the Democratic Republic of Congo”*

Please see our comments above concerning our data and analysis of historical land use. The data and analysis do indeed appear in the Results and the Discussion.

We thank R1 for the point about “stretching” to cover zoonotic spillover. We have moderated our claims about the implications of our analysis for zoonotic outbreak, in both the title and throughout the manuscript: we instead emphasize risk of exposure to zoonotic pathogens, not the risks of spillover. The title has been revised to: “Contact in context: How land use histories, sociocultural practices, and animal profiles shape human-animal contact and zoonotic pathogen exposure in a fragmented forest of the Democratic Republic of Congo”. We have modified language throughout the manuscript (Abstract, Introduction, Results, Discussion) to emphasize risk of zoonotic pathogenic exposure and not risk of spillover.

Moreover, contacts with domesticated animals (chickens, ducks, goats, sheep, and dogs) can indeed work as a starting point for zoonotic spillover. Rabies, for instance, is a zoonotic disease, and in Africa, dogs are the animal host. Avian influenza, Q fever, leptospirosis are also environmentally persistent pathogens carried by domesticated animals and can be transmitted to humans.

*Reviewer #2: Yes*

*Reviewer #3: Problems of interpretation**: The paper uses the cluster methods to identify species that participants rarely had physical or spatial contact with, and make the argument that “The risks of pathogenic transmission associated with these are minimal” and it thus finds that people have frequent contact with a relatively small group of animals, both wild and domesticated, that it cluster analysis identifies. It then suggests that “For this reason, surveillance of animal species in more frequently encountered animals would be a crucial measure in anticipating zoonotic spillovers and in curbing outbreaks at their source.” I am not sure that this reasoning is correct. Before focusing monitoring on commonly encountered animals, the counter argument needs to be considered, that much zoonotic transmission is very uncommon, or indeed (thankfully) exceptionally rare. It is thus not correct to infer that the risks of pathogenic transmission are associated only with animals with which participants have physical or spatial contact. Is it correct to infer that the clustering used in this paper offers potential guidance for surveillance systems, ‘to monitor only those species with which humans have most frequent physical or environmental contact.’? This is a major claim of the paper, but without considering the alternative, it may be overclaiming. There is, however, a further set of arguments that the authors might perhaps engage with, that diseases virulent to people emerge from the regular circulation of pathogens between animals and people (zoonosis and reverse zoonosis (zooanthroponosis) for which the clustering technique would indeed be useful. Equally the findings of this paper might be relevant to recent evidence concerning the importance for human immunity of the circulation of less virulent pathogens (such as Covid-19 cycling between people and rodents) that have conferred immunity benefits.*

We thank the Reviewer #3 for raising these critical points.

First, our framing overstated the implications of clustering for zoonotic risk of spillover. For this reason. we have altered the title and framed our contributions in terms of its implications concerning the risk of exposure to zoonotic pathogens, and not zoonotic spillover (see our response above).

Second, concerning the uses of clustering analysis for surveillance, we agree that contact frequency cannot be used alone to conclude the probability of pathogen spillover. We have indicated that this is a question that requires further research (line 844-846). In resource-constrained countries like DRC where broad-scale animal surveillance is not possible, targeting animal contact profiles with high physical or environmental contact could potentially make good sense. But as R3 cautions us, more research is needed.

Third, we also concur that rare contact with an animal species could result in a spillover and that frequent contact may not lead to transmission. But we also cite the history of HIVs, in which repeated physical contact alongside other factors (intrusive medical campaigns, for instance) facilitated the spillover of simian immunodeficiency viruses into humans, resulting in the adaptation of one SIV virus to human-to-human transmission and ultimately, pandemic HIV-1M. Here recurrent contact was a crucial (though not exclusive) facilitator of exposure and spillover.

Further, we entirely agree that clustering could indeed shed light on reverse zoonoses *(*zooanthroponosis*),* particularly if coupled with biological sampling. But in an already-lengthy paper, we cannot address this question and do it justice.

*A minor point: I am surprised that there are only two major commensal species (Mastomys natalensis and Pipistrella sp.) inside houses. Is this based on self-reporting. In particular, how was (the lack of) bat diversity identified.*

That only two commensal species (*Mastomys natalensis* and *Pipistrella sp*.) were reported inside houses was based on self-reporting. Participants in preliminary interviews mentioned no other species. We have clarified this point in the Methods section (line 252) and in the Limitations (lines 977-982).

4. **Editorial and Data Presentation Modifications?**

*Reviewer #1: There are numerous instances where key elements are insufficiently developed, not in terms of language or writing quality, but in the depth of explanation provided.
For example, the “Participant profiles” section consists of only a single sentence (“The profiles of participants in our data collection activities are detailed in Table 2.”, p. 12), which gives the impression of being underdeveloped. More generally, several paragraphs—and in some cases entire sections—contain only one or two sentences (e.g., “Only two of 24 volunteers raised cattle, doing so on ranches in savanna zones (040419-A, 020419-A).”, L. 388). These sections would benefit from further elaboration to provide sufficient context and interpretation.*

R1 makes a very useful point here. We therefore have added additional detail in these sections concerning cattle breeding and participant profiles. See lines 397-399, 437-443, 609-10. 879-893

*This sense of insufficient development is also evident in the figures.* *Figure 1, for instance, provides limited useful information: the map extent is very broad, while the key study area is not clearly contextualized. It would be helpful to include more localized detail, such as road networks, land use, and nearby major towns. If appropriate, indicating the locations of Village A, B, and C would further improve interpretability (while recognizing any confidentiality concerns). Additional contextual layers, such as rainfall or temperature gradients, could also be informative. More generally, satellite imagery alone is often insufficient to represent land-use patterns clearly.*

We concur and have modified the map accordingly.

*Figure 2 is also difficult to interpret, as the text elements are either too large or too dense, making them hard to read. Improving the clarity and balance of the figure design would greatly enhance readability.*

We have revised Fig. 2. We have removed the animal species names and improved the quality of the Figure.

Reviewer #2: (No Response)

Reviewer #3: (No Response)

5. **Summary and General Comments**

*Reviewer #1: This manuscript reports patterns of human–animal contact using a combination of methods, including participatory data collection, transect surveys, household surveys, and semi-structured interviews. The scope of the effort is noteworthy, involving a substantial number of participants across different components (e.g., 30 in participatory data collection and 272 in the household survey). The resulting dataset is rich and detailed, and has the potential to be a valuable contribution for this study area.*

We thank R1 for a careful, critical read of the manuscript and for multiple useful recommendations. We have undertaken extensive revisions to address all of these questions and recommendations.

*Reviewer #2: The paper examines an important question, contact as a zoonotic driver, which is investigated by means of robust mix-method research. The authors being the lead team that has introduced critical approaches to “contact” as a zoonotic driver, provide a study of 3 villages in the DRC, a key location as regards the study of disease emergence, and zoonotic diseases more broadly. The study has identified a number of animal species with which the study participants were in contact, which are clustered in 3 main classes. The study’s findings furthermore showcase how long-term socioeconomic processes inform these contact patterns, highlighting the need for more systematic examination of historically situated human-animal interactions.

I am not a life scientists so I cannot comment on the relevant methods of these paper, which however are always in dialogue and well informed by social scientific approaches to zoonosis. The social scientific design and methodology are robust, and in line with the highest standards in both medical anthropology and multispecies ethnography. Particularly welcomed is the detail involved in data collection as regards the type of contact, something that provided crucial epidemiological and ethnographic information.*

*The study can potentially make an important contribution to developing a more nuanced approach to “contact” as a zoonotic driver, which at the same challenges us to rethink the limitations and affordances of this key epidemiological notion. For this to happen the paper needs to include a broader ontextualization of the activities described in terms of the culture of the groups studies and in particular ontological and cosmological aspects of human-animal relations in these communities. This would be essential in the section titled “Historical changes in hunting and wildlife profiles” where the reader would benefit from a couple of paragraphs on these aspects. The reviewer is in agreement with the authors in capturing historically dynamic approaches to animals and not reduce human-animal interactions to a timeless cultural essentialism. However this has perhaps been taken too far, with the ontologies/cosmologies of communities (dynamic and historical as these are) being invisible to the reader. It would be particularly important to know what is the ontological/cosmological or other status of key contact-animals, so that we may understand locutions such as that mice are animals of “bad faith” [547], or the avoidance practices discussed on page 31. Similarly more context on the kinship and economic system of the local community would be necessary for us to understand practices such as meat sharing: what is the meaning of this practice, how does it distribute symbolic value or status, etc., are essential information so that we can understand what is being discussed here. The lack of such ethnographic context risks flattening the data into a naturalist ontology and a transactionalist mode of reasoning. In short the authors’ anthropological expertise needs to be employed here in a robust manner. Without this level of analysis statements such as “Our participants explained that they actively avoided certain animal species, some of which were understood to be ‘unclean’” [787-788] risk not only being not fully understood (unclean in which sense? Hygienic? Ritual? Or other?) but actually exoticise the community studied.*

We sincerely thank R2 for these very useful comments and criticisms. We took seriously R2’s remarks about the risks of invisibilising the ontolongical/cosmological status of key contact animals, and in neglecting the kin and economic systems to understanding meat sharing. Indeed, responding to these comments drove much of the revision’s expanded length.

We developed more fully the local concept of ngeyi (see lines 598-604, 709-734, 910-921) in the Results and Discussion, situating this notion of “disgusting” animals in a broader central African literature. In addition, we expanded our results and analyses of different logics of meat-sharing and its significance in these communities (lines 65—698, 905-910).

It is true that these two topics alone could justify standalone anthropological articles. But we sought to strike a balance between doing justice to our anthropological evidence and addressing the central question of this manuscript which should be of interest to multiple disciplinary perspectives.

*Reviewer #3: This research develops an innovative method for examining human-animal contact that will be important to ongoing debates about zoonotic surveillance. The results are interesting but some further reflection is needed to discern and drive home the particular significances of the findings and their limits.*

We are grateful to R3’s multiple questions and recommendations and have sought to respond to all.
