# Supplementary material for: Contact in context: How land use histories, sociocultural practices, and animal profiles shape human-animal contact and zoonotic pathogen exposure in a fragmented forest of the Democratic Republic of Congo
Source: PLoS Negl Trop Dis. 2026 Oct 26;20(10):e0013823. doi: 10.1371/journal.pntd.0013823 (PMC13727040; doi:10.1371/journal.pntd.0013823)
Supplement: Peer review history

# Peer review: Decision Letter 0

**DOI:** 10.1371/journal.pntd.0013823.r001

**Authors:** Song Liang, Marco Coral-Almeida

12 May 2026

PNTD-D-25-02174

Contact in context: Animal profiles, human activities, and land use histories shape human-animal contacts with implications for zoonotic spillover in the Democratic Republic of Congo

PLOS Neglected Tropical Diseases

Dear Dr. Giles-Vernick,

Thank you for submitting your manuscript to PLOS Neglected Tropical Diseases. After careful consideration, we feel that it has merit but does not fully meet PLOS Neglected Tropical Diseases's publication criteria as it currently stands. Therefore, we invite you to submit a revised version of the manuscript that addresses the points raised during the review process.

Please submit your revised manuscript within by Jul 11 2026 11:59PM. If you will need more time than this to complete your revisions, please reply to this message or contact the journal office at plosntds@plos.org. When you're ready to submit your revision, log on to https://www.editorialmanager.com/pntd/ and select the 'Submissions Needing Revision' folder to locate your manuscript file.

Please include the following items when submitting your revised manuscript:

* A letter that responds to each point raised by the editor and reviewer(s). You should upload this letter as a separate file labeled 'Response to Reviewers'. This file does not need to include responses to any formatting updates and technical items listed in the 'Journal Requirements' section below.

* A marked-up copy of your manuscript that highlights changes made to the original version. You should upload this as a separate file labeled 'Revised Manuscript with Track Changes'.

* An unmarked version of your revised paper without tracked changes. You should upload this as a separate file labeled 'Manuscript'.

If you would like to make changes to your financial disclosure, competing interests statement, or data availability statement, please make these updates within the submission form at the time of resubmission. Guidelines for resubmitting your figure files are available below the reviewer comments at the end of this letter.

As the corresponding author, your ORCID iD is verified in the submission system and will appear in the published article. PLOS supports the use of ORCID, and we encourage all coauthors to register for an ORCID iD and use it as well. Please encourage your coauthors to verify their ORCID iD within the submission system before final acceptance, as unverified ORCID iDs will not appear in the published article. Only the individual author can complete the verification step; PLOS staff cannot verify ORCID iDs on behalf of authors.

We look forward to receiving your revised manuscript.

Kind regards,

Marco Coral-Almeida, M.Sc., Ph.D.

Academic Editor

PLOS Neglected Tropical Diseases

Song Liang

Section Editor

PLOS Neglected Tropical Diseases

Shaden Kamhawi

co-Editor-in-Chief

PLOS Neglected Tropical Diseases

orcid.org/0000-0003-4304-636XX

Paul Brindley

co-Editor-in-Chief

PLOS Neglected Tropical Diseases

orcid.org/0000-0003-1765-0002

**Journal Requirements:**

1) We note that you have included Response to Journal Requirements in the cover letter. Please remove your Response to Journal Requirements from your Cover Letter. 2) We do not publish any copyright or trademark symbols that usually accompany proprietary names, eg ©,  ®, or TM  (e.g. next to drug or reagent names). Therefore please remove all instances of trademark/copyright symbols throughout the text, including: - ® on page: 6. 3) Please upload all main figures as separate Figure files in .tif or .eps format. For more information about how to convert and format your figure files please see our guidelines:  https://journals.plos.org/plosntds/s/figures 4) We have noticed that you have uploaded Supporting Information files, but you have not included a list of legends. Please add a full list of legends for your Supporting Information files after the references list. 5) Some material included in your submission may be copyrighted. According to PLOS’s copyright policy, authors who use figures or other material (e.g., graphics, clipart, maps) from another author or copyright holder must demonstrate or obtain permission to publish this material under the Creative Commons Attribution 4.0 International (CC BY 4.0) License used by PLOS journals. Please closely review the details of PLOS’s copyright requirements here: PLOS Licenses and Copyright. If you need to request permissions from a copyright holder, you may use PLOS's Copyright Content Permission form. Please respond directly to this email and provide any known details concerning your material's license terms and permissions required for reuse, even if you have not yet obtained copyright permissions or are unsure of your material's copyright compatibility. Once you have responded and addressed all other outstanding technical requirements, you may resubmit your manuscript within Editorial Manager.  Potential Copyright Issues: i) Figure 1. Please (a) provide a direct link to the base layer of the map (i.e., the country or region border shape) and ensure this is also included in the figure legend; and (b) provide a link to the terms of use / license information for the base layer image or shapefile. We cannot publish proprietary or copyrighted maps (e.g. Google Maps, Mapquest) and the terms of use for your map base layer must be compatible with our CC BY 4.0 license. Note: if you created the map in a software program like R or ArcGIS, please locate and indicate the source of the basemap shapefile onto which data has been plotted. If your map was obtained from a copyrighted source please amend the figure so that the base map used is from an openly available source. Alternatively, please provide explicit written permission from the copyright holder granting you the right to publish the material under our CC BY 4.0 license. If you are unsure whether you can use a map or not, please do reach out and we will be able to help you. The following websites are good examples of where you can source open access or public domain maps: * U.S. Geological Survey (USGS) - All maps are in the public domain. (http://www.usgs.gov) * PlaniGlobe - All maps are published under a Creative Commons license so please cite u201cPlaniGlobe, http://www.planiglobe.com, CC BY 2.0u201d in the image credit after the caption. (http://www.planiglobe.com/?lang=enl) * Natural Earth - All maps are public domain. (http://www.naturalearthdata.com/about/terms-of-use/). 6) We note that you have indicated that there are restrictions to data sharing for this study. For studies involving human research participant data or other sensitive data, we encourage authors to share de-identified or anonymized data. However, when data cannot be publicly shared for ethical reasons, we allow authors to make their data sets available upon request. For information on unacceptable data access restrictions, please see https://journals.plos.org/plosntds/s/data-availability#loc-unacceptable-data-access-restrictions. Before we proceed with your manuscript, please address the following prompts: a) If there are ethical or legal restrictions on sharing a de-identified data set, please explain them in detail (e.g., data contain potentially identifying or sensitive patient information, data are owned by a third-party organization, etc.) and who has imposed them (e.g., a Research Ethics Committee or Institutional Review Board, etc.). Please also provide contact information for a data access committee, ethics committee, or other institutional body to which data requests may be sent. b) If there are no restrictions, please upload the minimal anonymized data set necessary to replicate your study findings to a stable, public repository and provide us with the relevant URLs, DOIs, or accession numbers. Please see http://www.bmj.com/content/340/bmj.c181.long for guidelines on how to de-identify and prepare clinical data for publication. For a list of recommended repositories, please see https://journals.plos.org/plosone/s/recommended-repositories. You also have the option of uploading the data as Supporting Information files, but we would recommend depositing data directly to a data repository if possible. Please update your Data Availability statement in the submission form accordingly.  7) Please amend your detailed Financial Disclosure statement. This is published with the article. It must therefore be completed in full sentences and contain the exact wording you wish to be published. 1) State the initials, alongside each funding source, of each author to receive each grant. For example: "This work was supported by the National Institutes of Health (####### to AM; ###### to CJ) and the National Science Foundation (###### to AM)." 2) State what role the funders took in the study. If the funders had no role in your study, please state: "The funders had no role in study design, data collection and analysis, decision to publish, or preparation of the manuscript.". 8) Please ensure that the funders and grant numbers match between the Financial Disclosure field and the Funding Information tab in your submission form.  Note: If the reviewer comments include a recommendation to cite specific previously published works, please review and evaluate these publications to determine whether they are relevant and should be cited. There is no requirement to cite these works unless the editor has indicated otherwise.

**Reviewers' Comments:**

Reviewer's Responses to Questions

**Key Review Criteria Required for Acceptance?**

As you describe the new analyses required for acceptance, please consider the following:

**Methods**

-Are the objectives of the study clearly articulated with a clear testable hypothesis stated?

-Is the study design appropriate to address the stated objectives?

-Is the population clearly described and appropriate for the hypothesis being tested?

-Is the sample size sufficient to ensure adequate power to address the hypothesis being tested?

-Were correct statistical analysis used to support conclusions?

-Are there concerns about ethical or regulatory requirements being met?

Reviewer #1: The motivations underlying several methodological choices are not always clearly explained, which makes it difficult to fully understand the data collection and analysis framework.

Participatory data collection. The overall framework for the participatory data collection is insufficiently described. For instance, the manuscript states that “we adapted a participatory data collection tool previously developed by research team members” (L. 181), but this tool is not explained. A brief description of its structure and implementation would be important for reproducibility.

In addition, the target population for data collection is not clearly defined. While it becomes apparent that the intended sampling criteria were not fully achieved (e.g., “Despite our guidelines, village chiefs selected more men than women”), the implications of this deviation are not discussed.

Animal contact frequencies and clustering analysis. The motivation for the frequency model is not entirely clear. The authors state that the aim is to provide “broad insight into human exposures to animals” rather than a detailed, disaggregated analysis (L. 263). However, this raises questions about the level of aggregation: is the analysis based on total contact frequency across all species, or on species-specific interactions? This is particularly unclear given that the definition of F_v,p refers to interactions between “animal species*type of contact.” Clarification of this formulation would be helpful.

Clustering analysis. The rationale for using principal component analysis (PCA) and clustering is also unclear. The manuscript notes that clustering was used to avoid imposing predefined categories such as “domestic” or “wild,” yet in many cases these distinctions are relatively well established. It would therefore be helpful to better justify why a data-driven classification is preferred in this context. In addition, the description of the PCA (e.g., “performed on mean contact frequencies for each type of contact and each animal species”) is difficult to interpret. Clarifying the objective of this step—whether it is to explore correlations, reduce dimensionality, or support clustering—would improve transparency.

Influence of relative abundance, gender, and village. The assumption that proximity to villages increases the probability of contact with a given species is plausible from a human-density perspective, but not necessarily from an ecological one (i.e., it does not imply higher animal abundance near villages, except for species associated with human activity). In this context, the formulation of the relative abundance index (e.g., RA_s = sigma(1 / D_i, s) would benefit from further justification and clearer interpretation.

Reviewer #2: Yes

Reviewer #3: This is an interdisciplinary work that is beyond some of my specialist skills which concern qualitative anthropology. I cannot comment on the quantitative dimension to this work. This paper develops new methods and analytical tools for understanding human animal contact, focused on three villages in Democratic Republic of Congo. It’s significance is both methodological and empirical. The paper conducts cluster analysis to classify animal contact frequency to provide broad insight into regularities of human exposures to animals. This clustering eschews a priori classifications (such as domestic and wild) and its results show that such classifications would not necessarily be useful for understanding the nature and frequency of animal contact.

As the paper articulates, ‘This clustering constitutes a departure from human-animal contact literature, which has targeted selected animal species or pre-determined aggregated categories of animals. “Elsewhere, we have argued for abandoning analyses based on pre-conceived categories of “wild” and “captive” animals, which are neither homogeneous nor stable across diverse settings. Here we would extend our reflections, arguing that we should use category labels critically and consider how grounded experiences can shed light on alternative categorizations.” This is, as I read it, the central thrust of the paper.

**********

**Results**

-Does the analysis presented match the analysis plan?

-Are the results clearly and completely presented?

-Are the figures (Tables, Images) of sufficient quality for clarity?

Reviewer #1: The resulting dataset is rich and detailed, and has the potential to be a valuable contribution for this study area. However, the conclusions implied in the title are not fully supported by the analysis presented. In particular, the manuscript does not appear to meaningfully examine land-use histories or their influence on human–animal contact patterns, nor does it provide sufficient evidence to substantiate implications regarding zoonotic spillover. Rather, the study reads primarily as a descriptive account of human–animal interactions in villages in Central Africa.

Reviewer #2: Yes

Reviewer #3: The paper needs a good edit.

The authors observe in the introductory that ‘zoonotic spillovers are not only the consequence of broad scale changes, but also the result of specific human-animal-pathogen interactions at specific sites and moments.’ The methods developed here endeavour to resolve greater precision concerning these specificities. Whilst the paper focuses on gaining greater specificity in human animal contact, and considers the implications in relation to animal to human transmission, it only examines those contacts that are reflected on by inhabitants in their self-reporting and that are thus linked to everyday life. The study thus excludes contact with non-domestic birds, arthropods and fish, limiting analysis, and in particular it excludes consideration of symbionts and parasites even of those animals and their complex ecology. It also excludes fish. More justification and acknowledgement of the limitations that these exclusions places on the study would be appropriate.

The study acknowledges several other limitations, in particular the low sample size of informants, and data collection for only 5 month of the year (thus not accounting for seasonal migrations of mammals that might include, for example, fruit bats and ground living animals). Whilst these limitations are acknowledged, the significance of the limitations are not evaluated, and whether or not these are significant for the novel methodology that the paper forwards, or the novel empirical findings.

The mean age of informants was 48, and this is relatively old. The paper does not examine children’s encounters with the animals, which in this region is extensive, differing with age, of course. This is also a limitation and needs to be acknowledged and reflected on.

The paper has not considered how the presence of the Bonobo project, and conservation policy and history might have shaped informant’s responses. What laws are there, and what enforcement of them might have shaped the responses?

Concerning the findings, some of the results reported are rather obvious – that ‘those species in higher abundance and closer to villages have higher contact frequency with humans’; that hunting is not the only factor shaping human-animal contact; that changes in land use and animal husbandry can reshape the frequencies and types of contacts to specific animals’, and that human-animal contacts are shaped by human perceptions and uses of these animals.

I am a little confused with the first of these results as in the methods, this finding was actually an assumption: “We assumed that the closer to a village an animal species is, the higher the probability of contact with this species.” Some clarification is needed.

The significance of the paper is found in some of the more surprising results. The paper calls into question some frequent assumptions concerning gender and the frequency of animal contact. “We found no effect of gender on contact frequency in the significant models, and no clear pattern for an effect of the village.” There is a concern, however, that the paper’s analytical methods do not resolve this gendered contact with particular species, but only with categories of species that are more or less regularly encountered. Are gender differentiated encounters with particular species within these categories? Are such specificities not important to the aim to help resolve ‘specific human-animal-pathogen interactions at specific sites and moments.’?

Concerning ethics:

All participants provided written informed consent, but many were not literate. A sentence on the nature of consent procedures in those circumstances would be appropriate.

**********

**Conclusions**

-Are the conclusions supported by the data presented?

-Are the limitations of analysis clearly described?

-Do the authors discuss how these data can be helpful to advance our understanding of the topic under study?

-Is public health relevance addressed?

Reviewer #1: I don’t see any analysis (or data collection for that matter) on land-use, not to mention ‘historical land-use’. The discussion is also absent from this aspects. The implication to zoonosis outbreak is very little, as there is not analysis on disease side. Most of animals recorded are domestic animals as expected. It’s not clear how these contacts with chickens, ducks, goats, sheeps, pigs and dogs work as the starting point of zoonoses. It thus feel quite a stretch to have a title “Animal profiles, human activities, and land use histories shape human-animal contacts with implications for zoonotic spillover in the Democratic Republic of Congo”

Reviewer #2: Yes

Reviewer #3: Problems of interpretation

The paper uses the cluster methods to identify species that participants rarely had physical or spatial contact with, and make the argument that “The risks of pathogenic transmission associated with these are minimal” and it thus finds that people have frequent contact with a relatively small group of animals, both wild and domesticated, that it cluster analysis identifies. It then suggests that “For this reason, surveillance of animal species in more frequently encountered animals would be a crucial measure in anticipating zoonotic spillovers and in curbing outbreaks at their source.” I am not sure that this reasoning is correct. Before focusing monitoring on commonly encountered animals, the counter argument needs to be considered, that much zoonotic transmission is very uncommon, or indeed (thankfully) exceptionally rare. It is thus not correct to infer that the risks of pathogenic transmission are associated only with animals with which participants have physical or spatial contact. Is it correct to infer that the clustering used in this paper offers potential guidance for surveillance systems, ‘to monitor only those species with which humans have most frequent physical or environmental contact.’? This is a major claim of the paper, but without considering the alternative, it may be overclaiming. There is, however, a further set of arguments that the authors might perhaps engage with, that diseases virulent to people emerge from the regular circulation of pathogens between animals and people (zoonosis and reverse zoonosis (zooanthroponosis) for which the clustering technique would indeed be useful. Equally the findings of this paper might be relevant to recent evidence concerning the importance for human immunity of the circulation of less virulent pathogens (such as Covid-19 cycling between people and rodents) that have conferred immunity benefits.

A minor point: I am surprised that there are only two major commensal species (Mastomys natalensis and Pipistrella sp.) inside houses. Is this based on self-reporting. In particular, how was (the lack of) bat diversity identified.

**********

**Editorial and Data Presentation Modifications?**

Use this section for editorial suggestions as well as relatively minor modifications of existing data that would enhance clarity. If the only modifications needed are minor and/or editorial, you may wish to recommend “Minor Revision” or “Accept”.

Reviewer #1: There are numerous instances where key elements are insufficiently developed, not in terms of language or writing quality, but in the depth of explanation provided.

For example, the “Participant profiles” section consists of only a single sentence (“The profiles of participants in our data collection activities are detailed in Table 2.”, p. 12), which gives the impression of being underdeveloped. More generally, several paragraphs—and in some cases entire sections—contain only one or two sentences (e.g., “Only two of 24 volunteers raised cattle, doing so on ranches in savanna zones (040419-A, 020419-A).”, L. 388). These sections would benefit from further elaboration to provide sufficient context and interpretation.

This sense of insufficient development is also evident in the figures. Figure 1, for instance, provides limited useful information: the map extent is very broad, while the key study area is not clearly contextualized. It would be helpful to include more localized detail, such as road networks, land use, and nearby major towns. If appropriate, indicating the locations of Village A, B, and C would further improve interpretability (while recognizing any confidentiality concerns). Additional contextual layers, such as rainfall or temperature gradients, could also be informative. More generally, satellite imagery alone is often insufficient to represent land-use patterns clearly.

Figure 2 is also difficult to interpret, as the text elements are either too large or too dense, making them hard to read. Improving the clarity and balance of the figure design would greatly enhance readability.

Reviewer #2: (No Response)

Reviewer #3: (No Response)

**********

**Summary and General Comments**

Use this section to provide overall comments, discuss strengths/weaknesses of the study, novelty, significance, general execution and scholarship. You may also include additional comments for the author, including concerns about dual publication, research ethics, or publication ethics. If requesting major revision, please articulate the new experiments that are needed.

Reviewer #1: This manuscript reports patterns of human–animal contact using a combination of methods, including participatory data collection, transect surveys, household surveys, and semi-structured interviews. The scope of the effort is noteworthy, involving a substantial number of participants across different components (e.g., 30 in participatory data collection and 272 in the household survey). The resulting dataset is rich and detailed, and has the potential to be a valuable contribution for this study area.

Reviewer #2: The paper examines an important question, contact as a zoonotic driver, which is investigated by means of robust mix-method research. The authors being the lead team that has introduced critical approaches to “contact” as a zoonotic driver, provide a study of 3 villages in the DRC, a key location as regards the study of disease emergence, and zoonotic diseases more broadly. The study has identified a number of animal species with which the study participants were in contact, which are clustered in 3 main classes. The study’s findings furthermore showcase how long-term socioeconomic processes inform these contact patterns, highlighting the need for more systematic examination of historically situated human-animal interactions.

I am not a life scientists so I cannot comment on the relevant methods of these paper, which however are always in dialogue and well informed by social scientific approaches to zoonosis. The social scientific design and methodology are robust, and in line with the highest standards in both medical anthropology and multispecies ethnography. Particularly welcomed is the detail involved in data collection as regards the type of contact, something that provided crucial epidemiological and ethnographic information.

The study can potentially make an important contribution to developing a more nuanced approach to “contact” as a zoonotic driver, which at the same challenges us to rethink the limitations and affordances of this key epidemiological notion. For this to happen the paper needs to include a broader contextualisation of the activities described in terms of the culture of the groups studies and in particular ontological and cosmological aspects of human-animal relations in these communities. This would be essential in the section titled “Historical changes in hunting and wildlife profiles” where the reader would benefit from a couple of paragraphs on these aspects. The reviewer is in agreement with the authors in capturing historically dynamic approaches to animals and not reduce human-animal interactions to a timeless cultural essentialism. However this has perhaps been taken too far, with the ontologies/cosmologies of communities (dynamic and historical as these are) being invisible to the reader. It would be particularly important to know what is the ontological/cosmological or other status of key contact-animals, so that we may understand locutions such as that mice are animals of “bad faith” [547], or the avoidance practices discussed on page 31. Similarly more context on the kinship and economic system of the local community would be necessary for us to understand practices such as meat sharing: what is the meaning of this practice, how does it distribute symbolic value or status, etc., are essential information so that we can understand what is being discussed here. The lack of such ethnographic context risks flattening the data into a naturalist ontology and a transactionalist mode of reasoning. In short the authors’ anthropological expertise needs to be employed here in a robust manner. Without this level of analysis statements such as “Our participants explained that they actively avoided certain animal species, some of which were understood to be ‘unclean’” [787-788] risk not only being not fully understood (unclean in which sense? Hygienic? Ritual? Or other?) but actually exoticise the community studied.

Reviewer #3: This research develops an innovative method for examining human-animal contact that will be important to ongoing debates about zoonotic surveillance. The results are interesting but some further reflection is needed to discern and drive home the particular significances of the findings and their limits

**********

PLOS authors have the option to publish the peer review history of their article (what does this mean?). If published, this will include your full peer review and any attached files.

If you choose “no”, your identity will remain anonymous but your review may still be made public.

**Do you want your identity to be public for this peer review?**  For information about this choice, including consent withdrawal, please see our Privacy Policy.

Reviewer #1: No

Reviewer #2: No

Reviewer #3: No

[NOTE: If reviewer comments were submitted as an attachment file, they will be attached to this email and accessible via the submission site. Please log into your account, locate the manuscript record, and check for the action link "View Attachments". If this link does not appear, there are no attachment files.]

**Figure resubmission:**

While revising your submission, we strongly recommend that you use PLOS’s NAAS tool (https://ngplosjournals.pagemajik.ai/artanalysis) to test your figure files. NAAS can convert your figure files to the TIFF file type and meet basic requirements (such as print size, resolution), or provide you with a report on issues that do not meet our requirements and that NAAS cannot fix.

After uploading your figures to PLOS’s NAAS tool - https://ngplosjournals.pagemajik.ai/artanalysis, NAAS will process the files provided and display the results in the "Uploaded Files" section of the page as the processing is complete. If the uploaded figures meet our requirements (or NAAS is able to fix the files to meet our requirements), the figure will be marked as "fixed" above. If NAAS is unable to fix the files, a red "failed" label will appear above. When NAAS has confirmed that the figure files meet our requirements, please download the file via the download option, and include these NAAS processed figure files when submitting your revised manuscript.

**Reproducibility:**

To enhance the reproducibility of your results, we recommend that authors of applicable studies deposit laboratory protocols in protocols.io, where a protocol can be assigned its own identifier (DOI) such that it can be cited independently in the future. Additionally, PLOS ONE offers an option to publish peer-reviewed clinical study protocols. Read more information on sharing protocols at https://plos.org/protocols?utm_medium=editorial-email&utm_source=authorletters&utm_campaign=protocols

# Author comment: Author response to Decision Letter 1

**DOI:** 10.1371/journal.pntd.0013823.r002

3 Sep 2026

Attachment Submitted filename: Contact in context Response to reviewers.docx

# Peer review: Decision Letter 1

**DOI:** 10.1371/journal.pntd.0013823.r003

**Authors:** Song Liang, Marco Coral-Almeida

12 Oct 2026

Dear Dr. Giles-Vernick,

We are pleased to inform you that your manuscript 'Contact in context: How land use histories, sociocultural practices, and animal profiles shape human-animal contact and zoonotic pathogen exposure in a fragmented forest of the Democratic Republic of Congo' has been provisionally accepted for publication in PLOS Neglected Tropical Diseases.

Before your manuscript can be formally accepted you will need to complete some formatting changes, which you will receive in a follow up email. A member of our team will be in touch with a set of requests.

Please note that your manuscript will not be scheduled for publication until you have made the required changes, so a swift response is appreciated.

IMPORTANT: The editorial review process is now complete. PLOS will only permit corrections to spelling, formatting or significant scientific errors from this point onwards. Requests for major changes, or any which affect the scientific understanding of your work, will cause delays to the publication date of your manuscript.

Should you, your institution's press office or the journal office choose to press release your paper, you will automatically be opted out of early publication. We ask that you notify us now if you or your institution is planning to press release the article. All press must be co-ordinated with PLOS.

Thank you again for supporting Open Access publishing; we are looking forward to publishing your work in PLOS Neglected Tropical Diseases.

Best regards,

Marco Coral-Almeida, M.Sc., Ph.D.

Academic Editor

PLOS Neglected Tropical Diseases

Song Liang

Section Editor

PLOS Neglected Tropical Diseases

Shaden Kamhawi

co-Editor-in-Chief

PLOS Neglected Tropical Diseases

orcid.org/0000-0003-4304-636XX

Paul Brindley

co-Editor-in-Chief

PLOS Neglected Tropical Diseases

orcid.org/0000-0003-1765-0002

***********************************************************

Reviewer's Responses to Questions

**Key Review Criteria Required for Acceptance?**

As you describe the new analyses required for acceptance, please consider the following:

**Methods**

-Are the objectives of the study clearly articulated with a clear testable hypothesis stated?

-Is the study design appropriate to address the stated objectives?

-Is the population clearly described and appropriate for the hypothesis being tested?

-Is the sample size sufficient to ensure adequate power to address the hypothesis being tested?

-Were correct statistical analysis used to support conclusions?

-Are there concerns about ethical or regulatory requirements being met?

Reviewer #1: Yes. The authors have satisfactorily addressed the comments and concerns raised in my previous review. I have no further comments.

Reviewer #2: No change of evaluation from prior review

Reviewer #3: This is now very clear and well expressed. My original cocnerns have been addressed or reported in limitations

**********

**Results**

-Does the analysis presented match the analysis plan?

-Are the results clearly and completely presented?

-Are the figures (Tables, Images) of sufficient quality for clarity?

Reviewer #1: Yes. The authors have satisfactorily addressed the comments and concerns raised in my previous review. I have no further comments.

Reviewer #2: No change of evaluation from prior review

Reviewer #3: The results and analysis is reported clearly.

**********

**Conclusions**

-Are the conclusions supported by the data presented?

-Are the limitations of analysis clearly described?

-Do the authors discuss how these data can be helpful to advance our understanding of the topic under study?

-Is public health relevance addressed?

Reviewer #1: Yes. The authors have satisfactorily addressed the comments and concerns raised in my previous review. I have no further comments.

Reviewer #2: No change of evaluation from prior review

Reviewer #3: These are now clear and qualified.

**********

**Editorial and Data Presentation Modifications?**

Use this section for editorial suggestions as well as relatively minor modifications of existing data that would enhance clarity. If the only modifications needed are minor and/or editorial, you may wish to recommend “Minor Revision” or “Accept”.

Reviewer #1: Yes. The authors have satisfactorily addressed the comments and concerns raised in my previous review. I have no further comments.

Reviewer #2: No change of evaluation from prior review

Reviewer #3: (No Response)

**********

**Summary and General Comments**

Use this section to provide overall comments, discuss strengths/weaknesses of the study, novelty, significance, general execution and scholarship. You may also include additional comments for the author, including concerns about dual publication, research ethics, or publication ethics. If requesting major revision, please articulate the new experiments that are needed.

Reviewer #1: Yes. The authors have satisfactorily addressed the comments and concerns raised in my previous review. I have no further comments.

Reviewer #2: The authors have responded to R3 comments in a systematic and convincing manner, with revisions made to the article addressing and resolving all points made. I am satisfied by these revisions and have no further revisions to suggest. As far as the social scientific aspects of this article are concerned, the article can be published without further revisions.

Reviewer #3: A very good resubmission that has taken on board earlier comments and has developed a clear and useful papaer.

**********

PLOS authors have the option to publish the peer review history of their article (what does this mean?). If published, this will include your full peer review and any attached files.

If you choose “no”, your identity will remain anonymous but your review may still be made public.

**Do you want your identity to be public for this peer review?**  For information about this choice, including consent withdrawal, please see our Privacy Policy.

Reviewer #1: No

Reviewer #2: No

Reviewer #3: No

# Editor report: Acceptance letter

**DOI:** 10.1371/journal.pntd.0013823.r004

**Authors:** Song Liang, Marco Coral-Almeida

Dear Dr Giles-Vernick,

We are delighted to inform you that your manuscript, "Contact in context: How land use histories, sociocultural practices, and animal profiles shape human-animal contact and zoonotic pathogen exposure in a fragmented forest of the Democratic Republic of Congo," has been formally accepted for publication in PLOS Neglected Tropical Diseases.

We have now passed your article onto the PLOS Production Department who will complete the rest of the publication process. All authors will receive a confirmation email upon publication.

The corresponding author will soon be receiving a typeset proof for review, to ensure errors have not been introduced during production. Please review the PDF proof of your manuscript carefully, as this is the last chance to correct any scientific or type-setting errors. Please note that major changes, or those which affect the scientific understanding of the work, will likely cause delays to the publication date of your manuscript. Note: Proofs for Front Matter articles (Editorial, Viewpoint, Symposium, Review, etc...) are generated on a different schedule and may not be made available as quickly.

Soon after your final files are uploaded, the early version of your manuscript will be published online unless you opted out of this process. The date of the early version will be your article's publication date. The final article will be published to the same URL, and all versions of the paper will be accessible to readers.

For Research Articles, you will receive an invoice from PLOS for your publication fee after your manuscript has reached the completed accept phase. If you receive an email requesting payment before acceptance or for any other service, this may be a phishing scheme. Learn how to identify phishing emails and protect your accounts at https://explore.plos.org/phishing.

Thank you again for supporting open-access publishing; we are looking forward to publishing your work in PLOS Neglected Tropical Diseases.

Best regards,

Shaden Kamhawi

co-Editor-in-Chief

PLOS Neglected Tropical Diseases

Paul Brindley

co-Editor-in-Chief

PLOS Neglected Tropical Diseases
